# Safety and Tolerability of Conserved Region Vaccines Vectored by Plasmid DNA, Simian Adenovirus and Modified Vaccinia Virus Ankara Administered to Human Immunodeficiency Virus Type 1-Uninfected Adults in a Randomized, Single-Blind Phase I Trial

**DOI:** 10.1371/journal.pone.0101591

**Published:** 2014-07-09

**Authors:** Emma-Jo Hayton, Annie Rose, Umar Ibrahimsa, Mariarosaria Del Sorbo, Stefania Capone, Alison Crook, Antony P. Black, Lucy Dorrell, Tomáš Hanke

**Affiliations:** 1 Centre for Clinical Vaccinology and Tropical Medicine, The Jenner Institute, University of Oxford, Churchill Hospital, Oxford, United Kingdom; 2 Okairos, Rome, Italy; 3 MRC Human Immunology Unit, Weatherall Institute of Molecular Medicine, University of Oxford, The John Radcliffe, Oxford, United Kingdom; 4 Nuffield Department of Medicine Research Building, University of Oxford, Oxford, United Kingdom; 5 The Jenner Institute Laboratories, University of Oxford, Old Road Campus Research Building, Oxford, United Kingdom; University of Alabama, United States of America

## Abstract

**Trial Design:**

HIV-1 vaccine development has advanced slowly due to viral antigenic diversity, poor immunogenicity and recently, safety concerns associated with human adenovirus serotype-5 vectors. To tackle HIV-1 variation, we designed a unique T-cell immunogen HIVconsv from functionally conserved regions of the HIV-1 proteome, which were presented to the immune system using a heterologous prime-boost combination of plasmid DNA, a non-replicating simian (chimpanzee) adenovirus ChAdV-63 and a non-replicating poxvirus, modified vaccinia virus Ankara. A block-randomized, single-blind, placebo-controlled phase I trial HIV-CORE 002 administered for the first time candidate HIV-1- vaccines or placebo to 32 healthy HIV-1/2-uninfected adults in Oxford, UK and elicited high frequencies of HIV-1-specific T cells capable of inhibiting HIV-1 replication *in vitro*. Here, detail safety and tolerability of these vaccines are reported.

**Methods:**

Local and systemic reactogenicity data were collected using structured interviews and study-specific diary cards. Data on all other adverse events were collected using open questions. Serum neutralizing antibody titres to ChAdV-63 were determined before and after vaccination.

**Results:**

Two volunteers withdrew for vaccine-unrelated reasons. No vaccine-related serious adverse events or reactions occurred during 190 person-months of follow-up. Local and systemic events after vaccination occurred in 27/32 individuals and most were mild (severity grade 1) and predominantly transient (<48 hours). Myalgia and flu-like symptoms were more strongly associated with MVA than ChAdV63 or DNA vectors and more common in vaccine recipients than in placebo. There were no intercurrent HIV-1 infections during follow-up. 2/24 volunteers had low ChAdV-63-neutralizing titres at baseline and 7 increased their titres to over 200 with a median (range) of 633 (231-1533) post-vaccination, which is of no safety concern.

**Conclusions:**

These data demonstrate safety and good tolerability of the pSG2.HIVconsv DNA, ChAdV63.HIVconsv and MVA.HIVconsv vaccines and together with their high immunogenicity support their further development towards efficacy studies.

**Trial Registration:**

ClinicalTrials.gov NCT01151319

## Introduction

Vaccines are highly cost-effective tools for population health. While vaccines for over 40 human pathogens including human immunodeficiency virus type 1 (HIV-1) are urgently needed, safety of the volunteers remains the primary concern. The biggest roadblock in the development of an effective HIV-1 vaccine is the ability of HIV-1 to escape selective pressure of host immune response. To tackle this problem, we designed vaccine immunogen HIVconsv, derived from the functionally most conserved regions of the HIV-1 proteome [1]. These regions are common to most virus variants around the globe and the virus cannot easily change them without a likely decrease in its replicative fitness [2]–[5]. The gene coding for the HIVconsv immunogen was inserted into plasmid DNA (pSG2.HIVconsv: D), simian (chimpanzee) adenovirus (ChAdV63.HIVconsv; C), which is at least equally immunogenic to human adenovirus 5, but has very low pre-existing antibody responses, and modified vaccinia virus Ankara (MVA.HIVconsv; M) [1], [6], [7]. The pre-clinical safety and immunogenicity of these vaccines were demonstrated in mice and rhesus macaques[1], [7]–[9]. Two Good Laboratory Practice repeat-dose studies in the males and females of BALB/c mice showed no systemic toxicity of these three vaccine candidates [10] and supported approval by the Medicines and Healthcare Products Regulatory Agency of the UK for their use in a phase I clinical trial. The first phase I trial HIV-CORE 002 (for COnserved REgions) of the pSG2.HIVconsv, ChAdV63.HIVconsv and MVA.HIVconsv vaccines administered in C, CM, DDDCM and DDDMC regimens to healthy HIV-1/2-uninfected adults in Oxford, UK showed high induction of HIV-1-specific T cells. Furthermore, the CD8^+^ T-cell effectors were capable of inhibiting growth of multiple strains of HIV-1 in autologous CD4^+^ cells *in vitro* [11]. Here, we report that all the vaccines and regimens were safe and well tolerated by the trial volunteers.

## Methods

The protocol for this trial and the supporting CONSORT checklist are available as supporting information; see Checklist S1 and Protocol S1.

### Ethical and regulatory approvals

Ethical and regulatory approvals to conduct the HIV-CORE 002 trial, were obtained from the National Research Ethics Service Committee West London (Ref: 10/H0707/52) and the UK Medicines and Healthcare products Regulatory Agency (Ref: 21584/0271/001). The study was conducted according to the principles of the Declaration of Helsinki (2008) and complied with the guidelines of the International Conference on Harmonization Good Clinical Practice.

### The vaccines

The pSG2.HIVconsv DNA vaccine [1], [7] was produced by the Clinical Biotechnology Centre, Bristol Institute for Transfusion Science, University of Bristol, UK and formulated in phosphate buffered saline pH 7.4 at 4.0 mg/ml. The ChAdV63.HIVconsv vaccine [1], [7] was produced at the Clinical Biomanufacturing Facility, University of Oxford, UK and diluted in formulation buffer to 1.35×10^11^ virus particles (vp)/ml (36 vp/infectious unit). The MVA.HIVconsv vaccine [1], [7] was produced by IDT Biologika GmbH, Germany and diluted in a formulation buffer to 5.5×10^8^ plaque-forming units (PFU)/ml. All vaccines were stored below −70°C until use. Both the MVA.HIVconsv and ChAdV63.HIVconsv were genetically stable over 7 blind passages (not shown). All three vaccines expressed shared immunogen HIVconsv, which is a chimaeric protein assembled from the 14 most conserved sub-protein regions of the HIV-1 proteome using consensus sequences of alternating clades A, B, C and D [1], [11].

### Study objectives

The primary objectives of the HIV-CORE 002 trial were to evaluate the short-term safety of one low dose ChAdV63.HIVconsv and the standard doses of pSG2.HIVconsv DNA, ChAdV63.HIVconsv and MVA.HIVconsv vaccines administered sequentially in heterologous prime-boost regimens into healthy, low-risk, HIV-1-uninfected adult volunteers (Table 1). The secondary objectives were to evaluate and compare the T-cell induction among groups and further characterize the T-cell function and phenotypes, of which the main part has been already published [11].

**Table 1:** Visit and vaccination schedules [11].

Visit number	V:P	1	2	3	4	5	6	7	8	9	10	11
Week		Scr	0	1	2	4	8	16	28	-	-	-
Group 1	2:0	-	c	-	-	-	-	-	-	-	-	-
Week		Scr	0	1	2	4	8	9	12	20	28	-
Group 2	8:2	-	C/P	-	-	-	M/P	-	-	-	-	-
Week		Scr	0	4	8	12	13	14	20	21	22	28
Group 3	8:2	-	D/P	D/P	D/P	C/P	-	-	M/P	-	-	-
Week		Scr	0	4	8	12	13	16	17	18	24	28
Group 4	8:2	-	D/P	D/P	D/P	M/P	-	C/P	-	-	-	-

V:P – vaccine to placebo ratio; Scr – screen; c – low dose ChAdV63.HIVconsv 5×10^9^ vp; C – standard dose ChAdV63.HIVconsv 5×10^10^ vp; D – 4 mg pSG2.HIVconsv DNA; M – MVA.HIVconsv 2×10^8^ PFU; P – placebo (saline).

### Participants

**Figure 1:**
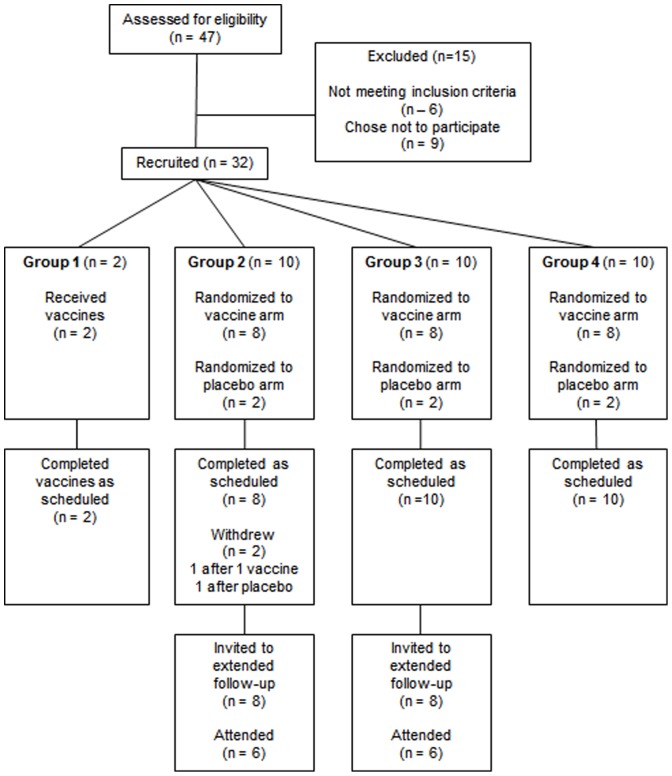
CONSORT flow diagram for HIV-CORE 002. Each vaccine and placebo dose was delivered by two injections, one into each arm.

The study was conducted at the Centre for Clinical Vaccinology and Tropical Medicine, University of Oxford, Oxford, UK. All volunteers in this trial were confirmed to be HIV-uninfected and at low risk of acquiring HIV-1 infection prior to enrolment. Healthy males and non-pregnant females aged 18–50 who fully comprehended the purpose, details and any possible consequences of this study and were able to provide and sign a written informed consent were invited to participate in the study (Fig. 1). Eligibility was assessed by review of medical histories and potential risk behaviours, physical examination, urinalysis and haematological, biochemical and serological tests. Volunteers were screened for hepatitis B and C virus infections and syphilis. Urine pregnancy tests were performed in all female volunteers. The inclusion and exclusion criteria are provided in the accompanying Study Protocol (Protocol S1). There was no selection of volunteers on the basis of pre-existing neutralizing antibodies to the ChAdV-63 or human adenovirus type 5 (HAdV-5) prior to enrolment.

### Study design

This was a phase I block-randomised, single-blind, placebo-controlled trial comprising four groups (Table 1), whereby the recipients were blinded. Sealed envelopes were used for the purpose of allocation concealment. Randomization for Groups 2–4 was generated by Centre for Statistics in Medicine, University of Oxford and implemented by sealed envelope allocations. In Group 1, two volunteers were scheduled to receive a single immunization with ChAdV63.HIVconsv at a low dose of 5×10^9^ vp as this was the first administration of this novel vector and insert combination in human subjects. Groups 2, 3 and 4 proceeded sequentially and each enrolled 10 subjects, of whom 8 were randomized to receive study vaccines and 2 to receive placebos (0.9% saline) (Table 1). All vaccines and placebos were delivered by intramuscular needle injection into the deltoid region of both arms (each dose was divided between two arms). This study entailed 28 weeks of clinical and laboratory follow-up after the day-0 vaccination.

### Sample size

Previous studies have shown that recombinant DNA and MVA vaccines are very well tolerated [12]–[18]. The ChAdV-63 and other replication-defective ChAdV vectors were also shown to be safe in over 1,000 healthy volunteers [19]–[22]. Nevertheless, the present study was a first-in-human evaluation of pSG2.HIVconsv DNA and ChAdV63.HIVconsv and as such, the study was primarily descriptive.

### Safety assessments

All assessments were carried out at specified time points according to the study protocol (Protocol S1). Evaluations of the vaccination site, vital signs (resting pulse and blood pressure, oral temperature), local and systemic reactogenicity were performed by the study staff on each vaccination day immediately prior to administration of the vaccine, at 30 minutes after vaccination, and at specified follow-up visits at the clinic. Data on local and systemic reactogenicity events were collected prospectively using structured interviews conducted by the trial nurse and/or physician and study-specific diary cards that were completed by the volunteers. Data on other events were collected with open questions. Local reactogenicity included assessment of skin reactions (erythema or skin discoloration, skin damage/vesiculation or ulceration, induration/formation of crust or scab), pain, swelling and itch. Systemic reactogenicity assessments included myalgia, flu-like symptoms, fatigue/malaise, oral temperature, chills, headache, nausea, allergic reactions and vomiting. Both local and systemic events were graded according to Grading Toxicity Tables given in the Clinical Protocol (Division of AIDS 2004, Protocol S1). Although this was a first-in-human evaluation of pSG2.HIVconsv and ChAdV63.HIVconsv, 3 rather than 7 days post-vaccination assessment was judged to be sufficient due to extensive safety data on plasmid DNA, MVA and simian adenovirus-derived vaccine vectors including ChAdV-63 [19]–[23]. AEs were specified as unrelated, unlikely, possibly, probably, related to the received treatment.

Laboratory parameters (haematology, biochemistry) were measured as specified in the protocol (Protocol S1).

### ChAdV-63 neutralizing antibody assay

ChAdV-63 nAb titers were assayed as previously described [24] using a secreted alkaline phosphatase (SEAP) assay. Briefly, 3.5×10^4^ HEK293 cells per well were seeded in a 96-well-plate for 2 days. SEAP-expressing ChAdV-63 was pre-incubated for 1 hour at 37°C alone or with serial dilutions (1∶18, 1∶72. 1∶288, 1∶1152 and 1∶4608) of heat-inactivated serum from trial volunteers, added to the 95–100% confluent HEK293 cells for 1 hour at 37°C, and the supernatant was then removed and replaced with 10% FCS in DMEM. SEAP activity in the supernatant was measured after 24 ± 2 hours using the chemiluminescent substrate (CSPD) from Phospha-Light kit (Tropix) following manufacturer's instructions. Light signal output expressed as relative light units (RLU) was measured 45 minutes after the addition of the CSPD substrate using a luminometer (Envision 2102 Multilabel reader, Perkin Elmer). The neutralization titer was defined as the reciprocal of sera dilution required to inhibits SEAP expression by 50% compared to the SEAP expression of virus infection alone.

## Results

### Study recruitment

Recruitment took place between March 2011 and February 2012. Thirty-two healthy HIV-1- and HIV-2-negative adult volunteers (15 females and 17 males), who signed an informed consent, were enrolled, immunized and followed up (Table 2). The median age of volunteers was 29 (range 18–50) years. One ChAdV63.HIVconsv and one placebo recipient in Group 2 withdrew after receiving their first allocated administration for reasons unconnected to trial. These individuals were followed up for 4 weeks. The remaining 30 volunteers attended almost all (>99%) visits as scheduled and completed the study. After unblinding, the protocol was extended to enable follow-up of vaccinees in Groups 2 and 3 up to 2 years after the last vaccination: 5/8 vaccinees from Group 2 and 7/8 vaccinees from Group 3 consented to additional follow-up. The study recruitment is summarized in Consort flow diagram (Fig. 1).

**Table 2:** HIV-CORE 002 volunteer demographics.

Gender	
Male	15
Female	17
Age median (range) years	29 (18–50)
Vaccine received	
Low dose c	2
Standard dose C	24
M	23
D (number of dosings)	16 (48)
Extended follow up	
1 year	12

c, C – ChAdV63.HIVconsv; M – MVA.HIVconsv; D – pSG2.HIVconsv DNA.

### Adverse events

No serious adverse reactions or suspected unexpected adverse reactions occurred during the study. One volunteer experienced a single SAE (prior to receipt of any vaccines (appendicitis requiring surgery). Eighty adverse events were recorded during the trial, all of which were mild or moderate (grade 1–2). The majority were judged unlikely to be related to the study vaccines. In particular, the placebo arm recorded 1 unrelated and 4 unlikely, and the vaccine arm registered 16 unrelated, 56 unlikely and 3 possibly related adverse events to the received treatment.

**Figure 2:**
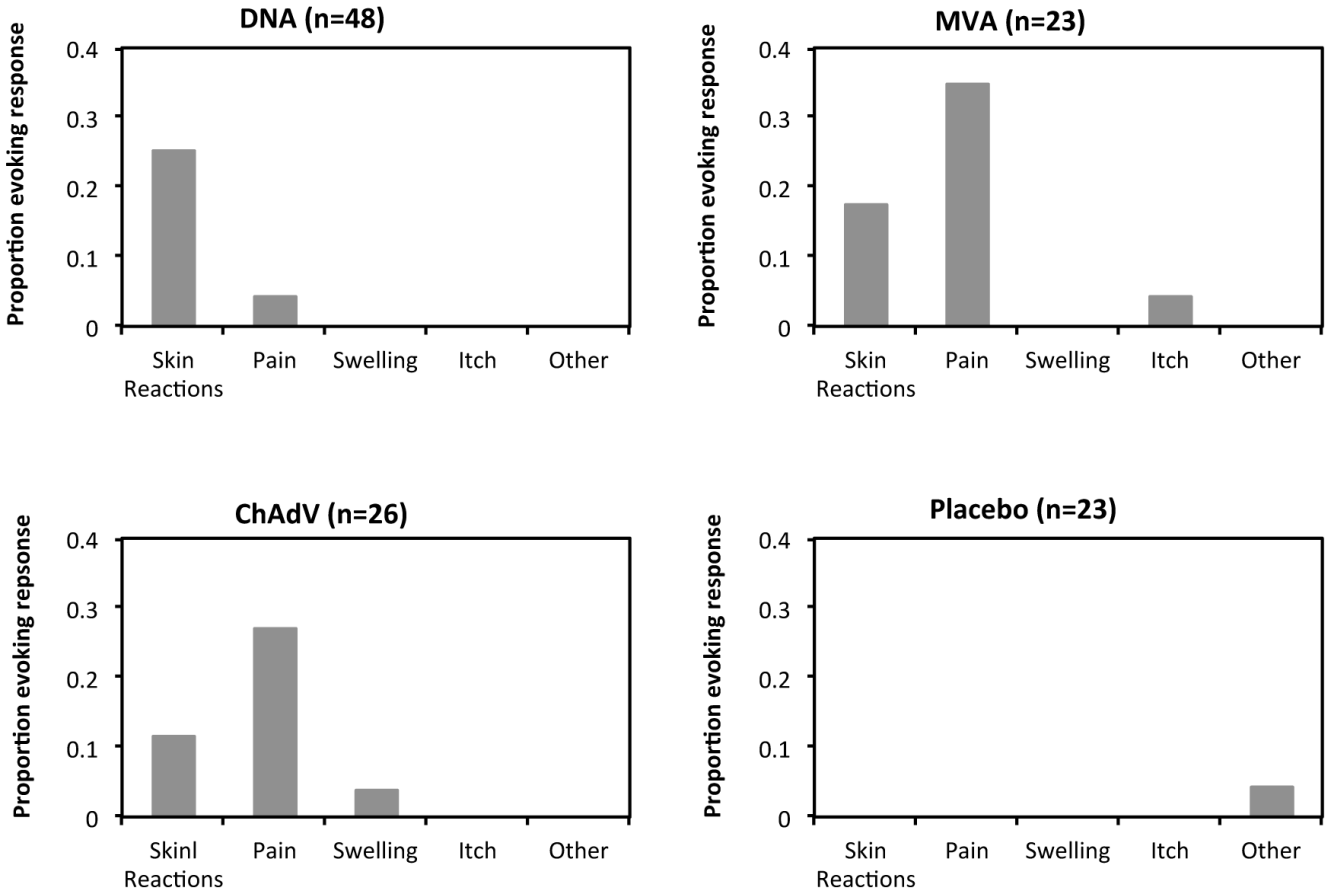
Summary of local reactions. Volunteers received vaccines pSG2.HIVconsv DNA (D), ChAdV63.HIVcocnsv (c – low dose and C – standard dose) and MVA.HIVconsv (M) in 4 groups using regimens c, (n  =  2), CM, DDDCM and DDDMC (n  =  10) and in Groups 2–4, were randomized between 8 and 2 recipients of vaccine and placebo, respectively. Local reactions were recorded by the volunteers themselves and assessed and scored by the clinical team. ‘Other’ - one placebo recipient reported a ‘slightly stiff left shoulder’.

Local reactogenicity is summarized in Fig. 2 and note that all vaccine and placebo doses were injected into two sites, one on each arm. Thus, 16/16 volunteers received all 3 scheduled doses of pSG2.HIVconsv DNA and the most common finding was mild (grade 1 severity) erythema at the site of injection, which resolved when the arms were examined at 7 days. Of the 23 volunteers who received the MVA.HIVconsv vaccine dose, 8 reported mild (grade 1), pain at the injection site, which persisted to 7 days in 2 cases. Local erythema was less common and was again mild when it occurred. The ChAdV63.HIVconsv vaccine was administered at low and high doses to 2 and 24 volunteers, respectively, of whom 7 reported mild pain, which lasted 2 weeks for 1 individual and less than 3 days for the others. Following 23 administrations of placebo, one volunteer reported a ‘slightly stiff shoulder’ the day following vaccination.

**Figure 3:**
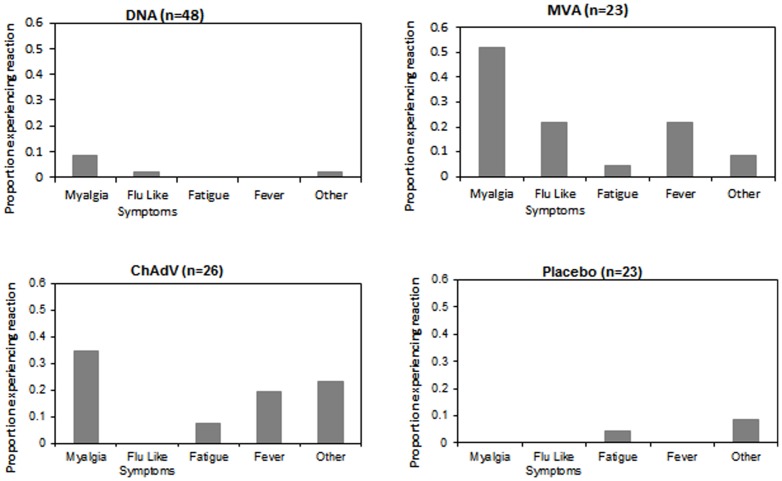
Summary of systemic reactions. Following administration of the pSG2.HIVconsv DNA (D), ChAdV63.HIVconsv (c – low dose and C – standard dose) and MVA.HIVconsv (M) vaccines (Groups 1–4) or placebo (Groups 2, 3 and 4), the volunteers themselves and the clinical team recorded and scored systemic reactions. ‘Other’ encompasses allergic reaction, chills/rigors, headache, vomit, stomach cramps and syncope.

Systemic reactions are summarized in Fig. 3. The pSG2.HIVconsv DNA recipients reported a few systemic effects. Myalgia was the most common, reported after 2 vaccine injections in 1 volunteer, and after a single vaccine in 2 others. MVA.HIVconsv: 12 volunteers reported myalgia. This was severe enough to warrant a day off work for 1 volunteer (i.e., grade 2). Maximum reported duration was 2 days. Fever (>37.7) was reported in 5, all of whom also described other symptoms- myalgia, ‘flu like symptoms, or nausea.

ChAdV63.HIVconsv: 16 volunteers reported systemic effects, 6 of these reporting multiple symptoms. 9 volunteers reported mild myalgia post vaccination, lasting up to 3 days. 5 reported fever and 5 headache. Of placebo recipients, 1 reported abdominal pain, 1 vomiting and 1 fatigue post vaccination.

Local and systemic reactions and their severity in relation to vaccine type/placebo are detailed in Table S1.

### Laboratory abnormalities

There were 33 abnormalities on haematological and biochemical tests. These were mostly isolated single values falling just outside the normal range for potassium, bilirubin, ALT, and haemoglobin that did not fulfil criteria for a grade 1 event (not shown). Volunteers were monitored for antibody responses to HIV-1 at the beginning and end of the study. One volunteer had a false positive result at the screening visit (4^th^ generation assay), but further testing indicated that this individual was HIV-1-seronegative. There were no positive reactions on HIV-1 serological testing after vaccination in any volunteer throughout the study.

### Anti-ChAdV-63 neutralizing antibody antibodies

**Figure 4:**
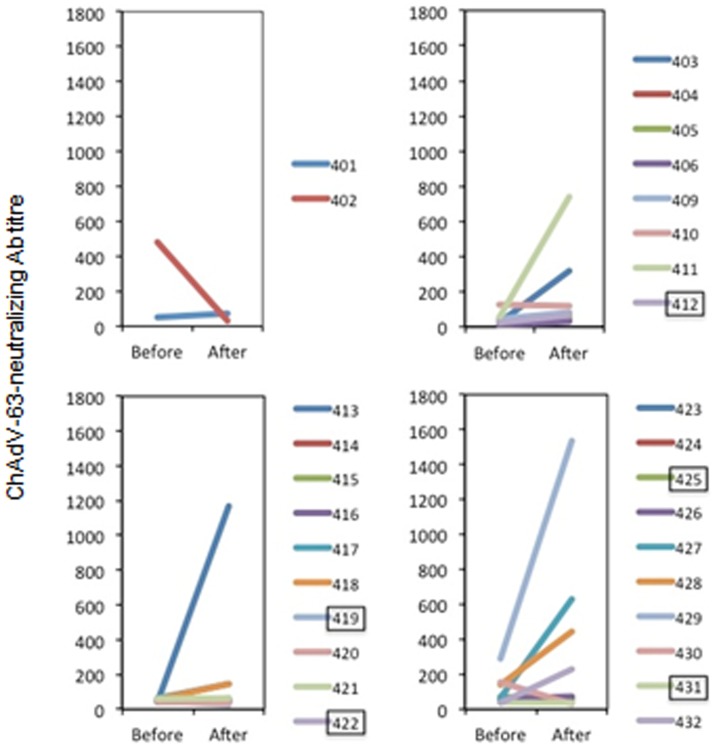
Vaccine-elicited neutralizing antibodies to ChAdV-63. Volunteers were given ChAdV63.HIVconsv alone or as a part of a heterologous prime-boost regimen and their sera were tested before and 2 weeks after the ChAdV63.HIVconsv administration for ChAdV-63 neutralizing antibodies. Boxed volunteer numbers indicate placebo recipients.

The pre- and post-ChAdV63.HIVconsv vaccination levels of antibodies neutralizing the ChAdV-63 vector were determined. An adenovirus neutralizing antibody titre of 200 was used as a cut-off here as it had been used previously to stratify analyses in studies of adenovirus type 5-vectored vaccines [25], [26]. Overall, the vast majority of volunteers had pre-vaccination baseline titres against ChAdV-63 below 200 and only 2/30 (6.7%) subjects had titres between 200 and 500. Following ChAdV63.HIVconsv vaccination, 7 volunteers developed ChAdV-63 neutralizing antibody >200, with median (range) of 633 (231 and 1533); the highest titre (volunteer no. 429) was indeed detected in one of the subjects with pre-existing Abs (Fig. 4).

## Discussion

This phase I study of the experimental HIV-1 vaccines pSG2.HIVconsv DNA, ChAdV63.HIVconsv and MVA.HIVconsv in healthy, volunteers at low risk of HIV infection showed that the vaccines were safe and well tolerated. There were no vaccine-related SAEs or serious adverse reactions over the study follow-up. Local and systemic events after vaccination were mild and transient, and the systemic reactogenicity was more associated with the MVA and ChAdV-63 vectors than DNA and with more vaccines than with placebo. No volunteer acquired HIV-1 infection during the study and the HIVconsv immunogen did not induce antibodies that could cause a reaction on standard HIV-1 diagnostic tests in any vaccine recipient.

An effective HIV-1 vaccine could be used both in prophylactic and therapeutic settings [27]. The former would be administered to uninfected populations to prevent infection or control HIV-1 replication post-infection and thus postpone development of AIDS perhaps indefinitely. The latter would be given to HIV-1-infected patients, in whom T cell-eliciting vaccines aim to either decrease virus load or together with antiretroviral treatment and reactivation of latent HIV-1 aim to eliminate the virus from the body completely. The safety standards for prophylactic and therapeutic vaccine applications are expected to be similar given the near-normal life expectancy that is achievable with combination antiretroviral therapy, therefore, in the case of the vaccines employed in trial HIV-CORE 002, the safety data so far support further development of the vaccination regimens for both prevention and therapy of HIV-1/AIDS.

The HIV-CORE 002 trial tested combinations of 3 vaccine modalities. In general, the safety of subunit vaccines vectored by DNA and MVA has been demonstrated in numerous trials reviewed elsewhere [13], [15], [28]–[33]. In contrast, the safety of recombinant human adenovirus serotype-5 (HAdV-5) vaccines was questioned in a recent meta-analysis of phase IIb trials [34]. Post-hoc analysis of the Step and Phambili trials raised concerns regarding potential enhancement of HIV-1 acquisition risk after vaccination with the MRKAd5 vaccine [25], [35]-[38]. However, these findings arose from analyses that were not pre-specified, did not reach statistical significance and/or included data that were collected from 80% after study unblinding. Importantly, HVTN 505 specifically addressed a concern raised in HVTN 502 regarding the role of pre-existing Ad5-specific antibodies by excluding individuals with these responses; this trial did not show a significant increase in HIV-1 acquisition in vaccinees [39]. The vector ChAdV-63 employed in HIV-CORE 002 was selected because of its high immunogenicity and yet a number of significant differences from HAdV-5 [6]. Thus, as confirmed by our analysis, pre-existing nAb were rare and of low titres. These results agree with a previous study, in which less than 3% of 193 tested Caucasian human healthy volunteers from diverse regions of Europe and the US had nAb titers over 200 [6]. Also of the 200 individual sera taken from a cohort of 1- to 6-year-old children from Kenya, only 4% contained high-titer neutralizing antibodies to ChAdV-63 [40]. Biologically, HAdV-5 is a group C adenovirus, while ChAdV-63 belongs to group E adenoviruses. The only HAdV representative of group E is HAdV-4 used in over 10 million military recruits in the US as a live, enteric-coated oral vaccine, and proven safe and effective again homologous HAdV infections. Yet, antibodies induced by HAdV-4 vaccine display minimal cross-reactivity with non-human adenoviruses [26]. Due to the difference in sequences of the capsid proteins, ChAdV-63 and HAdV-5 showed little serum cross-reactivity [6], [20], [40]. In contrast to antibodies, highly conserved hexon-specific T cells cross-reactive against most AdVs species are common [41], yet, with exception of one study of HAdV-5 [42], they do not seem to affect immunogenicity for the passenger transgenes [11], [19]–[22]. Nevertheless, assessment of their role, trafficking and fate is now mandated. Finally, non-replicating recombinant ChAdV-63 vaccines have been safe when administered to date to more than 1,000 subjects in the North and South [19]–[22] including children and infants in Africa, and HIV-1-infected patients (unpublished) with overall a single dose reactogenicity comparable to HAdV-35-vectored vaccine Ad35-GRIN [43]. Immunogens delivered by ChAdV-63 were highly immunogenic for both T-cell and antibody responses [11], [19]-[22]. Thus, our data support the development of ChAdVs as vectors for preventive HIV-1 vaccines, with careful monitoring for any unanticipated, statistically significant indications of suspect side effects.

The combined vaccine regimens of CM and DDDCM induced uniquely high frequencies of CD8^+^ T cells specific for the conserved regions of HIV-1 common to most HIV-1 variants, which were capable of inhibiting HIV-1 replication in autologous CD4^+^ cells [11]. The immunogenicity and *in vitro* efficacy data together with an excellent safety of the vectors reported here strongly support further development of this anti-HIV-1 vaccine strategy [27] alone and/or in a combination with antibody-induced vaccine components.

## Supporting Information

Checklist S1 **CONSORT 2010 checklist of information on the clinical trial HIV-CORE 002 as presented in this manuscript.**
(PDF)

Table S1 **Severity of local and systemic adverse reactions in HIV-CORE 002.**
(PDF)

Protocol S1 **HIV-CORE 002 Clinical Trial Protocol.**
(PDF)
