# Supplementary material for: Safety and Tolerability of Conserved Region Vaccines Vectored by Plasmid DNA, Simian Adenovirus and Modified Vaccinia Virus Ankara Administered to Human Immunodeficiency Virus Type 1-Uninfected Adults in a Randomized, Single-Blind Phase I Trial
Source: PLoS One. 2014 Jul 9;9(7):e101591. doi: 10.1371/journal.pone.0101591 (PMC4090156; doi:10.1371/journal.pone.0101591)
Supplement: Table S1 — Severity of local and systemic adverse reactions in HIV-CORE 002. (PDF) [file pone.0101591.s002.pdf]

Table S1. Severity of local and systemic adverse reactions in HIV-CORE 002.

**Local AR**	Severity					Severity					Severity					Severity			
**pSG2.HIVconsv DNA**	**1**	**2**	**3**	**4**	**MVA.HIVconsv**	**1**	**2**	**3**	**4**	**ChAdV63.HIVconsv**	**1**	**2**	**3**	**4**	**Placebo**	**1**	**2**	**3**	**4**
Pain/tenderness	6	0	0	0	Pain/tenderness	13	0	0	0	Pain/tenderness	8	0	0	0	Pain/tenderness	1	0	0	0
Pruritis	0	0	0	0	Pruritis	1	0	0	0	Pruritis	0	0	0	0	Pruritis	0	0	0	0
Skin reaction	12	0	0	0	Skin reaction	5	0	0	0	Skin reaction	7	0	0	0	Skin reaction	0	0	0	0

**Systemic AR**	Severity					Severity					Severity					Severity			
**pSG2.HIVconsv DNA**	**1**	**2**	**3**	**4**	**MVA.HIVconsv**	**1**	**2**	**3**	**4**	**ChAdV63.HIVconsv**	**1**	**2**	**3**	**4**	**Placebo**	**1**	**2**	**3**	**4**
allergic reaction	0	0	1	0	allergic reaction	0	0	0	0	allergic reaction	0	0	0	0	allergic reaction	0	0	0	0
chills/rigors	1	0	0	0	chills/rigors	4	0	0	0	chills/rigors	2	0	0	0	chills/rigors	0	0	0	0
fatigue/malaise	0	0	0	0	fatigue/malaise	2	0	0	0	fatigue/malaise	3	0	0	0	fatigue/malaise	1	0	0	0
pain/myalgia	1	0	0	0	pain/myalgia	7	0	0	0	pain/myalgia	7	0	0	0	pain/myalgia	0	0	0	0
headache	0	0	0	0	headache	0	0	0	0	headache	5	0	0	0	headache	0	0	0	0
fever	0	0	0	0	fever	9	0	0	0	fever	4	0	0	0	fever	0	0	0	0
					syncope	1	0	0	0	cough	1	0	0	0	vomit	0	1	0	0
					sore throat	1	0	0	0						stomach cramps	1	0	0	0

There were the following numbers of doses administered of each modality: pSG2.HIVconsv DNA 48, MVA.HIVconsv 23, ChAdV63.HIVconsv 2 low doses and 24 high doses, and placebo 23. Each dose was injected into two sites, one on each arm.
