# Supplementary material for: Safety and Tolerability of Conserved Region Vaccines Vectored by Plasmid DNA, Simian Adenovirus and Modified Vaccinia Virus Ankara Administered to Human Immunodeficiency Virus Type 1-Uninfected Adults in a Randomized, Single-Blind Phase I Trial
Source: PLoS One. 2014 Jul 9;9(7):e101591. doi: 10.1371/journal.pone.0101591 (PMC4090156; doi:10.1371/journal.pone.0101591)
Supplement: Protocol S1 — HIV-CORE 002 Clinical Trial Protocol. (PDF) [file pone.0101591.s003.pdf]

Study Title: A randomized single-blind placebo-controlled study to evaluate the safety and immunogenicity of three candidate HIV-1 vaccines, pSG2.HIVconsv DNA, ChAdV63.HIVconsv and MVA.HIVconsv, administered in combination to healthy HIV-1 uninfected adults.

Internal Reference No: HIV-CORE 002
Ethics Ref:
Eudract Number: 2010-018439-16
Version No: 1.8
Date: 14th January, 2011
Funder: Medical Research Council

This document contains confidential information that must not be disclosed to anyone other than the Sponsor, the Investigator Team, host NHS Trust (s), regulatory authorities, and members of the Research Ethics Committee.

Contact information

Chief Investigator (correspondence)<br>Dr Tomáš Hanke<br>The Jenner Institute<br>Old Road Campus Reaserch Building<br>Roosevelt Drive<br>Oxford OX3 7DQ	Telephone:<br>Fax:<br>Mobile:<br>E-mail:	+44 (0)1865 617630<br>+44 (0)1865 617608<br>+44 (0)7773427147<br>tomas.hanke@imm.ox.ac.uk
Principal Investigator<br>Dr Lucy Dorrell<br>MRC Human Immunology Unit,<br>Weatherall Institute of Molecular Medicine<br>John Radcliffe Hospital, Headley Way<br>Oxford, OX3 9DS, UK	Telephone:<br>Fax:<br>Mobile:<br>E-mail:	+44 (0)1865 222145<br>+44 (0)1865 222502<br><br>lucy.dorrell@imm.ox.ac.uk
Trial Sponsor<br>University of Oxford		
Trial Monitoring<br>Clinical Trial Research Governance Manor House, John Radcliffe Hospital, Headington, Oxford OX3 9DZ	Telephone:<br>Fax:	01865 857939<br>01865 743002
Investigational Site<br>Centre for Clinical Vaccinology and Tropical Medicine<br>Churchill Hospital, Headington, Oxford, OX3 7LJ, UK	Telephone:<br>Fax:<br>E-mail:	

SIGNATURE PAGE

The signatures below constitute the approval of this protocol and the appendices and provide the necessary assurances that this study will be conducted in compliance with the protocol, ICH-GCP and the applicable regulatory requirement (s).

Chief Investigator: Tomáš Hanke

Date:

Signed: ______________________________ ____________

Principal Investigator: Lucy Dorrell

Date:

Signed: ______________________________ ____________

Table of Contents

1 SYNOPSIS 8

2 ABBREVIATIONS 9

3 BACKGROUND information 11

3.1 Importance and background 11

3.2 T cell responses and protection against HIV-1/AIDS 11

3.3 Dealing with HIV-1 diversity 11

3.4 Design of the study immunogen HIVconsv 12

3.5 Vaccine vectors and regimens 13

3.6 The investigational pSG2.HIVconsv DNA vaccine 14

3.6.1 Plasmid DNA as a vaccine vector 14

3.6.2 Clinical experience with DNA vaccines 14

3.6.3 Clinical experience with pSG2 plasmid DNA-vectored vaccines 14

3.6.4 pSG2.HIVconsv dose and dosing schedule justification 14

3.7 The investigational MVA.HIVconsv vaccine 15

3.7.1 Modified vaccinia virus Ankara 15

3.7.2 Pre-clinical studies with MVA-vectored vaccines 15

3.7.3 Clinical experience with MVA-vectored vaccines 15

3.7.4 Clinical experience with MVA.HIVconsv 16

3.7.5 MVA.HIVconsv dose justification 16

3.8 The investigational ChAdV63.HIVconsv vaccine 17

3.8.1 Attenuated adenoviruses as vaccine vectors 17

3.8.2 Pre-clinical studies with chimpanzee adenovirus-vectored HIVconsv vaccines 17

3.8.3 Clinical trials with chimpanzee adenovirus-vectored vaccines 17

3.8.4 -ChAdV63.HIVconsv dose justification 18

3.9 Pre-clinical studies of HIVconsv vaccines in heterologous regimens 18

3.10 Pre-clinical GLP toxicology using HIVconsv vaccines 18

4 STUDY OBJECTIVES 19

4.1 Primary Objectives 19

4.2 Secondary Objectives 19

5 STUDY DESIGN 19

5.1 Summary of study design 19

5.2 Study Duration 20

5.3 Primary and secondary endpoints/outcome measures 20

5.3.1 Primary Endpoints 20
5.3.2 Secondary Endpoints 20
5.4 Study Population 21
5.4.1 Overall description of trial participants 21
5.4.2 Inclusion Criteria 21
5.4.3 Exclusion Criteria 21
5.5 Recruitment 23
5.5.1 Reimbursement 23
5.6 Study Procedures 23
5.6.1 Study visits 23
5.6.2 Vaccination visit 24
5.6.3 Follow-Up Visits (including Final or Early Termination visit) 24
5.6.4 Final Visit or Early Termination Visit 25
5.6.5 Unscheduled visits 25
5.6.6 Informed Consent 25
5.6.7 Counseling regarding contraception & use of condoms 25
5.6.8 Medical history and physical examination 25
5.6.9 Blood and urine collection 26
5.6.10 Laboratory results 26
5.7 Randomization 26
5.8 Unblinding procedures 26
5.9 Definition of End of Study 26
6 INVESTIGATIONAL MEDICINAL PRODUCT 26
6.1 Description 26
6.2 Packaging, Storage and Shipment 27
6.3 Dispensing and Handling 28
6.4 Administration 28
6.5 Accountability and Disposal 28
7 ASSESSMENTS 28
7.1 Safety Assessments 28
7.1.1 Local Reactogenicity Events 28
7.1.2 Systemic Reactogenicity Events 29
7.1.3 Other Adverse Events 29
7.1.4 Routine Laboratory Parameters 29
7.1.5 Specific Screening Tests 29
7.2 Immunogenicity Assessments 30

7.2.1 Cellular Responses 30
7.3 Other Assessments 30
7.3.1 HLA Typing 30
7.3.2 HIV Antibody Test 30
7.3.3 Pregnancy Test 30
8 SAFETY REPORTING 30
8.1 Definitions 30
8.1.1 Adverse Event (AE) 30
8.1.2 Adverse Reaction (AR) 30
8.1.3 Serious Adverse Events and Reactions 31
8.1.4 Suspected Unexpected Serious Adverse Reaction 31
8.1.5 Severe Adverse Events 31
8.2 Severity Grading of Adverse Events 31
8.3 Relationship to Investigational Medicinal Product 32
8.4 Procedures for Recording Adverse Events 33
8.4.1 Reporting Serious Adverse Events 33
8.4.2 Reporting Other Events 33
8.5 Clinical Management 34
9 MANAGEMENT OF HIV-1 ISSUES DURING AND FOLLOWING THE STUDY 34
9.1 HIV-1 Testing 34
9.2 Social Discrimination as a Result of Antibody Response to IMP 34
10 DISCONTINUATION OR POSTPONEMENT OF VACCINATIONs AND/OR WITHDRAWal FROM THE STUDY 34
10.1 Discontinuation or Postponement of Vaccinations 34
10.1.1 Follow-Up after Discontinuation of Vaccinations 35
10.2 Early Termination 36
11 DATA HANDLING 36
11.1 Data Collection and Record Keeping at the Trial Site 36
11.2 Data Entry at the Trial Site 36
11.3 Data Analysis 36
12 STATISTICAL CONSIDERATIONS 36
12.1 Statistical Power and Analysis 36
12.1.1 Safety and Tolerability 37
12.1.2 Immunogenicity 37

13 QUALITY CONTROL AND QUALITY ASSURANCE....37

14 DATA AND BIOLOGICAL MATERIAL....37

15 ADMINiSTRATIVE STRUCTURE....38

15.1 Data Monitoring Committee (DMC)....38

15.2 Content of Interim Review....38

15.3 Indications for Discontinuation of Vaccinations in all Volunteers....39

16 INDEMNITY....39

17 PUBLICATION....39

18 REGULATORY, ETHICAL AND LEGAL OBLIGATIONS....40

18.1 Declaration of Helsinki....40

18.2 ICH Guidelines for Good Clinical Practice....40

18.3 Participant Confidentiality....40

19 DATA PROTECTION....40

20 REFERENCES....41

APPENDIX A - SCHEDULE OF PROCEDURES....54

APPENDIX B - Volunteer INVITATION LETTER version 2.0....58

APPENDIX C - PARTICIPANT INFORMATION LEAFLET version 2.0....59

APPENDIX D – CONSENT FORM version 1.0....66

APPENDIX E - PARTICIPANT DIARY CARD....67

APPENDIX F - SAMPLE GP LETTER version 1.0....68

APPENDIX G - ADVERSE EVENT SEVERITY ASSESSMENT TABLE....69

1 SYNOPSIS

<table>
<tr><td>Study title</td><td>A randomized single-blind placebo-controlled study to evaluate the safety and immunogenicity of three candidate HIV-1 vaccines, pSG2.HIVconsv DNA, ChAdV63.HIVconsv and MVA.HIVconsv, administered in combination to healthy HIV-1 uninfected adults.</td></tr>
<tr><td>Clinical Phase</td><td>I/IIa</td></tr>
<tr><td>Trial Design</td><td>Randomized single-blind study</td></tr>
<tr><td>Study Population</td><td>HIV-1/2 negative, low-risk males and females 18-50 years of age.</td></tr>
<tr><td>Planned Sample Size</td><td>32</td></tr>
<tr><td>Planned Trial Period</td><td>16 months (from screening of the first volunteer to 6 months after final vaccination of the last volunteer)</td></tr>
<tr><td>Primary Objective</td><td>To evaluate the safety of candidate HIV-1 vaccines pSG2.HIVconsv DNA, ChAdV63.HIVconsv and MVA.HIVconsv administered intramuscularly as part of heterologous prime-boost regimens.</td></tr>
<tr><td>Secondary Objective</td><td>To evaluate the magnitude, specificity and quality of HIV-1-specific T cell responses after administration of pSG2.HIVconsv DNA, ChAdV63.HIVconsv and MVA.HIVconsv vaccines in heterologous prime-boost regimens.</td></tr>
<tr><td>Primary Endpoints</td><td>Proportion of volunteers who develop a grade 3 or 4 local reaction.
Proportion of volunteers who develop a grade 3 or 4 systemic reaction.</td></tr>
<tr><td>Secondary endpoints</td><td>Proportion of volunteers who develop new $CD8^+$ and $CD4^+$ T cell responses to one or more HIV-1 epitopes, as determined by IFN-γ ELISPOT assay.
Exploration of the efficacy of vaccine-induced $CD8^+$ T cells to suppress HIV-1 replication in vitro.</td></tr>
<tr><td>Investigational Medicinal Products</td><td>pSG2.HIVconsv DNA; attenuated chimp adenovirus ChAdV63.HIVconsv; attenuated poxvirus MVA.HIVconsv; or placebo.</td></tr>
<tr><td>Dose</td><td>ChAdV63.HIVconsv 5x10^9 vp (c) or 5x10^{10} vp (C)
MVA.HIVconsv 2x10^8 pfu (M)
pSG2.HIVconsv DNA 4 mg (D)</td></tr>
<tr><td>Route</td><td>Intramuscular needle injection</td></tr>
<tr><td>Study Design</td><td>
<table>
<tr><th>Stage</th><th>No.</th><th>Wk 0</th><th>Wk 4</th><th>Wk 8</th><th>Wk 12</th><th>Wk 16</th><th>Wk 20</th></tr>
<tr><td>1</td><td>2</td><td>c</td><td>-</td><td>-</td><td>-</td><td>-</td><td>-</td></tr>
<tr><td rowspan="2">2</td><td>8</td><td>C</td><td>-</td><td>M</td><td>-</td><td>-</td><td>-</td></tr>
<tr><td>2</td><td>P</td><td>-</td><td>P</td><td>-</td><td>-</td><td>-</td></tr>
<tr><td rowspan="2">3</td><td>8</td><td>D</td><td>D</td><td>D</td><td>C</td><td>-</td><td>M</td></tr>
<tr><td>2</td><td>P</td><td>P</td><td>P</td><td>P</td><td>-</td><td>P</td></tr>
<tr><td rowspan="2">4</td><td>8</td><td>D</td><td>D</td><td>D</td><td>M</td><td>C</td><td>-</td></tr>
<tr><td>2</td><td>P</td><td>P</td><td>P</td><td>P</td><td>P</td><td>-</td></tr>
</table>
</td></tr>
<tr><td>Follow-Up Duration</td><td>All individuals will be followed until 28 weeks after the initial vaccination.
Stage 1; Screen at -4 wks to 28 wks after final vaccination
Stage 2; Screen at -4 wks to 20 wks after final vaccination
Stage 3; Screen at -4 wks to 8 wks after final vaccination
Stage 4; Screen at -4 wks to 12 wks after final vaccination</td></tr>
<tr><td>Trial Sponsor</td><td>University of Oxford</td></tr>
</table>

2 ABBREVIATIONS

aa	Amino acid
HAdV-5	Human adenovirus serotype 5
AIDS	Acquired Immunodeficiency Syndrome
AE	Adverse Event
ALC	Absolute Lymphocyte Count
ALT	Alanine aminotransferase
ANC	Absolute Neutrophil Count
AR	Adverse reaction
AST	Aspartate aminotransferase
BCG	*Mycocterium bovis* bacillus Calmette–Guérin
CBF	Clinical Biomanufacturing Facility
ChAdV-63	Chimpanzee Adenovirus serotype 63
CI	Chief Investigator
CRF	Case Report Form
CSM	Centre for Statistics in Medicine
CTA	Clinical Trial Authorisation
CTL	Cytotoxic T Lymphocyte
DMC	Data Monitoring Committee
DNA	Deoxyribonucleic acid
EC	Ethics Committee (see REC)
ELISA	Enzyme-linked immunosorbent assay
ELISPOT	Enzyme-linked immunospot
GCP	Good Clinical Practice
GGT	Gamma-glutamyl transferase
GLP	Good Laboratory Practice
GMP	Good Manufacturing Practise
GP	General Practitioner
GTAC	Gene Therapy Advisory Committee
GUM	Genito-Urinary Medicine
HAART	Highly active antiretroviral treatment
HBsAg	Hepatitis B virus surface antigen
HCV	Hepatitis C virus
βhCG	Human Chorionic Gonadotrophin
HIV-1	Human Immunodeficiency Virus type I
HLA	Human Leukocyte Antigen
IB	Investigators Brochure
ICF	Informed Consent Form
ICH	International Conference on Harmonisation
IEC	Independent Ethics Committee
IFN-γ	Interferon gamma
IMP	Investigational Medicinal Product
IRB	Independent Review Board
IUD	Intra-uterine device
mAb	Monoclonal Antibody
mg	Milligram
μg	Microgram
MHC	Major Histocompatability Complex
MHRA	Medicines and Healthcare products Regulatory Agency
ml	Millilitre

mM	Millimolar
MRC	Medical Research Council
MVA	Modified Vaccinia Ankara
NAb	Neutralising Antibody
NHS	National Health Service
NRES	National Research Ethics Service (previously known as COREC)
PBS	Phosphate buffered saline
PCR	Polymerase Chain Reaction
pfu	Plaque-Forming Units
PI	Principal Investigator
PIL	Participant/ Patient Information Leaflet
R&D	NHS Trust Research & Development Department
RCA	Recombinant-competent adenovirus
SAE	Serious Adverse Event
SAR	Serious Adverse Reaction
SCID	Severe combined immunodeficiency
SIV	Simian Immunodeficiency Virus
SOP	Standard Operating Procedure
SUSAR	Suspected Unexpected Serious Adverse Reactions
ULN	Upper Limit of Normal
vp	Virus Particles
WBC	White Blood Cell Count

3 BACKGROUND INFORMATION

3.1 Importance and background

Development of an effective, accessible vaccine is the only realistic hope for halting the human immunodeficiency virus type 1 (HIV-1)/AIDS epidemic. Ideally, such a vaccine should induce broadly neutralizing antibodies and effective T cells at the same time; however, both of these goals face substantial and very different challenges *(1)*. A rational scientific strategy tackles and solves these roadblocks separately *(2)* before combining the two successful solutions into a single vaccine formulation.

3.2 T cell responses and protection against HIV-1/AIDS

We focus on the induction of protective T cell responses. To maximize the likelihood of early HIV-1 containment, vaccine-elicited T cells have to act quickly and efficiently *(3)*. To achieve this, such T cells must be present in sufficient numbers, recognize biologically constrained epitopes, exert protective functions and be present at the right place at the right time. Indeed, successful vaccine-induced T cell-mediated protection will likely require that all of these parameters are optimized simultaneously. First, there has to be a minimum number of HIV-1-specific T cells required for protection. Attempts to determine such numerical correlates have not been straightforward*(4-8)*, although analysis of T cell frequencies according to specificity has yielded some initial encouraging associations for Gag-derived epitopes*(9-11)*. Second, it will likely be advantageous for T cells to recognize the transmitted/founder virus *(12-14)* and specific epitopes that are presented on infected cells early rather than late after virus entry*(5, 12, 15-18)*. In addition, T cell responses that target multiple epitopes *(9, 11, 19)* in conserved regions of the HIV-1 genome*(20, 21)* will tend to hinder viral escape. Third, despite a substantial body of evidence that supports an important role for $CD8^+$ T cells in the control of HIV-1 replication*(19, 22-32)*, the definition of a single unifying protective determinant remains elusive*(33-35)*. The best correlates of virus control within a T cell response are high levels of antigen avidity and functional sensitivity *(36-41)*, rapid proliferation after exposure to cognate antigen*(36, 42)*, efficient killing of infected cells*(36, 42, 43)*, production of multiple soluble antiviral factors*(36, 43)* and, in certain circumstances, public clonotype mobilization from the available cognate repertoire *(44)*. Furthermore, to exert their effector functions efficiently, $CD8^+$ T cells need to receive optimal costimulatory signals *(45-47)*. Fourth, it is clear that functional T cells need to be present at the site of viral inoculation to protect against infection*(48)*. In addition, the parallel induction of anti-Env antibodies may attenuate the initial infection and provide time for T cells to expand optimally*(49, 50)*. Thus, the big challenge on the road to a successful HIV-1 vaccine is to find a means of stimulating as many of the potentially protective mechanisms as possible by active immunization.

3.3 Dealing with HIV-1 diversity

There are several approaches for dealing with the HIV-1 diversity. One optimistic view is that a single clade may induce sufficiently cross-reactive T-cell responses to protect against other variants of both the same and heterologous clades. The choice of a natural isolate can be based on having the closest sequence to all others, or picking a strain derived from acute infection and arguing that there is a convergence of viral sequences during transmission*(51)*. However, even if a single variant elicits responses that confer some cross-reactive protection, such protection is likely to be only partial and thus it is well worth attempting to design vaccine immunogens with enhanced cross-reactive potential. Although there are numerous reports of cross-clade reactive HIV-1-specific $CD8^+$ T cell responses *(52-56)*, use of

unphysiologically high concentrations of variant peptides make the biological relevance of many of these results uncertain. In contrast, there are ample examples of highly specific T cell receptors sensitive to single amino acid (aa) changes *(57-63)*, as well as compelling evidence of HIV-1 variants escaping existing T cell responses in infected individuals by single mutations in epitopes*(27, 64-66)*. In vitro, systematic studies employing all possible single aa substitutions in each position of a major histocompatibility complex (MHC) class I epitope indicated that as few as one third of such epitope variants were recognized by a given T cell receptor*(57, 63)*. These results are in agreement with theoretical predictions proposed for cross-recognition of MHC class I-presented peptides by T cell receptors*(67)*. Thus the use of a single natural isolate for a vaccine has a high risk of not protecting against a different clade, nor against many variants of the same clade.

A second approach to HIV-1 diversity derives vaccine immunogens from 'centralized' sequences, which employ consensus/average, or centre-of-the-tree*(68)* sequences or extrapolated aa to a common clade or group ancestor*(69)*. Centralized sequences are designed to minimize the sequence differences between a vaccine immunogen and circulating viruses*(68-71)*. So far they have proven immunogenic and able to elicit T cell responses in small animal studies*(68, 72-74)* and clinical trials*(75-77)*, providing experimental support for their further development. Early results for centralized immunogens for the entire group M are promising in that initial immunogenicity studies in mice yielded T-cell responses that were comparable to within-clade responses for many clades*(74)*, however, this strategy may be stretched too far for optimal coverage of $CD8^+$ T cell epitope variants of the whole group M*(62, 63, 78)*.

In a third approach, vaccines deliver a cocktail of immunogens derived from different clades*(79-81)*. While initial results have been encouraging and responses to each antigen in the cocktail were observed*(81)*, attention still needs be paid to possible immune interference, such as epitope antagonism, between different, but closely related peptide sequences in the vaccine, which may be limiting responses to some epitopes. Antagonism of T cell responses by altered epitope peptide ligands has been demonstrated both *in vitro(82, 83)* and *in vivo(62, 84-87)*. It can occur when a host capable of mounting a response to an agonist epitope is simultaneously exposed to an antagonist epitope variant, which interferes with the induction of the T cell response to the agonist epitope and leads to a defective response. Thus, the breadth of responses induced by cocktail approaches should be carefully monitored when such vaccines are used*(62)*.

A fourth approach uses computational methods for assembling a polyvalent vaccine candidate that optimize the coverage of T cell epitopes. 'Mosaic' immunogens*(88)* are based on intact proteins and retain the probability for natural processing and presentation of T cell epitopes. Their potential problems are similar to those of other cocktails of natural proteins, i.e. immune interference and inclusion of both variable and conserved regions, whereby responses to variable regions may draw attention away from potentially more useful conserved targets. The impact of these processes will be only resolved in vaccine studies. An alternative means of designing immunogens contending with the HIV-1 variation is the COT+ method*(89)*, which combines a central sequence with a set protein fragments designed to help cover diversity.

3.4 Design of the study immunogen HIVconsv

As elaborated above, protective T cells will have to recognize all HIV-1 variants circulating in the target population as well as reduce the chance of escape mutants in infected individuals. To address this problem, we designed a novel immunogen HIVconsv (for conserved) as a chimaeric protein assembled from the most highly conserved domains among the HIV-1 clade A, B, C and D proteomes. *(20)* First, a decision was taken that the HIVconsv gene should be

approximately 2.5 kbp in size, which makes it suitable for most currently used genetic vaccine vectors and is likely to support a high protein expression. 2,500 nucleotides translated into fourteen, 27- to 128-aa-long, most conserved regions of the HIV-1 proteins. The HIVconsv immunogen was assembled from segments derived from one of the four clade consensus sequences to reflect the fact that even the most conserved regions of HIV-1 are somewhat variable. It should be noted that because these regions are so highly conserved, often the consensus for one clade perfectly matched the consensus sequences of the other clades or indeed the group M, enhancing the potential for eliciting globally relevant cross-reactive responses. To keep the vaccine simple and minimize occurrence of immune interference of T cell responses while ensuring a good coverage of all the four major clades, the clades of individual segments were alternated in the 'string'. Epitopes recognized by rhesus macaque and mouse CD8 T cells, and a monoclonal antibody (mAb)*(90-92)* were added to the C-terminus of the HIVconsv immunogen to facilitate the vaccine pre-clinical development.

Alignments of the HIVconsv immunogen with the global HIV-1 sequences of group M including recombinant forms revealed that at least half of the sequences in the Los Alamos database are identical to segments 6 and 8 (the median distance = 0), while segments 2, 3, 10, 11, and 12 differ in less than 3% of their aa positions when compared to half of the sequences (median < 0.03). The largest distance from the circulating global sequences displayed segment 9 with differences in just over 7% aa positions. Conserved HIV-1 protein regions were included into the HIVconsv immunogen irrespective of whether of not they contained known T cell epitopes, however, every conserved fragment in the HIVconsv contains at least one known human epitope. In fact, 270 (24%) of the 1112 distinct published $CD8^+$ T cell epitopes smaller than 12 aa described in the Los Alamos HIV-1 database are embedded in these fragments. Even though most epitopes in the literature have been defined using clade B reagents and the HIVconsv immunogen is an assemblage of HIV-1 clade A, B, C, and D consensus fragments, still 192 (71%) of these 270 HIVconsv epitopes are identical to experimentally defined epitopes and additional 59 (22%) differ by only one aa, so that 251 (93%) epitopes differ by no more than a single-aa difference from a known epitope and thus may elicit a cross-reactive response.

All potential solutions to the problem of HIV-1 diversity have their theoretical advantages and disadvantages, the relevance of which will be only determined experimentally in efficacy trials in humans *(34)*. Indeed, as of November 2009, there are approximately 25 ongoing clinical vaccine studies mostly using multiple immunogens, of which only about 25% use 2 or more clades *(93)*.

3.5 Vaccine vectors and regimens

The frequency and quality of memory T cells induced by vaccination are to a large extent determined by the vaccine vectors and modalities employed to deliver the HIV-1-derived immunogens *(45, 94-96)*. While live, replication-competent virus vectors stir more vigorous immune responses *(97)*, their immunogenicity has to be balanced prudently with safety. The favoured delivery vectors at present are non-replicating genetic vaccines such as attenuated viruses or replicons that, similarly to replicating vaccines, deliver the immunogen to the MHC class I presentation pathway either directly or by cross-presentation*(98-100)* without producing infectious progeny*(101)*. Their lower immunogenicity*(102, 103)* cannot be easily enhanced by repeated boosts with the same vaccine because of a build-up of anti-vector immunity, which dampens insert-specific T cell induction*(104, 105)*. For this reason, heterologous prime-boost regimens are increasingly used*(19, 20, 106-110)*, although their limitations have not been properly, let alone systematically, tested. More complex vaccination regimens are less practical for global deployment, although not necessarily more logistically challenging than delivering highly active anti-retroviral therapy to resource and infrastructure-

limited countries, but may be instrumental in establishing proof-of-concept for T cell-based protection in humans.

The HIVconsv gene was made synthetically using ‘humanized’ amino acid codons. For the clinical studies, the gene was inserted into three leading non-replicating vaccine vectors: plasmid DNA to construct pSG2.HIVconsv, attenuated chimpanzee adenovirus to construct ChAdV63.HIVconsv and modified vaccinia virus Ankara to construct MVA.HIVconsv. For pre-clinical development and possible future regimen improvements, other vaccine modalities were also explored including attenuated human adenovirus HAdV-5 and synthetic long peptides, Semliki Forest virus replicons and *Mycobacterium bovis* bacillus Calmette–Guérin (BCG).

3.6 The investigational pSG2.HIVconsv DNA vaccine

3.6.1 Plasmid DNA as a vaccine vector

DNA vaccines contain a gene encoding one or more antigens under the regulation of a eukaryotic enhancer/promoter and polyadenylation signals that confer appropriate expression of the antigens. DNA encoding a selected component of a pathogen is injected as a plasmid e.g. pSG2.HIVconsv. When injected into muscle, cells surrounding the injection site internalize the plasmid and transport the DNA to the nucleus where transcription occurs as it would in natural infection. The feasibility of genetic vaccination has been shown in several experimental model systems, demonstrating that ‘naked’ nucleic acid vaccines may elicit both antibody and T cell-mediated responses.

3.6.2 Clinical experience with DNA vaccines

DNA vaccines have been used in a large number of clinical studies and shown to be safe and modestly immunogenic as a stand-alone vaccines *(107, 111, 112)*. In humans, plasmid DNA alone induced weak, but consistent responses mediated almost exclusively by CD4 T cells*(76, 113-115)*. Low immunogen production after DNA administration may be beneficial for the quality of the T cell responses, e.g. through induction of higher T cell avidity*(116)*. Thus, DNA is often used as a priming agent in heterologous prime-boost regimens with other vaccine modalities.

3.6.3 Clinical experience with pSG2 plasmid DNA-vectored vaccines

Plasmid pSG2 belongs to the pTH family of plasmids*(117)*, which all use a common expression cassette for the immunogen gene. This comprises the human cytomegalovirus early enhancer/promoter regions followed by intron A upstream and bovine growth hormone polyadenylation signal downstream of the open reading frame coding for the immunogen of interest. Plasmid pTH *(117)* carries the Ampicilline resistance gene and is used for preclinical development only. While pTHr*(118)* uses the operator-repressor titration (ORT) system of COBRA allowing plasmid maintenance without any use of antibiotics*(119)*, in plasmid pSG2 *(114)*, the Kanamycin resistance gene replaces that for Ampicilline resistance of the parental pTH plasmid. Both pTHr and pSG2 plasmid have been tested extensively in humans [and animals] as HIV-1 and malaria candidate vaccines, respectively, and shown to be safe and immunogenic*(107, 120)*. However, a pSG2 vector containing the HIVconsv insert has not been tested previously in man.

3.6.4 pSG2.HIVconsv dose and dosing schedule justification

Using our first generation prime-boost vaccines pTHr.HIVA plasmid DNA-MVA.HIVA, we have demonstrated that a dose of 4 mg of DNA intramuscularly administered into human

volunteers primed T cell responses more consistently than lower doses [reviewed in *(107)*). A number of non-human primate experiments and clinical trials using DNA priming showed that three DNA administrations deliver a potent T cell prime (*(114, 121)* and G. Pantaleo, G. Nabel, personal communication). Therefore, our preferred priming regimen in this study is three doses of pSG2.HIVconsv DNA at 4mg."

3.7 The investigational MVA.HIVconsv vaccine

3.7.1 Modified vaccinia virus Ankara

Modified vaccinia virus Ankara (MVA) is a vaccinia virus strain, which was attenuated by serial passage in chicken embryo fibroblasts. MVA has lost 15% of the parental genome, including immunomodulator genes and replicates well in chicken cells and baby hamster kidney cells, but grows poorly or not at all in most mammalian cells*(122)*.

3.7.2 Pre-clinical studies with MVA-vectored vaccines

A pre-clinical systemic toxicology study with an MVA.HIVA vaccine together with pTHr.HIVA DNA, both constructed in our laboratory, were carried out in compliance with Good Laboratory Practice (GLP) at Huntingdon Life Science, UK. A combined protocol of persistence, distribution and toxicity of the pTHr.HIVA and MVA.HIVA vaccines in the BALB/c mouse was performed, which demonstrated that the vaccines were non-toxic and detectable beyond 5 weeks after administration only in the sites of injection*(123)*. These results formed a basis for the approval of phase I safety and immunogenicity clinical trials in healthy HIV-1-uninfected volunteers. To support studies in HIV-1-infected subjects, toxicity and biodistribution of MVA.HIVA in mice with severe combined immunodeficiency (SCID) and simian immunodeficiency virus (SIV)-infected rhesus macaques were carried out, which demonstrated that the MVA.HIVA vaccine was non-toxic in mice and non-persistent in places other than the injection site in mice and monkeys *(124)*. The safety of another recombinant MVA was also studied in immune-suppressed macaques. Eight macaques were vaccinated with MVA by three different routes (intradermally, intramuscularly and intranasally) after immune-suppression by total body irradiation, anti-thymocyte globulin treatment or measles virus infection. No clinical, haematological or pathological abnormalities related to MVA inoculation were observed during a 13-day follow-up period *(125)*.

The immunogenicity of MVA-based recombinant vaccines in animals is well documented. Recombinant MVA vector vaccines alone or in combination with other vectors elicit insert-specific cytotoxic T lymphocytes (CTL) responses in mice *(20, 126, 127)* and rhesus monkeys *(128-130)*, decrease plasma viraemia and increase survival *(130, 131)* after challenge with pathogenic SIV. Several studies in macaques have demonstrated that, although CTL responses were detected following immunization with MVA-SIV recombinants, no animals were completely protected from infection upon challenge with pathogenic SIV. However, vaccinated animals had lower virus loads and prolonged survivals compared with control animals that received only non-recombinant MVA *(106, 130, 131)*.

3.7.3 Clinical experience with MVA-vectored vaccines

3.7.3.1 Safety of MVA-vectored vaccines in humans

Poxviruses have played an important role in the field of vaccinology ever since Edward Jenner in 1798 protected humans against smallpox (variola) by inoculation of the related cowpox virus. MVA, a highly attenuated strain of vaccinia virus and a member of the

Orthopoxvirus genus in the family of *Poxviridae*, made its debut towards the end of the smallpox eradication campaign as a safer alternative vaccine and has a good safety record *(132)*. It has been administered to more than 120,000 vaccinees as part of the smallpox eradication programme, with no reported adverse effects, despite the deliberate vaccination of high risk groups *(122)*. There are now safety data from a number of recombinant MVA-based vaccines expressing antigens for hepatitis B, malaria, TB, HIV, and melanoma from phase I/IIa trials both in the UK, Europe, Africa and Asia *(133-135)*.

Recombinant subunit vaccines vectored by non-replicating poxvirus MVA have an impressive record of safety from HIV-1/2-uninfected adults *(120, 136, 137)*, children*(133, 138-140)* and infants (McShane, personal communication). This is further supported by an excellent safety record of closely related non-replicating recombinant poxviruses NYVAC, ALVAC and fowlpox mostly in adults *(141-145)*, but also in children *(133, 138, 139)* and infants *(146)*.

3.7.3.2 Immunogenicity of MVA-vectored vaccines in humans

A number of MVA-vectored vaccines carrying HIV-1 and various other inserts have been or are being currently clinically assessed including MVA.HIVA which has been tested in over 300 healthy and HIV-1-infected individuals *(107)*. While in humans recombinant MVA is not a strong primer, it can boost efficiently pre-existing immune responses *(32, 75, 147)*.

MVA.HIVA vaccine delivered alone and in a prime-boost regimen was tested in several hundred healthy and HIV-1-infected volunteers in Europe and Africa, and the clinical immunogenicity results are summarized in a recent publication*(107)*. Overall, these trials demonstrated that MVA.HIVA vaccine is less efficient in priming HIV-1-specific responses, but it can deliver a consistent boost to both $CD4^+$ and $CD8^+$ T cells, which is particularly strong if the T cell responses are well primed, e.g., HIV-1 infected patients *(32, 75, 148)*. Thus, in the most recent trial in healthy volunteers, MVA.HIVA alone primed responses were not detectable using the *ex vivo* IFN-γ ELISPOT assay, but in cultured IFN-γ ELISPOT assays, HIV-1-specific memory T cells were found in 5 out of 8 volunteers. When primed with a pTHr.HIVA DNA vaccine, MVA.HIVA induced detectable memory HIV-1-specific predominantly $CD4^+$ T cell responses in 8 out of 8 volunteers *(76)*. In HIV-1 infected patients on highly active antiretroviral treatment (HAART), a significant amplification and broadening of $CD8^+$ and $CD4^+$ IFN-γ responses to vaccine-derived epitopes was observed in all (n=16) vaccinees, but not in unvaccinated controls, in the absence of rebound viraemia. After MVA.HIVA administration, vaccine-expanded $CD8^+$ T cells identified by tetramer reactivity were transiently activated and had upregulated perforin levels. Expansions persisted for at least one year and consisted predominantly of either $CD45RA^-$ $CCR7^+$ or $CD45RA^-$ $CCR7^-$ $CD8^+$ T cells. Increased frequencies of $CD4^+$ T cells expressing intracellular IL-2 and IFN-γ were noted in several vaccinees *(75)*.

3.7.4 Clinical experience with MVA.HIVconsv

The HIV-CORE001 trial started in January 2010 testing the safety and immunogenicity of three MVA.HIVconsv immunisations, at either ‘standard’ or higher doses (1 x 10^8 pfu or 4 x 10^8 pfu, respectively), administered by intramuscular injection to HIV-1-seropositive patients on HAART. Preliminary safety and immunogenicity data show no SAE related to the vaccine to date.

3.7.5 MVA.HIVconsv dose justification

In EudraCT 2007-004567-21 (Vac036; CI: Adrian Hill), systemic reactions were observed using MVA AMA1 initially at a dose believed to be 2.5 x 10^8 PFU, but on further analysis

(including multiple re-titrations) this turned out to be 5 x10^8 PFU. This showed a fairly severe reactogenicity profile in several immunised subjects and the dose was halved. On administration of this halved dose (of 2.5x10^8 PFU) to one subject, reactogenicity was noted again and the dose was halved again for further vaccinees to a dose of 1.25 x 10^8 PFU. This dose has now been administered to 21 subjects in Vac036 and Vac039 with a good safety profile. Because similar experience was obtained in EudraCT no. 2006-005966-37, in which MVA.NP+M1 was administered alone (without a recombinant ChAdV-63 boost), the initially observed systemic reactions were most likely caused by rMVA alone.

In HIV-CORE001, two to three doses of 1 x 10^8 PFU of MVA.HIVconsv administered intramuscularly were safe in 8 volunteers so far. HIV-CORE002 will use the same batch of the MVA.HIVconsv vaccine and we have decided to lower the dose from the originally proposed 4 x 10^8 to 2 x 10^8 PFU MVA.HIVconsv delivered intramuscularly, i.e. to double the safe dose of MVA.HIVconsv in HIV-CORE001. We believe this dose will be safe and will not cause any systemic reactions.

3.8 The investigational ChAdV63.HIVconsv vaccine

3.8.1 Attenuated adenoviruses as vaccine vectors

Selected attenuated adenoviruses are proving to be highly immunogenic vaccine vectors *(149, 150)*. A drawback for the use of human adenovirus serotype 5 (HAdV-5), which was the leading vector until recently, is pre-existing humoral immunity, with seroprevalence as high as 90% in parts of Africa *(150, 151)*. To circumvent this problem, a number of chimpanzee adenovirus serotypes have been explored; these are unaffected by human pre-existing antibodies to human adenoviruses and are able to induce transgene-specific T cell responses of comparable or greater magnitude than those induced by Merck's AdHu5 vectored HIV-1 vaccine tested in the STEP trial *(150, 151)*. We have chosen ChAdV-63 which was isolated and developed as a vector by Okairos *(152)*. The genomic DNA of the adenovirus was cloned into bacterial plasmids to eliminate the possibility of carrying over any adventitious infectious agents from the original hosts. The virus was rendered replication incompetent by deletion of the E1 gene. Their immunogenicity has been increased by deletion of the E3 region and their genomes can stably accommodate passenger gene (s). HEK293 cells can be used for preparation of high titre Good Manufacturing Practice (GMP) virus stocks without the risk of generation of contaminating replication-competent adenovirus (RCA) forms.

3.8.2 Pre-clinical studies with chimpanzee adenovirus-vectored HIVconsv vaccines

In pre-clinical studies in mice and rhesus macaques, the ChAdV63.HIVconsv vaccine was found to be safe and immunogenic. In a dose escalation experiment in mice (10^5 to 10^9 virus particles per dose), the frequencies of HIV-1-specific T cells increased in a dose-dependent manner. In macaques, the ChAdV63.HIVconsv vaccine significantly boosted transgene-specific T cells in optimally-primed animals.

3.8.3 Clinical trials with chimpanzee adenovirus-vectored vaccines

Experimental vaccines vectored by chimpanzee adenoviruses (have been trialed in UK and Africa since in 2007 and over 130 volunteers have been dosed. The first experimental human vaccine were vectored by ChAdV-63 and ChAdV-3. These carried malaria (*Plasmodium falciparum*) and hepatitis C virus immunogens, respectively. So far, the vaccines have been well tolerated and highly immunogenic.

3.8.4 -ChAdV63.HIVconsv dose justification

Vaccines vectored by non-replicating ChAdVs at a dose of 5 x 10^{10} vp, have now been injected intramuscularly into over one hundred volunteers in Oxford and have been shown to be safe and immunogenic. Higher doses did not strongly increase CD8 T cell induction (Adrian Hill, personal communication). In rhesus macaques 5 x 10^{10} vp efficiently boosted well-primed CD8 T cell responses *(121)*. The ChAdV-63.HIVconsv vaccine has not been tested in man before. Therefore, for HIV-CORE002, the ChAdV63.HIVconsv vaccine will be administered at 5 x 10^{10} vp because of the proven safety and immunogenicity of chimpanzee adenoviral vectors at this dose.

3.9 Pre-clinical studies of HIVconsv vaccines in heterologous regimens

Heterologous vaccination regimens using pTH.HIVconsv plasmid DNA (D), HAdV5.HIVconsv (A) and MVA.HIVconsv (M), were tested in BALB/c and *HLA-A*0201*-transgenic mouse models: we demonstrated that the murine epitopes in the HIVconsv immunogen could be efficiently processed and presented for recognition by CD8+ T cells and that DAM sequence was more immunogenic than other combinations of these three vaccines (note that at this point, the ChAdV63.HIVconsv vaccine was not available)*(20)*. Induction of high HIV-1-specific T cell frequencies by the DDDAM regimen was subsequently confirmed in rhesus macaques *(121)*. In this model, the vaccine-elicited T cells showed broad specificities, were polyfunctional, polyclonal, readily proliferated to recall antigens and recognized majority of HIV-1 variant epitope sequences present in the Los Alamos National Library database *(153)*. Furthermore, we also demonstrated the macaque immunogenicity of the newly available ChAdV63.HIVconsv vaccine in the DDDCM regimen in this animal model, mimicking this proposed clinical study (manuscript in preparation). Virus challenge studies could not be performed in the HIVconsv-vaccinated animals could not be challenged because the HIVconsv vaccines are designed for humans and derived from HIV-1 antigens, and HIV-1 does not replicate in rhesus macaques.

3.10 Pre-clinical GLP toxicology using HIVconsv vaccines

Two separate pre-clinical GLP toxicology studies have been carried out by Huntingdon Life Sciences. Study UNO0011 assessed the systemic toxic potential of 2 x 10^7 pfu/dose of MVA.HIVconsv administered by intramuscular injection to groups of 10 male and 10 female BALB/c mice on days 1, 15 and 29 (the MMM regimen). Vaccinations were not associated with any systemic toxicological changes. The findings of increased cellularity of the draining lymph nodes, high plasma gamma globulin, concentration and aspartate aminotransferase activity and inflammatory changes at the dose sites were considered to be consistent with a predicted response to vaccine administration. These results supported the MHRA approval for clinical trial HIV-CORE 001 (see below).

Study UNO0012 assessed in the similar groups of BALB/c mice the systemic toxic potential following administration of 50 µg/dose of pSG2.HIVconsv administered by intramuscular injection on days 1, 15 and 29, followed by an intramuscular injection of 5 x 10^9 vp ChAdV63.HIVconsv on day 43 (the DDDC regimen). Again, vaccinations were not associated with any systemic toxicological changes and all the observed lymph node changes were consistent with response to the vaccine administration. The UNO0011 toxicology protocol MMM together with the UNO0012 protocol DDDC support the application for the proposed Clinical Protocol HIV-CORE 002 as confirmed in communication with the Medicines and Healthcare products Regulatory Agency (MHRA).

4 STUDY OBJECTIVES

4.1 Primary Objectives

- To evaluate the short-term safety of one low dose ChAdV63.HIVconsv delivered intramuscularly into healthy, low-risk, HIV-1-uninfected adult volunteers.
- To evaluate the safety of the pSG2.HIVconsv DNA, ChAdV63.HIVconsv and MVA.HIVconsv vaccines administered sequentially by intramuscular needle injection in heterologous prime-boost regimens into healthy, low-risk, HIV-1-uninfected adult volunteers.

4.2 Secondary Objectives

- To evaluate the immunogenicity of the pSG2.HIVconsv DNA, ChAdV63.HIVconsv and MVA.HIVconsv vaccines administered sequentially by intramuscular needle injection in three different heterologous prime-boost regimens into healthy, low-risk, HIV-1-uninfected adult volunteers.
- To compare the immunogenicity (breadth, specificity and HIV-1 clade coverage of T cell responses) of two regimens DDDCM and DDDMC.
- To determine whether priming with three DNA vaccinations (DDDCM regimen) is necessary for optimal T cell responses to the HIVconsv immunogen by comparing responses to the DDDCM and ChAdV63/MVA (CM) regimens.
- To assess of the kinetics, magnitude and functionality of vaccine-induced T cell responses elicited by the vector ChAdV63.

As this is a first in human study for two of the vaccines, this trial has been designed as a pilot study to compare different vector combinations and sample sizes have been chosen that will only allow detection of large response differences among volunteer stages 2-4. Thus, it is expected to yield data which are primarily descriptive. The inclusion of placebos will ensure that any potential for bias in the analysis of immune responses is minimised and will give greater confidence to assignations of the causality of any adverse reactions observed in this study and will help to maintain blinding of the study participants.

5 STUDY DESIGN

5.1 Summary of study design

HIV-CORE 002 is a randomised, placebo-controlled, single-blind study designed to evaluate the safety and immunogenicity of three novel HIVconsv vaccines.

Table 1. *Phase I trial design*

Stage	Regimen	No.	Wk 0	Wk 4	Wk 8	Wk 12	Wk 16	Wk 20
1	c	2	c	-	-	-	-	-
2	CM	8	C	-	M	-	-	-
	PP	2	P	-	P	-	-	-
3	DDDCM	8	D	D	D	C	-	M
	PPPPP	2	P	P	P	P	-	P
4	DDDMC	8	D	D	D	M	C	-
	PPPPP	2	P	P	P	P	P	-

c – $5x10^9$ vp ChAdV63.HIVconsv i.m.; C – $5x10^{10}$ vp ChAdV63.HIVconsv i.m.; M – $2x10^8$ pfu MVA.HIVconsv i.m.; D – 4 mg pSG2.HIVconsv DNA i.m.; and P – placebo i.m.

Stage 1 The first stage will start from a low dose of $5x10^9$ vp of ChAdV63.HIVconsv which is expected to be well tolerated but of sub-optimal immunogenicity. **Stage 2** Once the safety of the low dose is confirmed (2 weeks), the next 10 volunteers will be randomized to either $5x10^{10}$ vp of ChAdV63.HIVconsv (predicted to be the most immunogenic dose while still tolerated) followed by boost with $2x10^8$ pfu of MVA.HIVconsv (CM; n=8) or two placebo injections (PP; n=2). **Stage 3** The next 10 volunteers will be randomized to either three doses of 4 mg of pSG2.HIVconsv DNA followed by a boost with $5x10^{10}$ vp of ChAdV63.HIVconsv and a second boost with $2x10^8$ pfu of MVA.HIVconsv (DDDCM; n=8) or five placebo injections (PPPPP; n=2). **Stage 4** Finally, the last group of 10 volunteers will be randomized to either three doses of 4 mg of pSG2.HIVconsv DNA followed by boost with $2x10^8$ pfu of MVA.HIVconsv and a second boost with $5x10^{10}$ vp of ChAdV63.HIVconsv (DDDCM; n=8) or placebo (PPPPP; n=2). The schedule of bleeds is outlined in Appendix A.

5.2 Study Duration

16 months (from screening of first volunteer to 6 months after last immunisation of last volunteer, assuming approximately 2 recruits per week)

5.3 Primary and secondary endpoints/outcome measures

5.3.1 Primary Endpoints

The safety of the vaccines will be determined by analysis of local and systemic reactogenicity, and biochemical and haematological data.

The data will be expressed as
- Proportion of volunteers who develop a grade 3 or 4 local reaction.
- Proportion of volunteers who develop a grade 3 or 4 systemic reaction.

5.3.2 Secondary Endpoints

Safety and tolerability secondary endpoints
- A descriptive summary of grade 3 of 4 local and systemic events including laboratory abnormalities.
- A descriptive summary of serious adverse events, including laboratory abnormalities

Immunogenicity secondary endpoints
- Proportion of volunteers who develop CD8+ T cell responses to one or more HIV-1 epitopes, as determined by IFN-γ ELISPOT assay
- The cumulative number of epitopes volunteers' CD8+ T cells recognize, as determined by IFN-γ ELISPOT assay
- The total frequency of HIV-1-specific T cell responses, as determined by IFN-γ ELISPOT assay

Exploratory secondary endpoints
- Evaluation of the in vitro HIV-1 suppressive capacity of vaccine-elicited CD8+ T cells, using a novel flow cytometric assay
- Polyfunctionality of vaccine-elicited HIV-1-specific CD8+ T cells as determined by multicolour flow cytometry on selected volunteers at selected time points

- Proliferative capacity of vaccine-elicited HIV-1-specific CD8+ T cells as determined by CFSE proliferation assays
- Functional avidity of vaccine-elicited HIV-1-specific CD8+ T cells as determined by specific peptide titration in IFN-γ ELISPOT assay on selected volunteers at selected time points

5.4 Study Population

5.4.1 Overall description of trial participants

Development of an effective prophylactic vaccine against HIV is the best solution to the control of the HIV/AIDS epidemic. Furthermore, testing immunogenicity of vaccines in patients with compromised immune responses may not properly reflect the safety and immunogenicity of vaccines in healthy volunteers. The good safety profiles in humans of the vaccines also strongly support evaluation of our vaccine regimens first in HIV-1-negative volunteers rather than in HIV-1-infected patients.

All 32 volunteers in this trial will be deemed to be at low risk of HIV-1 infection. As far as possible equal numbers of adult men and women will be recruited to these studies and members of different racial and ethnic groups enrolled commensurate with their representation in the population of Oxford. Subjects will be adults aged 18-50 who fully comprehend the purpose and details of this study as provided in the Participant Information Sheet and are able to provide informed consent. Eligibility will depend on the results of laboratory tests, review of medical histories, physical exam results and answers to questions about risk behaviours.

5.4.2 Inclusion Criteria

1) Healthy males or females, as assessed by a medical history, physical examination and laboratory tests.
2) Aged at least 18 years on the day of screening and no greater than 50 years on the day of the first vaccination.
3) Willing to comply with the requirements of the protocol and available for follow-up for the planned duration of the study.
4) In the opinion of the principal investigator or designee, the volunteer has understood the information provided. Written informed consent must be given before any study-related procedures are performed.
5) Willing to undergo HIV-1 testing, HIV-1 counselling and receive HIV-1 test results.
6) If heterosexually active female; using an effective method of contraception (e.g. hormonal contraception, diaphragm, intra-uterine device (IUD), condoms, anatomical sterility in self or partner) from 14 days prior to the first vaccination until at least 6 weeks after the last vaccination; all female volunteers must be willing to undergo urine pregnancy tests at time points specified in the Schedule of Procedures (Appendix A).
7) If heterosexually active male; willing to use an effective method of contraception (condoms; anatomical sterility in self or partner) from the day of the first vaccination until 6 weeks after the last vaccination.
8) Willing to forgo donating blood during the study.

5.4.3 Exclusion Criteria

Any relevant abnormality on history or examination including history of immunodeficiency or autoimmune disease, or use of systemic corticosteroids, immunosuppressive, antiviral, anticancer or other medication that, in the opinion of the principal investigator or designee, is

clinically significant, within the previous 6 months. (Note: use of inhaled steroids for asthma and use of topical steroids for localized skin conditions will not exclude a volunteer from participation.)

1) Any clinically significant acute or chronic medical condition that is considered progressive or, in the opinion of the principal investigator or designee, would make the volunteer unsuitable for the study.
2) Any of the following abnormal laboratory parameters listed below:

Haematology
- Haemoglobin < 10.0 g/dl
- Absolute Neutrophil Count (ANC) ≤ 1000 /mm^3 (≤ 1 x 10^9 /l)
- Absolute Lymphocyte Count (ALC) ≤ 600 /mm^3 (≤ 1 x 10^9 /l)
- Platelets ≤100,000 /mm^3, ≥ 550,000 /mm^3 (≤ 90 /l, ≥ 550 /l)

Biochemistry
- Creatinine > 1.3 x ULN
- Aspartate aminotransferase (AST) > 2.5 x ULN
- Alanine aminotransferase (ALT) > 2.5 x ULN

Urinalysis
- Abnormal dipstick <u>confirmed</u> by microscopy

3) Reported high-risk behaviour for HIV-1 infection. High-risk behaviour for HIV-1 infection is defined as follows. Within the previous 6 months the volunteer has:
   - Had unprotected vaginal or anal sex with a known HIV-1-infected person or a casual partner (i.e., no continuing, established relationship)
   - Engaged in sex work for money or drugs
   - Used injection drugs
   - Acquired one of the following sexually transmitted infection; Chlamydia, gonorrhoea and syphilis.
4) Confirmed HIV-1 or HIV-2 infection.
5) If female, pregnant or planning a pregnancy within 6 weeks after last vaccination; or lactating.
6) Receipt of live attenuated vaccine within the previous 60 days or planned receipt within 60 days after vaccination with Investigational Medicinal Product (IMP) or receipt of other vaccine, including influenza vaccine, within the previous 14 days or planned receipt within 14 days after vaccination with the IMP.
7) Receipt of blood transfusion or blood products within the previous 6 months.
8) Participation in another clinical trial of an IMP currently or within the previous 3 months or expected participation during this study.
9) Receipt of any investigational HIV-1 vaccine within the last 6 years.
10) History of severe or very severe local or systemic reactogenicity events, or history of severe or very severe allergic reactions.
11) Confirmed diagnosis of acute or chronic hepatitis B virus infection (spontaneous clearance leading to natural immunity, indicated by antibodies to core + antigens, is not an exclusion criterion); confirmed diagnosis of hepatitis C virus infection; untreated syphilis.
12) Smallpox vaccination within the previous 3 years (smallpox vaccination prior to 3 years should be documented but is not an exclusion criterion).
13) Major psychiatric illness including any history of schizophrenia or severe psychosis, bipolar disorder requiring therapy, suicidal attempt or ideation in the previous 3 years.

5.5 Recruitment

Potential participants will be recruited through advertisements in local newspapers, hospitals and university colleges. Interested persons will be offered an informal discussion with the Principal Investigator, research nurse or research physician, when they will be provided with information on the vaccine and the study protocol and will be given a copy of the Participant Information Sheet. They will have the opportunity to ask questions and to arrange an appointment for a screening visit if they wish to take part. Volunteers recruited in the study will be free to leave the study at any time.

5.5.1 Reimbursement

Volunteers will be reimbursed for their time, effort and travel costs to the study site due to study participation. Reimbursement amounts will be documented in the Participant Information Leaflet.

5.6 Study Procedures

5.6.1 Study visits

Approximately 600ml of blood will be drawn over a period of 6 months including samples prior, throughout and regularly after the vaccinations. These will be at screening, day 0 and at a number of days following vaccination to capture the peak and persistence of vaccine induced-responses *(76,79)*. Finally, blood will be drawn at the end of the study. See Appendix A for schedule of procedures.

At screening (Visit 1, up to 28 days prior to day 0 and at least 24 hours after giving the volunteer the Participant Information Sheet), site personnel (trial nurse/physician) will perform the following:

- Review the Participant Information Sheet and Consent Form prior to obtaining written informed consent.
- Obtain written informed consent before performing any study procedures.

If the volunteer agrees to participate, site personnel will:

- Give the volunteer opportunity to ask any additional questions about study procedures
- Confirm study eligibility
- Obtain a complete medical history including concomitant medication (prescribed and non-prescribed)
- Perform a directed physical examination including weight and vital signs; oral temperature will be recorded and resting pulse and blood pressure (BP) will be measured after the participant has sat for at least five minutes
- Obtain a blood sample for laboratory investigations indicated in the Schedule of Procedures (Appendix A)
- Perform a urine pregnancy test in all female participants (except when documentation of physiological or anatomical sterility is available).

Screening laboratory test (s) may be repeated once at the discretion of the principal investigator or designee to investigate any isolated abnormalities.

If the screening visit occurs more than 28 days prior to the date of vaccination all screening procedures must be repeated. In such cases the complete medical history may be

replaced by an interim medical history and the Informed Consent Document should be reviewed, if necessary.

If the volunteer gives his/her permission, the volunteer's GP will be informed that the volunteer is participating in the study.

5.6.2 Vaccination visit

Volunteers will receive vaccine or placebo on visits depending in which study stage he/she is enrolled. These visits are specified in the Schedule of Procedures (Appendix A).

Prior to the first vaccination, site personnel will:

- Review interim medical history
- Perform baseline assessment of planned injection site and systemic symptoms
- Review any adverse events and concomitant medications
- Review safety laboratory data from the previous visit
- Perform a directed physical examination including vital signs (pulse, respiratory rate, blood pressure and temperature), examination of vaccination site and axillary lymph nodes in addition to any further examination indicated by history or observation
- Collect blood and urine specimens for tests as specified in the Schedule of Procedures (Appendix A)
- Perform a pregnancy test for all female volunteers and obtain results prior to vaccination
- Issue diary card (for the recording of temperature between 0-3 days post-vaccination) and provide instructions on the recording of information
- The volunteer will be enrolled by assignment of an allocation number.

For subsequent vaccination visits, site personnel will follow the procedures listed above as and when indicated in the Schedule of Procedures.

After vaccination, site personnel will:

- Cover the vaccination site with an semi-occlusive dressing and observe the subjects closely for 30 minutes for signs of acute reaction
- Monitor vital signs after 30 minutes
- Record any local reaction at the site of vaccine administration (Appendix G)
- Contact subjects by telephone after 3 days and review them if necessary
- Subjects will record body temperature on a diary card for 3 days after each vaccination (Appendix E)

5.6.3 Follow-Up Visits (including Final or Early Termination visit)

Where specified in Schedule of Procedures (Appendix A), site personnel will:

- Review the diary card and retain in the CRF
- Solicit and record adverse events
- Review and record interim medical history and concomitant medication
- Perform a directed physical examination including vital signs
- Assess the vaccination site on days indicated in the Schedule of Procedures
- Obtain a blood sample for laboratory investigations indicated in the Schedule of Procedures
- Supplementary visit (s) for further investigation may be planned at the discretion of the principal investigator or designee. Supplemental visit (s) may be recommended if clinically indicated or to clarify observations.

5.6.4 Final Visit or Early Termination Visit

The Final Visit or Early Termination Visit procedures will be performed according to the Schedule of Procedures (Appendix A).

Site personnel will:
- Review any adverse events and concomitant medications
- Perform a directed physical examination including vital signs, (pulse, respiratory rate, blood pressure and temperature), examination of vaccination site and axillary lymph nodes in addition to any further examination indicated by history or observation
- Assess any local and systemic reactogenicity events
- Collect blood for tests as specified in the Schedule of Procedures
- Perform a pregnancy test in all female volunteers where appropriate

5.6.5 Unscheduled visits

Visits / contacts other than those described in the Schedule of Procedures will be classified as unscheduled visits and recorded on a designated CRF. They may occur:
- For administrative reasons
- To review a laboratory investigation from a previous visit
- To review the outcome of an adverse event
- To conduct a study visit where a volunteer has missed the scheduled study visit window
- For any other reason requested by the volunteer or Principal Investigator

5.6.6 Informed Consent

Potential participants will be presented with the Participant Information Sheet (Appendix C) and its content will be reviewed with them in person to enable discussion of: the exact nature of the study; the implications and constraints of the protocol; the known side effects and any risks involved in taking part. It will be clearly stated that the participant is free to withdraw from the study at any time for any reason without prejudice to future care, and with no obligation to give the reason for withdrawal. Participants will be allowed as much time as wished to consider the information, and to question the Principal Investigator, their GP or other independent parties to decide whether they will participate in the study.
Written Informed Consent will then be obtained by means of participant dated signature and dated signature of the person who presented and obtained the informed consent (Appendix D). The person obtaining the consent will be suitably qualified and experienced and will have been authorised to do so by the Principal Investigator. A copy of the signed Informed Consent Form will be given to the participants and the original will be kept in a locked filing cabinet. The original signed form will be retained at the study site in a locked cabinet.

5.6.7 Counseling regarding contraception & use of condoms

Site personnel will counsel study participants about the importance of preventing pregnancies and about effective methods of contraception including correct use of condoms. Condoms will be offered to study participants where appropriate.

5.6.8 Medical history and physical examination

At screening, a comprehensive history of previous illness and any medical or surgical interventions will be recorded. A directed physical examination will be performed at the visits specified in the Schedule of Procedures or as clinically indicated. This may include weight,

vital signs, examination of skin, mucous membranes, lymph nodes, respiratory, cardiovascular, abdominal or central nervous systems as indicated by history or observation.

5.6.9 Blood and urine collection

The volume of blood to be taken at each visit will vary from 40-80 ml and will not exceed 600 ml in any 6-month period. Blood will usually be collected from a vein in the antecubital fossa. In the event of an abnormal laboratory value, an additional sample may be collected if clinically indicated. Urine will be analysed using a standard NHS dipstick test at screening. A urine pregnancy test will be performed as indicated in the Schedule of Procedures.

5.6.10 Laboratory results

All diagnostic laboratory (Oxford Radcliffe Hospitals NHS Trust laboratories) results will be reviewed and the reports signed by the Principal Investigator who will record in the CRF whether they are normal, abnormal but not clinically significant, or abnormal AND action is required. In the latter case the eligibility of the participants will be reviewed.

5.7 Randomization

Once eligibility has been confirmed, subject numbers will be assigned sequentially as each subject enters the study. The study participants in each group will be assigned vaccine or placebo through a randomisation schedule based on the randomisation plan. Laboratory personnel and volunteers will be blinded with respect to the allocation of vaccine or placebo within the groups. Those giving the vaccine will not be blinded as the study vaccines are open-labelled. They will ensure blinding of volunteers by shielding vaccine vials during preparation and administration.

Randomisation will be performed centrally at the Centre for Statistics in Medicine in Oxford (CSM) using dedicated computer software. Following recruitment and consent, participants will be randomised to either vaccine or placebo. The statistician carrying out the randomisation will have no direct contact with participants.

5.8 Unblinding procedures

Unblinding of an individual volunteer is indicated in the event of a medical emergency where the clinical management of the volunteer would be altered by knowledge of the group assignment. The decision to unblind will be taken in conjunction with the independent members of the DMC who will be available in case of emergency. Procedures and contact details for unblinding procedures will be held in a site-specific SOP. The site personnel will ensure that the reasons for unblinding document are documented in the CRF. Volunteers will also be given contact details of trial staff to be contacted in an emergency.

5.9 Definition of End of Study

The end of the study is the date of the last visit of the last participant.

6 INVESTIGATIONAL MEDICINAL PRODUCT

6.1 Description

All three vaccines and placebo are manufactured under Good Manufacturing Practice

conditions and in compliance with European Union GMP Guidelines, Annex 13. The vaccines will be open-labeled and therefore shielded from view of participants to ensure blinding. The immunology laboratory will also remain blinded.

pSG2.HIVconsv DNA is manufactured, labelled and released to trial by Bristol Institute for Transfusion Sciences Clinical Biotechnology Centre, Bristol, UK.

ChAdV63.HIVconsv is manufactured, labelled and released to trial by the Clinical Biomanufacturing Facility (CBF), Churchill Hospital, Oxford.

MVA.HIVconsv is manufactured by IDT Biologika GmbH, Germany. MVA.HIVconsv will be shipped to CBF and will then be labelled and released to trial.

The placebo for all three vaccines will be single use sodium chloride (NaCl) 0.9% w/v purchased from the Pharmacy, John Radcliffe Hospital, Oxford.

Table 2 *Description of IMPS and Placebos*

Vaccine/Placebo	Dosage	Formulation	Volume injected (approximate)
pSG2.HIVconsv DNA	4 mg	Phosphate buffered saline, pH 7.4	1ml
ChAdV63.HIVconsv	$5x10^9$ vp (stage 1)<br>$5x10^{10}$ vp (stage 2,3,4)	10 mM histidine, 7.5% sucrose, 35mM NaCl, 1 mM $MgCl_2$, 0.1% PS80, 0.1 mM EDTA, 0.5% EtOH, pH 6.6	30 µl (stage 1)<br>300 µl (stage 2,3,4)
MVA.HIVAconsv	$2x10^8$ pfu	10 mM Tris-HCl,<br>140 mM NaCl, pH 7.7	200 µl
Placebo	N/A	NaCl 0.9% w/v	Matched to IMP

6.2 Packaging, Storage and Shipment

The vaccines will be supplied in glass stoppered vials and labelled under GMP in order to support a single-blind trial and in compliance with regulatory requirements, including as a minimum: the study number, manufacturer, route of administration, volume, storage temperature and 'for clinical trial use only'. Release of the IMP will be in compliance with procedures required by ICH-GCP. Authorisation to ship the IMP to the site will be given in writing by the Principal Investigator upon confirmation that all critical documents required for shipment authorisation are completed. The IMP will be shipped to the trial site on dry ice with temperature logging. On arrival they will be transferred to monitored storage between -70°C and -90°C at the Centre for Clinical Vaccinology and Tropical Medicine, Churchill Hospital. The placebo will be stored at room temperature.

6.3 Dispensing and Handling

- The vaccines will be dispensed according to site- and study-specific SOPs.
- All vaccines will be used as supplied by the manufacturer with no further preparation.
- The vaccines will be thawed until completely liquid by holding the vial in the hand.
- The vials will not be shaken and the required volume will be drawn into the appropriate syringe.
- pSG2.HIVconsv DNA, MVA.HIVconv and ChAdV63.HIVconsv will be administered within 1 hour of thawing.

6.4 Administration

- The vaccines and placebo will be administered by intramuscular injection at the time-points specified in the Schedule of Procedures (Appendix A).
- The low dose of ChAdV63.HIVconsv will be preferably administered into the deltoid region of the non-dominant arm.
- All other doses will be injected into deltoid regions of both arms.
- The injection site will be observed for 30 minutes
- Complete instructions for the handling and administration of IMPs are supplied in the site- and study-specific SOPs.

6.5 Accountability and Disposal

- During the study an accountability log, dispensing log and log of used vials will be kept.
- During the study the used and unused vials, accountability log, dispensing log and the log of used vials will be monitored according to ICH-GCP.
- All used vials will be stored in a designated local freezer at the end of each vaccination visit.
- At the end of the study the used and unused vials will be transferred to Dr Hanke's laboratory.

7 ASSESSMENTS

7.1 Safety Assessments

- Data on local and systemic reactogenicity events will be collected using specific questions.
- Data on other events will be collected with an open question.
- All data will be recorded in the source documents.

7.1.1 Local Reactogenicity Events

- The presence of local reactogenicity events will be assessed at the time points specified in the Schedule of Procedures (Appendix A).
- Local reactogenicity events will be collected prospectively by structured interviews at the vaccination visits and post-vaccination follow-up visits.
- Local reactogenicity events (pain, tenderness, erythema or skin discoloration, skin damage (vesiculation or ulceration), induration (formation of crust or scab) will be assessed and graded according to the Adverse Event Grading Toxicity Table (Appendix B).

7.1.2 Systemic Reactogenicity Events

The presence of systemic reactogenicity events will be assessed at the time points specified in the Schedule of Procedures (Appendix A).

- Volunteers will record their temperature between 0-3 days post-vaccination in a study-specific diary.
- Systemic reactogenicity events will be collected prospectively by inspection of the diary and structured interviews at the vaccination visits and post-vaccination follow-up visits.
- Vital signs (pulse, respiratory rate, blood pressure and temperature) will be measured by site personnel prior to vaccination and approximately 30 minutes post-vaccination.
- Feverishness, chills, headache, nausea, vomiting, malaise and myalgia will be graded according to the Adverse Event Grading Toxicity Table (Appendix G).

7.1.3 Other Adverse Events

Occurrence of other adverse events (including Serious Adverse Events) will be collected following an open question to volunteers at the time points specified in the Schedule of Procedures (Appendix A). The adverse events will be graded as specified in the Adverse Event Grading Toxicity Table (Appendix G).

7.1.4 Routine Laboratory Parameters

Table 3 shows the laboratory parameters that will be measured routinely. These will include haematology, biochemistry, immunological assays and urinalysis. The samples for these tests will be collected at the time points specified in the Schedule of Procedures (Appendix A).

Table 3 *Routine Laboratory Parameters*

**Laboratory Parameter**	**Test**
Haematology	Full blood count (haemoglobin, haematocrit, erythrocytes, leucocytes, platelets)<br>Differential count (absolute neutrophils, absolute lymphocytes)
Biochemistry	Liver function tests: (aspartate aminotransferase (AST), alanine aminotransferase (ALT), total and direct bilirubin)<br><br>Kidney function tests: (creatinine) and urinalysis (see below)
Urinalysis	Dipstick test for protein, blood, glucose, ketones, leucocytes, nitrite. If abnormalities (blood, protein, leucocytes) are found in the dipstick test then further tests will be performed e.g. microscopy and culture

7.1.5 Specific Screening Tests

Volunteers will be screened to exclude the following diseases:

- Hepatitis B: positive for hepatitis B surface antigen (HbsAg)
- Hepatitis C: positive for hepatitis C antibodies (HCV antibodies)

- Syphilis: Treponemal IgG/IgM and positive RPR/TPPA AND no documentation of adequate treatment.

7.2 Immunogenicity Assessments

7.2.1 Cellular Responses

T cell responses will be determined initially by IFN-γ ELISPOT assay and, depending on the number of cells available, by further exploratory assays including investigated by Intracellular cytokine staining and *in vitro* HIV suppression assays.

7.3 Other Assessments

7.3.1 HLA Typing

HLA typing will be performed on samples collected at the time point specified in the Schedule of Procedures (Appendix A). The local laboratory will perform the analyses in accordance with site-specific SOPs.

7.3.2 HIV Antibody Test

Samples will be tested by standard ELISA at the time points specified in the Schedule of Procedures (Appendix A).

7.3.3 Pregnancy Test

A urine pregnancy test for all female volunteers where appropriate will be performed by measurement of Human Chorionic Gonadotrophin (βhCG) at the time points specified in the Schedule of Procedures (Appendix A). If found to be pregnant, the volunteer would be withdrawn from the trial.

8 SAFETY REPORTING

8.1 Definitions

8.1.1 Adverse Event (AE)

An AE or adverse experience is:
Any untoward medical occurrence in a patient or clinical investigation participants administered a medicinal product, which does not necessarily have to have a causal relationship with this treatment (the study medication).

An AE can therefore be any unfavourable and unintended sign (including an abnormal laboratory finding), symptom or disease temporally associated with the use of the study medication, whether or not considered related to the study medication.

8.1.2 Adverse Reaction (AR)

All untoward and unintended responses to a medicinal product related to any dose.

The phrase "responses to a medicinal product" means that a causal relationship between a study medication and an AE is at least a reasonable possibility, i.e., the relationship cannot be ruled out.

All cases judged by either the reporting medically qualified professional or the sponsor as having a reasonable suspected causal relationship to the study medication qualify as adverse reactions.

8.1.3 Serious Adverse Events and Reactions

A serious adverse event or reaction is any untoward medical occurrence that at any dose:

- Results in death,
- Is life-threatening,

NOTE: The term "life-threatening" in the definition of "serious" refers to an event in which the participant was at risk of death at the time of the event; it does not refer to an event that hypothetically might have caused death if it were more severe.

- Requires inpatient hospitalisation or prolongation of existing hospitalisation,
- Results in persistent or significant disability/incapacity, or
- Is a congenital anomaly/birth defect.
- Other important medical events

NOTE: Other events that may not result in death, are not life threatening, or do not require hospitalisation, may be considered a serious adverse event when, based upon appropriate medical judgement, the event may jeopardise the participant and may require medical or surgical intervention to prevent one of the outcomes listed above.

8.1.4 Suspected Unexpected Serious Adverse Reaction

A suspected unexpected serious adverse reaction (SUSAR) is an SAE, the nature or severity of which is unexpected (not consistent with the Investigator's brochure) and thought to be related to the study vaccine.

8.1.5 Severe Adverse Events

To ensure no confusion or misunderstanding of the difference between the terms "serious" and "severe", which are not synonymous, the following note of clarification is provided:
The term "severe" is often used to describe the intensity (severity) of a specific event (as in mild, moderate, or severe myocardial infarction); the event itself, however, may be of relatively minor medical significance (such as severe headache). This is not the same as "serious," which is based on participant/event outcome or action criteria usually associated with events that pose a threat to a participant's life or functioning. Seriousness (not severity) serves as a guide for defining regulatory reporting obligations.

8.2 Severity Grading of Adverse Events

Severity grading of all AEs will be assessed and determined by the principal investigator or designee. Criteria for grading the severity of adverse events (as mild, moderate, severe and very severe) are listed in the Adverse Event Grading Toxicity Table (Appendix B). The principle used to develop the grading criteria is shown in Table 4 and the same principle should be used in grading any events that have not been anticipated in the Adverse Event Grading Toxicity Table (Appendix B).

Table 4 *Severity Grading Criteria for Adverse Events*

Severity Grade	Description of Severity
Mild	Transient or mild discomfort. No prescribed therapy needed.
Moderate	Mild to moderate impact on activity. May be able to work full-time. May require minimal medical intervention.
Severe	Marked impact on activities of daily living. May be able to work

Severity Grade	Description of Severity
	part-time with some assistance. Requires medical intervention.
Very Severe	Extreme limitation in activity. Requires significant medical assistance.

8.3 Relationship to Investigational Medicinal Product

The likelihood that the IMP caused the (S) AE will be determined by an investigator who is a qualified physician. The investigator must sign and date the source document that supports the causality.

The criteria below are intended as a guideline to assist the investigator in determining causality (not all criteria must be present):

Not Related
(S)AEs that, after careful medical consideration, are clearly felt to be due to extraneous causes (e.g., disease, environment) and are unrelated to the IMPS.

Unlikely
(S)AEs that, after careful medical consideration, are felt with near certainty unlikely to be related to the IMP. In general, this category is applicable to (S)AEs that meet one of the following criteria:

- It does not follow a reasonable temporal (time) sequence from administration of the IMP .
- It may have been produced by the volunteer's clinical state, environmental or toxic factors, or other modes of therapy administered to the volunteer
- It does not follow a known pattern of response to the IMP

Possibly
(S)AEs that, after careful medical consideration, are felt unlikely to be related to the IMP although the possibility cannot be ruled out with certainty. In general, this category is applicable to (S)AEs that meet one of the following criteria:

- It follows a reasonable temporal (time) sequence from administration of the IMP
- It may have been produced by the volunteer's clinical state, environmental or toxic factors, or other modes of therapy administered to the volunteer
- It follows a known pattern of response to the IMP

Probably
(S)AEs that, after careful medical consideration, are felt with a high degree of certainty to be related to the IMP. In general, this category is applicable to (S)AEs that meets one of the following criteria:

- It follows a reasonable temporal (time) sequence from administration of the IMP
- It cannot be reasonably explained by the known characteristics of the volunteer's clinical state, environmental or toxic factors, or other modes of therapy administered to the volunteer
- It follows a known pattern or response to the IMP
- It reappears after repeat administration of IMP, if given

Definitely

(S)AEs that, after careful medical consideration, are felt with certainty to be related to the IMP.

8.4 Procedures for Recording Adverse Events

All adverse events including SAEs occurring from the time of first vaccination up to the end of the study will be recorded.
All AEs occurring during the study observed by the investigator or reported by the participant, whether or not attributed to study medication, will be recorded on the CRF.
The following information will be recorded:

- description, date of onset and end date, severity, assessment of relatedness to study medication, other suspect drug or device and action taken. Follow-up information should be provided as necessary.
- AEs considered related to the study medication as judged by a medically qualified investigator will be followed until resolution or the event is considered stable. All related AEs that result in a participant's withdrawal from the study or are present at the end of the study, should be followed up until a satisfactory resolution occurs.
- It will be left to the investigator's clinical judgment whether or not an AE is of sufficient severity to require the participant's removal from treatment. A participant may also voluntarily withdraw from treatment due to what he or she perceives as an intolerable AE. If either of these occurs, the participant must undergo an end of study assessment and be given appropriate care under medical supervision until symptoms cease or the condition becomes stable.
- The severity of events will be assessed on the following scale: 1 = mild, 2 = moderate, 3 = severe, 4 = very severe (see table 4).
- The relationship of AEs to the study medication will be assessed by a medically qualified investigator.
- Any pregnancy occurring during the clinical study and the outcome of the pregnancy, should be recorded and followed up for congenital abnormality or birth defect.

8.4.1 Reporting Serious Adverse Events

Serious Adverse Events should be reported to the DMC within 24 hours of the site personnel becoming aware of the event. SAEs should also be entered into the clinical trial database as soon as possible.

Notification to the DMC must be made by e-mail or fax, followed by telephone contact to confirm receipt, if appropriate.

The sponsor's SAE reporting form should be completed with all the available information at the time of reporting. The principal investigator or designee is required to produce a detailed written report, including the rationale for causality assessment, and follow-up the SAE to resolution (i.e., the volunteer recovers or dies, or the condition becomes chronic but relatively stable).

The principal investigator will notify GTAC/REC and MHRA of SUSARS according to their requirements. and timelines. In addition, the CI will submit a safety report to the MHRA, CTRG and GTAC/REC once a year throughout the trial, or on request.

8.4.2 Reporting Other Events

Pregnancy and HIV-1 infection should be reported to the DMC within 24 hours.

8.5 Clinical Management

Adverse events will be managed by the site personnel who will assess and treat the event as appropriate, including referral to an independent physician and/or department where necessary. Any adverse events will be followed up until resolution or stabilisation, where possible.

9 MANAGEMENT OF HIV-1 ISSUES DURING AND FOLLOWING THE STUDY

9.1 HIV-1 Testing

Only volunteers who are HIV-1-seronegative at screening will participate in the study.

The HIV-1 screening tests and routine post-vaccination tests will be performed as specified in the Schedule of Procedures (Appendix A).

If a volunteer during or after the study is found to be HIV-1-seropositive, a newly drawn blood specimen will be collected for confirmation.

If a volunteer appears to have developed HIV-1-specific antibodies as a result of vaccination and these cause reactivity on a standard HIV ELISA test, s/he will be followed up until the ELISA test becomes negative.

If a volunteer suspects s/he has been exposed to HIV during the study s/he would be recommended to contact the trial nurse/physician first. A risk assessment would be carried out by the trial nurse/physician and the volunteer would be offered an HIV-1 antibody test at the trial site, or referred to a GUM clinic if s/he prefers this option. Written information concerning tests performed and results will be provided upon request.

Volunteers who are found to be HIV-1-seropositive at screening and volunteers who acquire HIV infection during the study will receive counselling and referral for support and/or care.

9.2 Social Discrimination as a Result of Antibody Response to IMP

The aim is to minimise the possibility of social discrimination in volunteers (if any) who develop vaccine-induced HIV antibodies and therefore test positive on a routine HIV test.

The volunteer will be offered a volunteer card stating that he/she participated in a vaccine study giving a site contact number in case of medical emergency.

10 DISCONTINUATION OR POSTPONEMENT OF VACCINATIONS AND/OR WITHDRAWAL FROM THE STUDY

10.1 Discontinuation or Postponement of Vaccinations

Each participant has the right to withdraw from the study at any time. Any volunteer for whom further vaccinations are discontinued or postponed, or who is being considered for discontinuation or postponement of vaccinations will be discussed with the trial team. Volunteers will be discontinued or postponed from further vaccination for any of the following reasons:

- A disease, condition or an adverse event (including clinically significant abnormal laboratory values; see below) that develops, regardless of relationship to the IMP, if, in the opinion of the principal investigator or designee, further vaccinations would jeopardise the safety of the volunteer.
- Haematology

    - Haemoglobin < 10.0 g/dl
    - Absolute Neutrophil Count (ANC) ≤ 1000 /mm^3 (≤ 1 x 10^9 /l)
    - Absolute Lymphocyte Count (ALC) ≤ 600 /mm^3 (≤ 1 x 10^9 /l)
    - Platelets ≤ 100,000 /mm^3, ≥ 550,000 /mm^3 (≤ 90 /L, ≥ 550 /l)
- Biochemistry
    - Creatinine > 1.3 x ULN
    - AST > 2.5 x ULN
    - ALT > 2.5 x ULN
- Urinalysis (abnormal dipstick <u>confirmed</u> by microscopy)
    - Blood (not due to menses)
    - Leucocytes
- Confirmed intercurrent HIV-1 or HIV-2 Infection.
- Pregnancy.
- Receipt of live attenuated vaccine within 30 days.
- Receipt of blood transfusion or blood products during this study.
- Participating in another clinical trial of an IMP.
- A very severe local or systemic reactogenicity event judged to be possibly, probably or definitely related to vaccination.
- A very severe or serious adverse event judged to be possibly, probably or definitely related to vaccination.
- An adverse event which requires discontinuation of the IMP or results in inability to continue to comply with study procedures
- Loss to follow up
- The safety of the volunteer would be jeopardised in the opinion of the investigator or sponsor.
- Volunteer request.
- Investigator discretion.
- Significant protocol deviation.

10.1.1 Follow-Up after Discontinuation of Vaccinations

The reason for withdrawal will be recorded in the CRF.

Where possible, study procedures for Early Termination will be performed in the case of withdrawal (see Schedule of Procedures). If a volunteer develops a condition which may not necessarily be related to the IMP but which may cause the Investigators to recommend discontinuation of further vaccinations, the volunteer will continue to be followed up until any adverse event is judged to have resolved or stabilised.

Volunteers who discontinue vaccinations for one or more of the reasons above will remain in the study and all procedures, except vaccination, will be performed according to the Schedule of Procedures (Appendix A).

Any adverse event resulting in the discontinuation of a volunteer’s vaccinations will be followed up until, in the opinion of the principal investigator or designee, it has resolved or stabilised, where possible.

A volunteer who becomes pregnant will be followed to the completion or termination of the pregnancy. If the pregnancy continues to term, approximately 2-4 weeks after delivery, the health of the infant will be examined and reported to the sponsor.

Volunteers who have received the IMP and who acquire HIV-1 infection through sexual (or parenteral) exposure during the study will be followed as determined by the DMC.

10.2 Early Termination

A volunteer's participation in the study may be terminated for the following reasons:

- At any time if the volunteer wishes to do so, for any reason
- At the discretion of the investigator after consultation with sponsor
- If the sponsor decides to terminate or suspend the trial
- At the time of the withdrawal, provided the volunteer is willing, all the requested early termination visit procedures will be performed according to the Schedule of Procedures (Appendix A). Every effort will be made to determine the reason for withdrawal from the study.

11 DATA HANDLING

11.1 Data Collection and Record Keeping at the Trial Site

Data will be collected by the site personnel and recorded on the CRFs. Where information or data are first recorded on the CRF this will be the source document.

Other source documents include but are not limited to:

- Documentation of any existing conditions or past conditions relevant to eligibility
- Signed Informed Consent Forms
- Reported laboratory results

A file will be held at the trial site for each volunteer containing all the CRFs and source documents. All essential documents will be kept in a secure location and retained as required by ICH-GCP and applicable local requirements.

11.2 Data Entry at the Trial Site

Site personnel will enter the data collected at the site onto the database. All data will be recorded in source documents and no data will be entered directly onto the database. To provide for real time assessment of safety, data should be entered as soon as reasonably feasible (e.g., within one week) of a visit. Immunogenicity results will be transferred within two weeks of the assay being performed.

11.3 Data Analysis

The data analysis plan will be developed and agreed upon by the principal investigator prior to unblinding the study.

12 STATISTICAL CONSIDERATIONS

12.1 Statistical Power and Analysis

The primary goal of this study is to evaluate the safety and tolerability of candidate HIV-1 vaccines, MVA.HIVconsv, pSG2.HIVconsv DNA and ChAdV63.HIVconsv in HIV-1-seronegative individuals. The trial will enrol 32 subjects and it is not the remit of this study to recruit sufficient numbers of volunteers to be statistically confident about the result. However, the incidence of severe and very severe or serious adverse events will be used as a measure of safety of the IMP. Because of the small sample size the results will be primarily descriptive.

Summary statistics will be calculated; point and interval estimates of adverse event and immune response rates will be reported.

Immunogenicity will be evaluated in both validated quantitative assays and non-validated exploratory assays. The single-blind placebo-controlled design has been chosen to minimise bias in the reporting of immunological data. Statistical analysis will be applied to evaluate changes in HIV-1 epitope-specific CD8+ T cell responses detected in IFN-γ ELISPOT assays before and after vaccination. Sub-group analysis will be performed to discern a dose or regimen effect. Because of the small sample size the results will be primarily descriptive.

This study is not powered to detect a significant difference between vaccinees and controls in the magnitude or breadth of immune response to the immunogen. However, it may be possible to discern trends, which could inform the design of future studies.

12.1.1 Safety and Tolerability

The incidence of severe and very severe adverse events will be used as one measure of the safety of the IMP. Severe and very severe reactions that may be temporarily incapacitating (for example, loss or cancellation of work or social activities), which could make a vaccine impractical for large scale use if they occur in more than a small proportion of cases, will be assessed. All adverse events, their severity grading and relationship to the IMP (as judged by the investigator) will be reported, whether or not they qualify as SAEs.

12.1.2 Immunogenicity

Cellular immune responses will be analysed using binomial methods to examine for the presence or absence of HIV-1 epitope-specific T cell responses quantified by IFN-γ ELISPOT and intracellular cytokine assay. Assays will be performed in a similar fashion in all volunteers. Response rates will be determined and compared between the volunteers who receive the different regimens. Separately, the volunteers receiving placebo will be compared with the vaccine groups. Because of the small sample size (and therefore lack of statistical power to detect small differences) and multiple epitopes, the results will be primarily descriptive.
The procedure for handling any missing, unused or spurious safety or immunogenicity data will be described in the data analysis plan.

13 QUALITY CONTROL AND QUALITY ASSURANCE

Regular monitoring will be performed in compliance with ICH-GCP in accordance with the study specific monitoring plan. Following written SOPS, the trial monitors will verify that the study is conducted, recorded and reported in compliance with the protocol, ICH-GCP and applicable regulatory requirements. An independent audit of the study may be performed, if required by the sponsor.

The chief investigator (by signing the protocol) and the volunteers (by giving informed consent) permit study-related monitoring, audits, IRB/IEC review (s) and regulatory inspection (s) and direct access to source documents. Such information will be treated as strictly confidential and will under no circumstances be made publicly available.

14 DATA AND BIOLOGICAL MATERIAL

All data and all biological material collected through the study shall be the property of the University of Oxford.

All CRFs and laboratory reports will be reviewed by the clinical team, who will ensure that they are accurate and complete.

CRFs and other study documents will be stored in a secure location separate from study participant identification information to ensure confidentiality. On all study-specific documents, other than the signed consent, the participant will be referred to by the study participant number/code.

A copy of the following information will be available in the participant's research file:

- Written informed consent, including documentation of the version and date of the Participant Information Leaflet to which the consent refers
- Dates of visits, including dates of vaccinations

Direct access to all data and documentation associated with the trial will be granted to authorised representatives from the sponsor, host institution and the regulatory authorities to permit trial-related monitoring, audits and inspections.

Study data may be transcribed from the CRFs onto an electronic data entry system. The security and confidentiality of the data will be maintained in accordance with the Data Protection Act 1998 and Standard Operating Procedures which include: restricted access through password protection and retention of electronic data entry systems on secure premises with access restricted to authorised personnel; removal of all participant identifiers; identification of study participants by study number / code only.

15 ADMINISTRATIVE STRUCTURE

The principal investigator will be responsible for all aspects of the study at the trial site.

15.1 Data Monitoring Committee (DMC)

The Data Monitoring Committee (DMC) will oversee the progress and safety of the study. The DMC will consist of specialists who are not involved in the study. Investigators responsible for the clinical care of volunteers or representative of the sponsor may not be members of the DMC. However, the DMC may invite the principal investigator or designee and a sponsor representative to an open session of the meeting to provide information on study conduct, present data or to respond to questions.

The review of study data by the DMC will take place regularly or may be specifically requested.

15.2 Content of Interim Review

After completion of stage 2 and stage 3, an interim review will be prepared by the principal investigator for the DMC and will contain any of the following data depending on availability:

- All severe or very severe clinical adverse event and reactogenicity events judged by the principal investigator to be possibly, probably or definitely related to the IMP

- All severe or very severe laboratory adverse events judged by the principal investigator to be possibly, probably, or definitely related to IMPs

- All Serious Adverse Events, independent of relationship to the IMP

- T cell responses following the final vaccination compared to responses before the first vaccination and before the final vaccination

- HIV-1 specific T-cell responses quantified by ELISPOT

- HIV-1 specific T-cell responses quantified by intracellular cytokine assay

- HIV-1 specific T-cell responses quantified by restimulation assay measuring tetramer staining

- HIV-1 specific T-cell responses quantified by restimulation assay measuring proliferation

- HIV-1 specific T-cell responses quantified by restimulation assay measuring cytolysis

- HIV-1 specific T-cell responses quantified by cytokine production assay

- Control of HIV-1 replication *in vitro*

15.3 Indications for Discontinuation of Vaccinations in all Volunteers

If two or more volunteers develop any clinically similar SAE judged definitely, probably or possibly related to the IMP (SAR), the principal investigator and the sponsor will request a review by the DMC. The study will be suspended pending a review of all safety data by the DMC.

Following this review the DMC will make a recommendation to the sponsor and the principal investigator regarding the continuation of the study. Unless the study is permanently discontinued, the volunteers will not be made aware of any unblinded results.

16 INDEMNITY

Indemnity and/or compensation for negligent harm arising specifically from an accidental injury for which the University is legally liable as the Research Sponsor will be covered by the University of Oxford.

17 PUBLICATION

A preliminary manuscript describing safety and immunogenicity responses in this study will be prepared promptly after the data analyses are available. The investigators will be involved in reviewing all drafts of the manuscripts, abstracts, press releases and any other publications arising from the trial and retain final editorial control. The authors shall acknowledge that the study was carried out with support from the Medical Research Council, UK.

The authors will be determined subject to the generally accepted criteria of contributions to the design, work, analysis and writing of the trial. All contributors who do not meet the criteria for authorship will be acknowledged.

18 REGULATORY, ETHICAL AND LEGAL OBLIGATIONS

This principal investigator will ensure that the trial is conducted in compliance with the protocol, Standard Operating Procedures that meet the guidelines laid down by the International Conference on Harmonisation for Good Clinical Practice in clinical trials, i and applicable regulatory requirement (s).

In addition to ethical and regulatory approvals, all other required approvals (e.g., GTAC) will be obtained before shipment of IMPS and recruitment of volunteers.

18.1 Declaration of Helsinki

The Investigator will ensure that this study is conducted in accordance with the principles of the current revision of the Declaration of Helsinki (last amended October 2008).

18.2 ICH Guidelines for Good Clinical Practice

The Chief Investigator will ensure that this study is conducted in full conformity with relevant regulations and with the ICH Guidelines for Good Clinical Practice (CPMP/ICH/135/95) July 1996.

18.3 Participant Confidentiality

The trial staff will ensure that the participants' anonymity is maintained. Only initials and a participants ID number on the CRF and any electronic database will identify the participants. All documents will be stored securely and will be accessible only to trial staff and authorised personnel. The study will comply with the Data Protection Act, which requires data to be anonymised as soon as it is practical to do so.

19 DATA PROTECTION

All personnel involved in the study will observe or work in compliance with the Data Protection Act 1998.

20 REFERENCES

1. B. D. Walker and D. R. Burton, Toward an AIDS vaccine. Science, 320, 760-764 (2008).
2. The Global HIV/AIDS Vaccine Enterprise: scientific strategic plan. PLoS Med, 2, e25 (2005).
3. N. L. Letvin, J. R. Mascola, Y. Sun, D. A. Gorgone, A. P. Buzby, L. Xu, Z. Y. Yang, B. Chakrabarti, S. S. Rao, J. E. Schmitz, D. C. Montefiori, B. R. Barker, F. L. Bookstein, G. J. Nabel, Preserved CD4+ central memory T cells and survival in vaccinated SIV-challenged monkeys. Science, 312, 1530-1533 (2006).
4. M. M. Addo, X. G. Yu, A. Rathod, D. Cohen, R. L. Eldridge, D. Strick, M. N. Johnston, C. Corcoran, A. G. Wurcel, C. A. Fitzpatrick, M. E. Feeney, W. R. Rodriguez, N. Basgoz, R. Draenert, D. R. Stone, C. Brander, P. J. Goulder, E. S. Rosenberg, M. Altfeld, W. B. D., Comprehensive epitope analysis of human immunodeficiency virus type 1 (HIV-1)-specific T-cell responses directed against the entire expressed HIV-1 genome demonstrate broadly directed responses, but no correlation to viral load. J Virol, 77, 2081-2092 (2003).
5. C. A. Baalen, C. Guillon, M. v. M. Baalen, E. J. Verschuren, P. H. Boers, A. D. Osterhaus, R. A. Gruters, Impact of antigen expression kinetics on the effectiveness of HIV-specific cytotoxic T lymphocytes. Eur J Immunol, 32, 2644-2652 (2002).
6. M. R. Betts, D. R. Ambrozak, D. C. Douek, S. Bonhoeffer, J. M. Brenchley, J. P. Casazza, R. A. Koup, L. J. Picker, Analysis of total human immunodeficiency virus (HIV)-specific CD4(+) and CD8(+) T-cell responses: relationship to viral load in untreated HIV infection. J Virol, 75, 11983-11991 (2001).
7. S. L. Rowland-Jones, S. Pinheiro, R. Kaul, P. Hansasuta, G. Gillespie, T. Dong, F. A. Plummer, J. B. Bwayo, S. Fidler, J. Weber, A. McMichael, V. Appay, How important is the 'quality' of the cytotoxic T lymphocyte (CTL) response in protection against HIV infection? Immunol Lett, 79, 15-20 (2001).
8. D. Zhang, P. Shankar, Z. Xu, B. Harnisch, G. Chen, C. Lange, S. J. Lee, H. Valdez, M. M. Lederman, J. Lieberman, Most antiviral CD8 T cells during chronic viral infection do not express high levels of perforin and are not directly cytotoxic. Blood, 101, 226-235 (2003).
9. I. Honeyborne, A. Prendergast, F. Pereyra, A. Leslie, H. Crawford, R. Payne, S. Reddy, K. Bishop, E. Moodley, K. Nair, M. van der Stok, N. McCarthy, C. M. Rousseau, M. Addo, J. I. Mullins, C. Brander, P. Kiepiela, B. D. Walker, P. J. Goulder, Control of human immunodeficiency virus type 1 is associated with HLA-B*13 and targeting of multiple gag-specific CD8+ T-cell epitopes. J Virol, 81, 3667-3672 (2007).
10. P. Kiepiela, K. Ngumbela, C. Thobakgale, D. Ramduth, I. Honeyborne, E. Moodley, S. Reddy, C. de Pierres, Z. Mncube, N. Mkhwanazi, K. Bishop, M. van der Stok, K. Nair, N. Khan, H. Crawford, R. Payne, A. Leslie, J. Prado, A. Prendergast, J. Frater, N. McCarthy, C. Brander, G. H. Learn, D. Nickle, C. Rousseau, H. Coovadia, J. I. Mullins, D. Heckerman, B. D. Walker, P. Goulder, CD8+ T-cell responses to different HIV proteins have discordant associations with viral load. Nat Med, 13, 46-53 (2007).
11. M. Rolland, D. Heckerman, W. Deng, C. M. Rousseau, H. Coovadia, K. Bishop, P. J. Goulder, B. D. Walker, C. Brander, J. I. Mullins, Broad and Gag-Biased HIV-1 Epitope Repertoires Are Associated with Lower Viral Loads. PLoS ONE, 3, e1424 (2008).
12. N. Goonetilleke, M. K. Liu, J. F. Salazar-Gonzalez, G. Ferrari, E. Giorgi, V. V. Ganusov, B. F. Keele, G. H. Learn, E. L. Turnbull, M. G. Salazar, K. J. Weinhold, S.

Moore, N. Letvin, B. F. Haynes, M. S. Cohen, P. Hraber, T. Bhattacharya, P. Borrow, A. S. Perelson, B. H. Hahn, G. M. Shaw, B. T. Korber, A. J. McMichael, The first T cell response to transmitted/founder virus contributes to the control of acute viremia in HIV-1 infection. J Exp Med, 206, 1253-1272 (2009).

13. B. F. Keele, E. E. Giorgi, J. F. Salazar-Gonzalez, J. M. Decker, K. T. Pham, M. G. Salazar, C. Sun, T. Grayson, S. Wang, H. Li, X. Wei, C. Jiang, J. L. Kirchherr, F. Gao, J. A. Anderson, L. H. Ping, R. Swanstrom, G. D. Tomaras, W. A. Blattner, P. A. Goepfert, J. M. Kilby, M. S. Saag, E. L. Delwart, M. P. Busch, M. S. Cohen, D. C. Montefiori, B. F. Haynes, B. Gaschen, G. S. Athreya, H. Y. Lee, N. Wood, C. Seoighe, A. S. Perelson, T. Bhattacharya, B. T. Korber, B. H. Hahn, G. M. Shaw, Identification and characterization of transmitted and early founder virus envelopes in primary HIV-1 infection. Proc Natl Acad Sci U S A, 105, 7552-7557 (2008).

14. J. F. Salazar-Gonzalez, M. G. Salazar, B. F. Keele, G. H. Learn, E. E. Giorgi, H. Li, J. M. Decker, S. Wang, J. Baalwa, M. H. Kraus, N. F. Parrish, K. S. Shaw, M. B. Guffey, K. J. Bar, K. L. Davis, C. Ochsenbauer-Jambor, J. C. Kappes, M. S. Saag, M. S. Cohen, J. Mulenga, C. A. Derdeyn, S. Allen, E. Hunter, M. Markowitz, P. Hraber, A. S. Perelson, T. Bhattacharya, B. F. Haynes, B. T. Korber, B. H. Hahn, G. M. Shaw, Genetic identity, biological phenotype, and evolutionary pathways of transmitted/founder viruses in acute and early HIV-1 infection. J Exp Med, 206, 1273-1289 (2009).

15. R. A. Gruters, C. A. van Baalen, A. D. Osterhaus, The advantage of early recognition of HIV-infected cells by cytotoxic T-lymphocytes. Vaccine, 20, 2011-2015 (2002).

16. J. B. Sacha, C. Chung, E. G. Rakasz, S. P. Spencer, A. K. Jonas, A. T. Bean, W. Lee, B. J. Burwitz, J. J. Stephany, J. T. Loffredo, D. B. Allison, S. Adnan, A. Hoji, N. A. Wilson, T. C. Friedrich, J. D. Lifson, O. O. Yang, D. I. Watkins, Gag-specific CD8+ T lymphocytes recognize infected cells before AIDS-virus integration and viral protein expression. J Immunol, 178, 2746-2754 (2007).

17. J. B. Sacha, C. Chung, J. Reed, A. K. Jonas, A. T. Bean, S. P. Spencer, W. Lee, L. Vojnov, R. Rudersdorf, T. C. Friedrich, N. A. Wilson, J. D. Lifson, D. I. Watkins, Pol-specific CD8+ T cells recognize simian immunodeficiency virus-infected cells prior to Nef-mediated major histocompatibility complex class I downregulation. J Virol, 81, 11703-11712 (2007).

18. J. B. Sacha, J. P. Giraldo-Vela, M. B. Buechler, M. A. Martins, N. J. Maness, C. Chung, L. T. Wallace, E. J. Leon, T. C. Friedrich, N. A. Wilson, A. Hiraoka, D. I. Watkins, Gag- and Nef-specific CD4+ T cells recognize and inhibit SIV replication in infected macrophages early after infection. Proc Natl Acad Sci U S A, 106, 9791-9796 (2009).

19. J. Liu, K. L. O'Brien, D. M. Lynch, N. L. Simmons, A. La Porte, A. M. Riggs, P. Abbink, R. T. Coffey, L. E. Grandpre, M. S. Seaman, G. Landucci, D. N. Forthal, D. C. Montefiori, A. Carville, K. G. Mansfield, M. J. Havenga, M. G. Pau, J. Goudsmit, D. H. Barouch, Immune control of an SIV challenge by a T-cell-based vaccine in rhesus monkeys. Nature, 457, 87-91 (2008).

20. S. Letourneau, E.-J. Im, T. Mashishi, C. Brereton, A. Bridgeman, H. Yang, L. Dorrell, T. Dong, B. Korber, A. J. McMichael, T. Hanke, Design and pre-clinical evaluation of a universal HIV-1 vaccine. PLoS ONE, 2, e984 (2007).

21. M. Rolland, D. C. Nickle, J. I. Mullins, HIV-1 group M conserved elements vaccine. PLoS Pathog, 3, e157 (2007).

22. P. Borrow, H. Lewicki, B. E. Hahn, G. M. Shaw, M. B. Oldstone, Virus-specific CD8+ CTL activity associated with control of viremia in primary HIV-1 infection. J. Virol., 68, 6103-6110 (1994).

23. D. R. Casimiro, F. Wang, W. A. Schleif, X. Liang, Z. Q. Zhang, T. W. Tobery, M. E. Davies, A. B. McDermott, D. H. O'Connor, A. Fridman, A. Bagchi, L. G. Tussey, A. J. Bett, A. C. Finnefrock, T. M. Fu, A. Tang, K. A. Wilson, M. Chen, H. C. Perry, G. J. Heidecker, D. C. Freed, A. Carella, K. S. Punt, K. J. Sykes, L. Huang, V. I. Ausensi, M. Bachinsky, U. Sadasivan-Nair, D. I. Watkins, E. A. Emini, J. W. Shiver, Attenuation of simian immunodeficiency virus SIVmac239 infection by prophylactic immunization with dna and recombinant adenoviral vaccine vectors expressing Gag. J Virol, 79, 15547-15555 (2005).
24. J. Fellay, K. V. Shianna, D. Ge, S. Colombo, B. Ledergerber, M. Weale, K. Zhang, C. Gumbs, A. Castagna, A. Cossarizza, A. Cozzi-Lepri, A. De Luca, P. Easterbrook, P. Francioli, S. Mallal, J. Martinez-Picado, J. M. Miro, N. Obel, J. P. Smith, J. Wyniger, P. Descombes, S. E. Antonarakis, N. L. Letvin, A. J. McMichael, B. F. Haynes, A. Telenti, D. B. Goldstein, A whole-genome association study of major determinants for host control of HIV-1. Science, 317, 944-947 (2007).
25. M. Fujiwara and M. Takiguchi, HIV-1-specific CTLs effectively suppress replication of HIV-1 in HIV-1-infected macrophages. Blood, 109, 4832-4838 (2007).
26. X. Jin, D. E. Bauer, S. E. Tuttleton, S. Lewin, A. Gettie, J. Blanchard, C. E. Irwin, J. T. Safrit, J. Mittler, L. Weinberger, L. G. Kostrikis, L. Zhang, A. S. Perelson, D. D. Ho, Dramatic rise in plasma viremia after CD8(+) T cell depletion in simian immunodeficiency virus-infected macaques. J. Exp. Med., 189, 991-998 (1999).
27. P. Klenerman, Y. Wu, R. Phillips, HIV: current opinion in escapology. Curr Opin Microbiol, 5, 408-413 (2002).
28. R. A. Koup, J. T. Safrit, Y. Cao, C. A. Andrews, G. McLoed, W. Borkowsky, C. Farthing, D. D. Ho, Temporal association of cellular immune responses with the initial control of viremia in primary human immunodeficiency virus type 1 syndrome. J. Virol., 68, 4650-4655 (1994).
29. T. Matano, R. Shibata, C. Siemon, M. Connors, H. C. Lane, M. A. Martin, Administration of an anti-CD8 monoclonal antibody interferes with the clearance of chimeric simian/human immunodeficiency virus during primary infections of rhesus macaques. J Virol, 72, 164-169. (1998).
30. S. J. O'Brien, X. Gao, M. Carrington, HLA and AIDS: a cautionary tale. Trends Mol Med, 7, 379-381 (2001).
31. J. E. Schmitz, M. J. Kuroda, V. G. Sasseville, M. A. Simon, M. A. Lifton, P. Racz, K. Tenner-Racz, M. Dalesandro, B. J. Scallon, J. Ghrayeb, M. A. Forman, D. C. Montefiori, E. P. Rieber, N. L. Letvin, K. A. Reinmann, Control of viremia in simian immunodeficiency virus infection by CD8+ lymphocytes. Science, 283, 857-860 (1999).
32. H. Yang, T. Dong, E. Turnbull, S. Ranasinghe, B. Ondondo, N. Goonetilleke, N. Winstone, K. di Gleria, P. Bowness, C. Conlon, P. Borrow, T. Hanke, A. McMichael, L. Dorrell, Broad TCR usage in functional HIV-1-specific CD8+ T cell expansions driven by vaccination during highly active antiretroviral therapy. J Immunol, 179, 597-606 (2007).
33. V. Appay, D. C. Douek, D. A. Price, CD8+ T cell efficacy in vaccination and disease. Nat Med, 14, 623-628 (2008).
34. T. Hanke, STEP trial and HIV-1 vaccines inducing T-cell responses. Expert Rev Vaccines, 7, 303-309 (2008).
35. D. I. Watkins, D. R. Burton, E. G. Kallas, J. P. Moore, W. C. Koff, Nonhuman primate models and the failure of the Merck HIV-1 vaccine in humans. Nat Med, 14, 617-621 (2008).
36. J. R. Almeida, D. A. Price, L. Papagno, Z. A. Arkoub, D. Sauce, E. Bornstein, T. E. Asher, A. Samri, A. Schnuriger, I. Theodorou, D. Costagliola, C. Rouzioux, H. Agut,

A. G. Marcelin, D. Douek, B. Autran, V. Appay, Superior control of HIV-1 replication by CD8+ T cells is reflected by their avidity, polyfunctionality, and clonal turnover. J Exp Med, 204, 2473-2485 (2007).

37. J. R. Almeida, D. Sauce, D. A. Price, L. Papagno, S. Y. Shin, A. Moris, M. Larsen, G. Pancino, D. C. Douek, B. Autran, A. Saez-Cirion, V. Appay, Antigen sensitivity is a major determinant of CD8+ T-cell polyfunctionality and HIV-suppressive activity. Blood, 113, 6351-6360 (2009).
38. M. S. Bennett, H. L. Ng, M. Dagarag, A. Ali, O. O. Yang, Epitope-dependent avidity thresholds for cytotoxic T-lymphocyte clearance of virus-infected cells. J Virol, 81, 4973-4980 (2007).
39. M. Derby, M. Alexander-Miller, R. Tse, J. Berzofsky, High-avidity CTL exploit two complementary mechanisms to provide better protection against viral infection than low-avidity CTL. J Immunol, 166, 1690-1697 (2001).
40. I. Messaoudi, J. A. Guevara Patino, R. Dyall, J. LeMaoult, J. Nikolich-Zugich, Direct link between mhc polymorphism, T cell avidity, and diversity in immune defense. Science, 298, 1797-1800 (2002).
41. D. A. Price, J. M. Brenchley, L. E. Ruff, M. R. Betts, B. J. Hill, M. Roederer, R. A. Koup, S. A. Migueles, E. Gostick, L. Wooldridge, A. K. Sewell, M. Connors, D. C. Douek, Avidity for antigen shapes clonal dominance in CD8+ T cell populations specific for persistent DNA viruses. J Exp Med, 202, 1349-1361 (2005).
42. S. A. Migueles, A. C. Laborico, W. L. Shupert, M. S. Sabbaghian, R. Rabin, C. W. Hallahan, D. Van Baarle, S. Kostense, F. Miedema, M. McLaughlin, L. Ehler, J. Metcalf, S. Liu, M. Connors, HIV-specific CD8+ T cell proliferation is coupled to perforin expression and is maintained in nonprogressors. Nat Immunol, 3, 1061-1068 (2002).
43. M. R. Betts, M. C. Nason, S. M. West, S. C. De Rosa, S. A. Migueles, J. Abraham, M. M. Lederman, J. M. Benito, P. A. Goepfert, M. Connors, M. Roederer, R. A. Koup, HIV nonprogressors preferentially maintain highly functional HIV-specific CD8+ T cells. Blood, 107, 4781-4789 (2006).
44. D. A. Price, T. E. Asher, N. A. Wilson, M. C. Nason, J. M. Brenchley, I. S. Metzler, V. Venturi, E. Gostick, P. K. Chattopadhyay, M. Roederer, M. P. Davenport, D. I. Watkins, D. C. Douek, Public clonotype usage identifies protective Gag-specific CD8+ T cell responses in SIV infection. J Exp Med, 206, 923-936 (2009).
45. A. Harari, F. Vallelian, G. Pantaleo, Phenotypic heterogeneity of antigen-specific CD4 T cells under different conditions of antigen persistence and antigen load. Eur J Immunol, 34, 3525-3533 (2004).
46. J. W. Hodge, M. Chakraborty, C. Kudo-Saito, C. T. Garnett, J. Schlom, Multiple costimulatory modalities enhance CTL avidity. J Immunol, 174, 5994-6004 (2005).
47. U. Wille-Reece, B. J. Flynn, K. Lore, R. A. Koup, A. P. Miles, A. Saul, R. M. Kedl, J. J. Mattapallil, W. R. Weiss, M. Roederer, R. A. Seder, Toll-like receptor agonists influence the magnitude and quality of memory T cell responses after prime-boost immunization in nonhuman primates. J Exp Med, 203, 1249-1258 (2006).
48. Q. Li, P. J. Skinner, S. J. Ha, L. Duan, T. L. Mattila, A. Hage, C. White, D. L. Barber, L. O'Mara, P. J. Southern, C. S. Reilly, J. V. Carlis, C. J. Miller, R. Ahmed, A. T. Haase, Visualizing antigen-specific and infected cells in situ predicts outcomes in early viral infection. Science, 323, 1726-1729 (2009).
49. A. J. Hessell, L. Hangartner, M. Hunter, C. E. Havenith, F. J. Beurskens, J. M. Bakker, C. M. Lanigan, G. Landucci, D. N. Forthal, P. W. Parren, P. A. Marx, D. R. Burton, Fc receptor but not complement binding is important in antibody protection against HIV. Nature, 449, 101-104 (2007).

50. S. Rerks-Ngarm, P. Pitisuttithum, S. Nitayaphan, J. Kaewkungwal, J. Chiu, R. Paris, N. Premsri, C. Namwat, M. de Souza, E. Adams, M. Benenson, S. Gurunathan, J. Tartaglia, J. G. McNeil, D. P. Francis, D. Stablein, D. L. Birx, S. Chunsuttiwat, C. Khamboonruang, P. Thongcharoen, M. L. Robb, N. L. Michael, P. Kunasol, J. H. Kim, Vaccination with ALVAC and AIDSVAX to Prevent HIV-1 Infection in Thailand. N Engl J Med, (2009).
51. C. Williamson, L. Morris, M. F. Maughan, L. H. Ping, S. A. Dryga, R. Thomas, E. A. Reap, T. Cilliers, J. van Harmelen, A. Pascual, G. Ramjee, G. Gray, R. Johnston, S. A. Karim, R. Swanstrom, Characterization and selection of HIV-1 subtype C isolates for use in vaccine development. AIDS Res Hum Retroviruses, 19, 133-144 (2003).
52. R. R. Amara, S. Sharma, M. Patel, J. M. Smith, L. Chennareddi, J. G. Herndon, H. L. Robinson, Studies on the cross-clade and cross-species conservation of HIV-1 Gag-specific CD8 and CD4 T cell responses elicited by a clade B DNA/MVA vaccine in macaques. Virology, 334, 124-133 (2005).
53. M. R. Betts, J. Krowka, C. Santamaria, K. Balsamo, F. Gao, G. Mulundu, C. Luo, N. N'Gandu, H. Sheppard, B. H. Hahn, S. Allen, J. A. Frelinger, Cross-clade human immunodeficiency virus (HIV)-specific cytotoxic T-lymphocyte responses in HIV-infected Zambians. J. Virol., 71, 8908-8911 (1997).
54. F. Buseyne, M. L. Chaix, B. Fleury, O. Manigard, M. Burgard, S. Blanche, C. Rouzioux, Y. Riviere, Cross-clade-specific cytotoxic T lymphocytes in HIV-1-infected children. Virology, 250, 316-324 (1998).
55. H. Cao, P. Kanki, J. L. Sankale, A. Dieng-Sarr, G. P. Mazzara, S. A. Kalams, B. Korber, S. Mboup, B. D. Walker, Cytotoxic T lymphocyte cross-reactivity among different human immunodeficiency virus type 1 clades: implications for vaccine development. J. Virol., 71, 8615-8623 (1997).
56. G. Ferrari, W. Humphrey, M. J. McElrath, J. L. Excler, A. M. Duliege, M. L. Clements, L. C. Corey, D. P. Bolognesi, K. J. Weinhold, Clade B-based HIV-1 vaccines elicit cross-clade cytotoxic T lymphocyte reactivities in uninfected volunteers. Proc. Natl. Acad. Sci. USA, 94, 1396-1401 (1997).
57. S. R. Burrows, S. L. Silins, D. J. Moss, R. Khanna, I. S. Misko, V. P. Argaet, T cell receptor repertoire for a viral epitope in humans is diversified by tolerance to a background major histocompatibility complex antigen. J Exp Med, 182, 1703-1715 (1995).
58. L. Dorrell, T. Dong, G. S. Ogg, S. McAdam, o. Anzala, T. Rostron, C. Conlon, A. J. McMichael, S. L. Rowland-Jones, Distinct recognition of clade A HIV-1 epitopes by cytotoxic T lymphocytes generated from donors infected in Africa. J. Virol., 73, 1708-1714 (1999).
59. L. Dorrell, B. E. Willcox, E. Y. Jones, G. Gillespie, H. Njai, S. Sabally, A. Jaye, K. DeGleria, T. Rostron, E. Lepin, A. McMichael, H. Whittle, S. Rowland-Jones, Cytotoxic T lymphocytes recognize structurally diverse, clade-specific and cross-reactive peptides in human immunodeficiency virus type-1 gag through HLA-B53. Eur J Immunol, 31, 1747-1756 (2001).
60. F. Gotch, A. McMichael, J. Rothbard, Recognition of influenza A matrix protein by HLA-A2-restricted cytotoxic T lymphocytes. Use of analogues to orientate the matrix peptide in the HLA-A2 binding site. J Exp Med, 168, 2045-2057 (1988).
61. S. Hausmann, W. E. Biddison, K. J. Smith, Y. H. Ding, D. N. Garboczi, U. Utz, D. C. Wiley, K. W. Wucherpfennig, Peptide recognition by two HLA-A2/Tax11-19-specific T cell clones in relationship to their MHC/peptide/TCR crystal structures. J Immunol, 162, 5389-5397 (1999).
62. N. Larke, E.-J. Im, R. Wagner, C. Williamson, A.-L. Williamson, A. J. McMichael, T. Hanke, Combined single-clade candidate HIV-1 vaccines induce T cell responses

limited by multiple forms of *in vivo* immune interference. Eur J Immunol, 37, 566-577 (2007).
63. J. K. Lee, G. Stewart-Jones, T. Dong, K. Harlos, K. Di Gleria, L. Dorrell, D. C. Douek, P. A. van der Merwe, E. Y. Jones, A. J. McMichael, T cell cross-reactivity and conformational changes during TCR engagement. J Exp Med, 200, 1455-1466 (2004).
64. P. J. Goulder and D. I. Watkins, HIV and SIV CTL escape: implications for vaccine design. Nat Rev Immunol, 4, 630-640 (2004).
65. A. J. McMichael, HIV vaccines. Annu Rev Immunol, 24, 227-255 (2006).
66. B. D. Walker and P. J. Goulder, AIDS. Escape from the immune system. Nature, 407, 313-314 (2000).
67. A. McMichael, M. Mwau, T. Hanke, HIV T cell vaccines, the importance of clades. Vaccine, 20, 1918-1921 (2002).
68. W. M. Blay, S. Gnanakaran, B. Foley, N. A. Doria-Rose, B. T. Korber, N. L. Haigwood, Consistent patterns of change during the divergence of human immunodeficiency virus type 1 envelope from that of the inoculated virus in simian/human immunodeficiency virus-infected macaques. J Virol, 80, 999-1014 (2006).
69. B. Gaschen, J. Taylor, K. Yusim, B. Foley, F. Gao, D. Lang, V. Novitsky, B. Haynes, B. H. Hahn, T. Bhattacharya, B. Korber, Diversity considerations in HIV-1 vaccine selection. Science, 296, 2354-2360 (2002).
70. F. Gao, B. T. Korber, E. Weaver, H. X. Liao, B. H. Hahn, B. F. Haynes, Centralized immunogens as a vaccine strategy to overcome HIV-1 diversity. Expert Rev Vaccines, 3, S161-168 (2004).
71. F. Gao, E. A. Weaver, Z. Lu, Y. Li, H. X. Liao, B. Ma, S. M. Alam, R. M. Scearce, L. L. Sutherland, J. S. Yu, J. M. Decker, G. M. Shaw, D. C. Montefiori, B. T. Korber, B. H. Hahn, B. F. Haynes, Antigenicity and immunogenicity of a synthetic human immunodeficiency virus type 1 group m consensus envelope glycoprotein. J Virol, 79, 1154-1163 (2005).
72. D. L. Kothe, J. M. Decker, Y. Li, Z. Weng, F. Bibollet-Ruche, K. P. Zammit, M. G. Salazar, Y. Chen, J. F. Salazar-Gonzalez, Z. Moldoveanu, J. Mestecky, F. Gao, B. F. Haynes, G. M. Shaw, M. Muldoon, B. T. Korber, B. H. Hahn, Antigenicity and immunogenicity of HIV-1 consensus subtype B envelope glycoproteins. Virology, 360, 218-234 (2007).
73. D. L. Kothe, Y. Li, J. M. Decker, F. Bibollet-Ruche, K. P. Zammit, M. G. Salazar, Y. Chen, Z. Weng, E. A. Weaver, F. Gao, B. F. Haynes, G. M. Shaw, B. T. Korber, B. H. Hahn, Ancestral and consensus envelope immunogens for HIV-1 subtype C. Virology, 352, 438-449 (2006).
74. E. A. Weaver, Z. Lu, Z. T. Camacho, F. Moukdar, H. X. Liao, B. J. Ma, M. Muldoon, J. Theiler, G. J. Nabel, N. L. Letvin, B. T. Korber, B. H. Hahn, B. F. Haynes, F. Gao, Cross-subtype T-cell immune responses induced by a human immunodeficiency virus type 1 group m consensus env immunogen. J Virol, 80, 6745-6756 (2006).
75. L. Dorrell, H. Yang, B. Ondondo, T. Dong, K. di Gleria, A. Suttill, C. Conlon, D. Brown, P. Williams, P. Bowness, N. Goonetilleke, T. Rostron, S. Rowland-Jones, T. Hanke, A. J. McMichael, Expansion and diversification of HIV-1-specific T cells following immunisation of HIV-1-infected individuals with a recombinant modified vaccinia virus Ankara / HIV-1 gag vaccine. J Virol, 80, 4705-4716 (2006).
76. N. Goonetilleke, S. Moore, L. Dally, N. Winstone, N. Mahmoud, I. Cebere, S. Pinheiro, G. Gillespie, D. Brown, V. Loach, J. Roberts, A. Guimaraes-Walker, P. Hayes, K. Loughran, C. Smith, P. Fast, L. Dorrell, T. Hanke, A. McMichael, Prime-boost vaccination with recombinant DNA and MVA expressing HIV-1 Clade A gag

and immunodominant CTL epitopes induces multi-functional HIV-1-specific T cells in healthy subjects. J Virol, 80, 4717-4728 (2006).
77. J. A. Slyker, B. L. Lohman, D. A. Mbori-Ngacha, M. Reilly, E. G. Wee, T. Dong, A. J. McMichael, S. L. Rowland-Jones, T. Hanke, G. John-Stewart, Modified vaccinia Ankara expressing HIVA antigen stimulates HIV-1-specific CD8 T cells in ELISpot assays of HIV-1 exposed infants. Vaccine, 23, 4711-4719 (2005).
78. M. Altfeld, M. M. Addo, R. Shankarappa, P. K. Lee, T. M. Allen, X. G. Yu, A. Rathod, J. Harlow, K. O'Sullivan, M. N. Johnston, P. J. Goulder, J. I. Mullins, E. S. Rosenberg, C. Brander, B. Korber, B. D. Walker, Enhanced detection of human immunodeficiency virus type 1-specific T-cell responses to highly variable regions by using peptides based on autologous virus sequences. J Virol, 77, 7330-7340 (2003).
79. A. T. Catanzaro, R. A. Koup, M. Roederer, R. T. Bailer, M. E. Enama, Z. Moodie, L. Gu, J. E. Martin, L. Novik, B. K. Chakrabarti, B. T. Butman, J. G. Gall, C. R. King, C. A. Andrews, R. Sheets, P. L. Gomez, J. R. Mascola, G. J. Nabel, B. S. Graham, Phase 1 safety and immunogenicity evaluation of a multiclade HIV-1 candidate vaccine delivered by a replication-defective recombinant adenovirus vector. J Infect Dis, 194, 1638-1649 (2006).
80. B. S. Graham, R. A. Koup, M. Roederer, R. T. Bailer, M. E. Enama, Z. Moodie, J. E. Martin, M. M. McCluskey, B. K. Chakrabarti, L. Lamoreaux, C. A. Andrews, P. L. Gomez, J. R. Mascola, G. J. Nabel, Phase 1 safety and immunogenicity evaluation of a multiclade HIV-1 DNA candidate vaccine. J Infect Dis, 194, 1650-1660 (2006).
81. M. S. Seaman, L. Xu, K. Beaudry, K. L. Martin, M. H. Beddall, A. Miura, A. Sambor, B. K. Chakrabarti, Y. Huang, R. Bailer, R. A. Koup, J. R. Mascola, G. J. Nabel, N. L. Letvin, Multiclade human immunodeficiency virus type 1 envelope immunogens elicit broad cellular and humoral immunity in rhesus monkeys. J Virol, 79, 2956-2963 (2005).
82. A. Bertoletti, A. Sette, F. V. Chisari, A. Penna, M. Levrero, M. De Carli, F. Fiaccadori, C. Ferrari, Natural variants of cytotoxic epitopes are T-cell receptor antagonists for antiviral cytotoxic T cells. Nature, 369, 407-410 (1994).
83. P. Klenerman, S. Rowland-Jones, S. McAdam, J. Edwards, S. Daenke, D. Lalloo, B. Koppe, W. Rosenberg, D. Boyd, A. Edwards, et al., Cytotoxic T-cell activity antagonized by naturally occurring HIV-1 Gag variants. Nature, 369, 403-407 (1994).
84. D. Basu, C. B. Williams, P. M. Allen, In vivo antagonism of a T cell response by an endogenously expressed ligand. Proc Natl Acad Sci U S A, 95, 14332-14336 (1998).
85. S. C. Gilbert, M. Plebanski, S. Gupta, J. Morris, M. Cox, M. Aidoo, D. Kwiatkowski, B. M. Greenwood, H. C. Whittle, A. V. Hill, Association of malaria parasite population structure, HLA, and immunological antagonism. Science, 279, 1173-1177 (1998).
86. L. L. Lau, J. Jiang, H. Shen, In vivo modulation of T cell responses and protective immunity by TCR antagonism during infection. J Immunol, 174, 7970-7976 (2005).
87. M. Plebanski, E. A. Lee, C. M. Hannan, K. L. Flanagan, S. C. Gilbert, M. B. Gravenor, A. V. Hill, Altered peptide ligands narrow the repertoire of cellular immune responses by interfering with T-cell priming. Nat Med, 5, 565-571 (1999).
88. W. Fischer, S. Perkins, J. Theiler, T. Bhattacharya, K. Yusim, R. Funkhouser, C. Kuiken, B. Haynes, N. L. Letvin, B. D. Walker, B. H. Hahn, B. T. Korber, Polyvalent vaccines for optimal coverage of potential T-cell epitopes in global HIV-1 variants. Nat Med, 13, 100-106 (2007).
89. M. Rolland, M. A. Jensen, D. C. Nickle, J. Yan, G. H. Learn, L. Heath, D. Weiner, J. I. Mullins, Reconstruction and function of Ancestral Center-Of-Tree (COT) HIV-I Proteins. J Virol, (2007).

90. T. M. Allen, J. Sidney, M.-F. del Guercio, R. L. Glickman, G. L. Lensmeyer, D. A. Wiebe, C. D. Pauza, R. P. Johnson, A. Sette, D. I. Watkins, Characterization of the peptide-binding motif of a rhesus MHC class I molecule (Mamu-A*01) that binds an immunodominant CTL epitope from SIV. J. Immunol., 160, 6062-6071 (1998).
91. T. Hanke, P. Szawlowski, R. E. Randall, Construction of solid matrix-antibody-antigen complexes containing simian immunodeficiency virus p27 using tag-specific monoclonal antibody and tag-linked antigen. J Gen Virol, 73, 653-660 (1992).
92. H. Takahashi, J. Cohen, A. Hosmalin, K. B. Cease, R. Houghten, J. L. Cornette, C. DeLisi, B. Moss, R. N. Germain, J. A. Berzofsky, An immunodominant epitope of the human immunodeficiency virus envelope glycoprotein gp160 recognized by class I major histocompatibility molecule-restricted murine cytotoxic T lymphocytes. Proc. Natl. Acad. Sci. USA, 85, 3105-3109 (1988).
93. www.iavi.org.
94. V. Appay, P. R. Dunbar, M. Callan, P. Klenerman, G. M. Gillespie, L. Papagno, G. S. Ogg, A. King, F. Lechner, C. A. Spina, S. Little, D. V. Havlir, D. D. Richman, N. Gruener, G. Pape, A. Waters, P. Easterbrook, M. Salio, V. Cerundolo, A. J. McMichael, S. L. Rowland-Jones, Memory CD8+ T cells vary in differentiation phenotype in different persistent virus infections. Nat Med, 8, 379-385 (2002).
95. M. J. Estcourt, S. Letourneau, A. J. McMichael, T. Hanke, Vaccine route, dose and type of delivery vector determine patterns of primary CD8+ T cell responses. Eur J Immunol, 35, 2532-2540 (2005).
96. M. Honda, R. Wang, W. P. Kong, M. Kanekiyo, W. Akahata, L. Xu, K. Matsuo, K. Natarajan, H. Robinson, T. E. Asher, D. A. Price, D. C. Douek, D. H. Margulies, G. J. Nabel, Different vaccine vectors delivering the same antigen elicit CD8+ T cell responses with distinct clonotype and epitope specificity. J Immunol, 183, 2425-2434 (2009).
97. B. Peng, L. R. Wang, V. R. Gomez-Roman, A. Davis-Warren, D. C. Montefiori, V. S. Kalyanaraman, D. Venzon, J. Zhao, E. Kan, T. J. Rowell, K. K. Murthy, I. Srivastava, S. W. Barnett, M. Robert-Guroff, Replicating rather than nonreplicating adenovirus-human immunodeficiency virus recombinant vaccines are better at eliciting potent cellular immunity and priming high-titer antibodies. J Virol, 79, 10200-10209 (2005).
98. C. C. Norbury, S. Basta, K. B. Donohue, D. C. Tscharke, M. F. Princiotta, P. Berglund, J. Gibbs, J. R. Bennink, J. W. Yewdell, CD8+ T cell cross-priming via transfer of proteasome substrates. Science, 304, 1318-1321 (2004).
99. L. Shen and K. L. Rock, Cellular protein is the source of cross-priming antigen in vivo. Proc Natl Acad Sci U S A, 101, 3035-3040 (2004).
100. M. C. Wolkers, N. Brouwenstijn, A. H. Bakker, M. Toebes, T. N. Schumacher, Antigen bias in T cell cross-priming. Science, 304, 1314-1317 (2004).
101. A. J. McMichael and T. Hanke, HIV vaccines 1983-2003. Nat Med, 9, 874-880 (2003).
102. S. P. Buchbinder, D. V. Mehrotra, A. Duerr, D. W. Fitzgerald, R. Mogg, D. Li, P. B. Gilbert, J. R. Lama, M. Marmor, C. Del Rio, M. J. McElrath, D. R. Casimiro, K. M. Gottesdiener, J. A. Chodakewitz, L. Corey, M. N. Robertson, Efficacy assessment of a cell-mediated immunity HIV-1 vaccine (the Step Study): a double-blind, randomised, placebo-controlled, test-of-concept trial. Lancet, 372, 1881-1893 (2008).
103. D. R. Casimiro, A. J. Bett, T. M. Fu, M. E. Davies, A. Tang, K. A. Wilson, M. Chen, R. Long, T. McKelvey, M. Chastain, S. Gurunathan, J. Tartaglia, E. A. Emini, J. Shiver, Heterologous human immunodeficiency virus type 1 priming-boosting immunization strategies involving replication-defective adenovirus and poxvirus vaccine vectors. J Virol, 78, 11434-11438 (2004).

104. A. Bridgeman, Y. Roshorm, L. J. Lockett, Z.-Z. Xu, R. Hopkins, J. Shaw, G. W. Both, T. Hanke, Ovine *Atadenovirus*, a novel and highly immunogenic vector in prime-boost studies of a candidate HIV-1 vaccine. Vaccine, In press (2009).
105. A. R. Thorner, A. A. Lemckert, J. Goudsmit, D. M. Lynch, B. A. Ewald, M. Denholtz, M. J. Havenga, D. H. Barouch, Immunogenicity of heterologous recombinant adenovirus prime-boost vaccine regimens is enhanced by circumventing vector cross-reactivity. J Virol, 80, 12009-12016 (2006).
106. R. R. Amara, F. Villinger, J. D. Altman, S. L. Lydy, S. P. O'Neil, S. I. Staprans, D. C. Montefiori, Y. Xu, J. G. Herndon, L. S. Wyatt, M. A. Candido, N. L. Kozyr, P. L. Earl, J. M. Smith, H. L. Ma, B. D. Grimm, M. L. Hulsey, J. Miller, H. M. McClure, J. M. McNicholl, B. Moss, H. L. Robinson, Control of a Mucosal Challenge and Prevention of AIDS by a Multiprotein DNA/MVA Vaccine. Science, 292, 69-74 (2001).
107. T. Hanke, N. Goonetilleke, A. J. McMichael, L. Dorrell, Clinical experience with plasmid DNA- and modified vaccinia vaccine Ankara (MVA)-vectored HIV-1 clade A vaccine inducing T cells. J Gen Virol, 88, 1-12 (2007).
108. T. Hanke, T. J. Blanchard, J. Schneider, C. M. Hannan, M. Becker, S. C. Gilbert, A. V. S. Hill, G. L. Smith, A. McMichael, Enhancement of MHC class I-restricted peptide-specific T cell induction by a DNA prime/MVA boost vaccination regime. Vaccine, 16, 439-445 (1998).
109. S. Santra, Y. Sun, B. Korioth-Schmitz, J. Fitzgerald, C. Charbonneau, G. Santos, M. S. Seaman, S. J. Ratcliffe, D. C. Montefiori, G. J. Nabel, H. C. Ertl, N. L. Letvin, Heterologous prime/boost immunizations of rhesus monkeys using chimpanzee adenovirus vectors. Vaccine, 27, 5837-5845 (2009).
110. J. M. Vuola, S. Keating, D. P. Webster, T. Berthoud, S. Dunachie, S. C. Gilbert, A. V. Hill, Differential immunogenicity of various heterologous prime-boost vaccine regimens using DNA and viral vectors in healthy volunteers. J Immunol, 174, 449-455 (2005).
111. M. J. Estcourt, A. J. McMichael, T. Hanke, DNA vaccines against human immunodeficiency virus type 1. Immunol Rev, 199, 144-155 (2004).
112. D. B. Weiner and R. C. Kennedy, Genetic vaccines. Sci. American, July, 34-41 (1999).
113. A. T. Catanzaro, M. Roederer, R. A. Koup, R. T. Bailer, M. E. Enama, M. C. Nason, J. E. Martin, S. Rucker, C. A. Andrews, P. L. Gomez, J. R. Mascola, G. J. Nabel, B. S. Graham, Phase I clinical evaluation of a six-plasmid multiclade HIV-1 DNA candidate vaccine. Vaccine, 25, 4085-4092 (2007).
114. S. J. McConkey, W. H. Reece, V. S. Moorthy, D. Webster, S. Dunachie, G. Butcher, J. M. Vuola, T. J. Blanchard, P. Gothard, K. Watkins, C. M. Hannan, S. Everaere, K. Brown, K. E. Kester, J. Cummings, J. Williams, D. G. Heppner, A. Pathan, K. Flanagan, N. Arulanantham, M. T. Roberts, M. Roy, G. L. Smith, J. Schneider, T. Peto, R. E. Sinden, S. C. Gilbert, A. V. Hill, Enhanced T-cell immunogenicity of plasmid DNA vaccines boosted by recombinant modified vaccinia virus Ankara in humans. Nat Med, 9, 729-735 (2003).
115. J. A. Tavel, J. E. Martin, G. G. Kelly, M. E. Enama, J. M. Shen, P. L. Gomez, C. A. Andrews, R. A. Koup, R. T. Bailer, J. A. Stein, M. Roederer, G. J. Nabel, B. S. Graham, Safety and immunogenicity of a Gag-Pol candidate HIV-1 DNA vaccine administered by a needle-free device in HIV-1-seronegative subjects. J Acquir Immune Defic Syndr, 44, 601-605 (2007).
116. M. J. Estcourt, A. J. Ramsay, A. Brooks, S. A. Thomson, C. J. Medveckzy, I. A. Ramshaw, Prime-boost immunization generates a high frequency, high-avidity CD8(+) cytotoxic T lymphocyte population. Int Immunol, 14, 31-37. (2002).

117. T. Hanke, J. Schneider, S. C. Gilbert, A. V. S. Hill, A. McMichael, DNA multi-CTL epitope vaccines for HIV and *Plasmodium falciparum*: Immunogenicity in mice. Vaccine, 16, 426-435 (1998).
118. T. Hanke and A. J. McMichael, Design and construction of an experimental HIV-1 vaccine for a year-2000 clinical trial in Kenya. Nat. Med., 6, 951-955 (2000).
119. S. G. Williams, R. M. Cranenburgh, A. M. E. Weiss, C. J. Wrighton, D. J. Sherratt, J. A. J. Hanak, Repressor titration: a novel system for selection and stable maintenance of recombinant plasmids. Nucleic Acids Res., 26, 2120-2124 (1998).
120. S. C. Gilbert, V. S. Moorthy, L. Andrews, A. A. Pathan, S. J. McConkey, J. M. Vuola, S. M. Keating, T. Berthoud, D. Webster, H. McShane, A. V. Hill, Synergistic DNA-MVA prime-boost vaccination regimes for malaria and tuberculosis. Vaccine, 24, 4554-4561 (2006).
121. M. Rosario, A. Bridgeman, E. D. Quakkelaar, M. F. Quigley, B. J. Hill, M. L. Knudsen, V. Ammendola, K. Ljungberg, N. Borthwick, E. J. Im, A. J. McMichael, J. W. Drijfhout, H. Y. Greenaway, V. Venturi, D. C. Douek, S. Colloca, P. Liljestrom, A. Nicosia, D. A. Price, C. J. Melief, T. Hanke, Long peptides induce polyfunctional T cells against conserved regions of HIV-1 with superior breadth to single-gene vaccines in macaques. Eur J Immunol, 40, 1973-1984 (2010).
122. A. Mayr, H. Stickl, H. K. Muller, K. Danner, H. Singer, Der Pockenimpfstamm MVA: Marker, genetische Struktur, Erfahrungen mit der parenteralen Schutzimpfung und Verhalten im abwehrgeschwachten Organismus. Zentralbl. Bakt. Hyg. I. (Abt. Orig. B), 167, 375-390 (1978).
123. T. Hanke, A. J. McMichael, R. S. Samuel, L. A. J. Powell, L. McLoughlin, S. J. Crome, A. Edlin, Lack of toxicity and persistence in the mouse associated with administration of candidate DNA- and modified vaccinia virus Ankara (MVA)-based HIV vaccines for Kenya. Vaccine, 21, 109-115 (2002).
124. T. Hanke, A. J. McMichael, M. J. Dennise, S. A. Sharpe, L. A. J. Powell, l. McLoughlin, S. J. Crome, Biodistribution and persistence of an MVA-vectored candidate HIV vaccine in SIV-infected rhesus macaques and SCID mice. Vaccine, 23, 1507-1514 (2005).
125. K. J. Stittelaar, T. Kuiken, R. L. de Swart, G. van Amerongen, H. W. Vos, H. G. Niesters, P. van Schalkwijk, T. van der Kwast, L. S. Wyatt, B. Moss, A. D. Osterhaus, Safety of modified vaccinia virus Ankara (MVA) in immune-suppressed macaques. Vaccine, 19, 3700-3709. (2001).
126. T. Hanke, T. J. Blanchard, J. Schneider, G. S. Ogg, R. Tan, M. Becker, S. C. Gilbert, A. V. S. Hill, G. L. Smith, A. McMichael, Immunogenicities of intravenous and intramuscular administrations of MVA-based multi-CTL epitope vaccine for HIV in mice. J. Gen. Virol., 79, 83-90 (1998).
127. E. J. Im, N. Saubi, G. Virgili, C. Sander, D. Teoh, J. M. Gatell, H. McShane, J. Joseph, T. Hanke, Vaccine platform for prevention of tuberculosis and mother-to-child transmission of human immunodeficiency virus type 1 through breastfeeding. J Virol, 81, 9408-9418 (2007).
128. T. Hanke, R. V. Samuel, T. J. Blanchard, V. C. Neumann, T. M. Allen, J. E. Boyson, A. S. Sharpe, N. Cook, G. L. Smith, D. I. Watkins, M. P. Cranage, A. McMichael, Effective induction of simian immunodeficiency virus-specific cytotoxic T lymphocytes in macaques by using a multiepitope gene and DNA prime-modified vaccinia virus Ankara boost vaccination regimen. J Virol, 73, 7524-7532 (1999).
129. E.-J. Im, K. di Gleria, A. J. McMichael, T. Hanke, Induction of long-lasting multi-specific $CD8^+$ T cells by a 4-component DNA-MVA/HIVA-RENTA candidate HIV-1 vaccine in rhesus macaques. Eur J Immunol, 36, 2574-2584 (2006).

130. H. L. Robinson, D. C. Montefiori, R. P. Johnson, K. H. Manson, M. L. Kalish, J. D. Lifson, T. A. Rizvi, S. Lu, S.-L. Hu, G. P. Mazzara, D. L. Panicali, J. G. Herndon, R. Glickman, M. A. Candido, S. L. Lydy, M. S. Wyand, H. M. McClure, Neutralizing antibody-independent containment of immunodeficiency virus challenges by DNA priming and recombinant pox virus booster immunizations. Nat. Med., 5, 526-534 (1999).
131. A. Seth, I. Ourmanov, J. E. Schmitz, M. J. Kuroda, M. A. Lifton, C. E. Nickerson, L. Wyatt, M. Carroll, B. Moss, D. Venzon, N. L. Letvin, V. M. Hirsch, Immunization with a modified vaccinia virus expressing simian immunodeficiency virus (SIV) Gag-Pol primes for an anamnestic Gag-specific cytotoxic T-lymphocyte response and is associated with reduction of viremia after SIV challenge. J. Virol., 74, 2502-2509 (2000).
132. A. Mayr, V. Hochstein-Mintzel, H. Stickl, Abstammung, Eigenschaften und Verwendung des attenuierten Vaccinia-Stammes MVA. Infection, 105, 6-14 (1975).
133. P. Bejon, N. Peshu, S. C. Gilbert, B. S. Lowe, C. S. Molyneux, J. Forsdyke, T. Lang, A. V. Hill, K. Marsh, Safety profile of the viral vectors of attenuated fowlpox strain FP9 and modified vaccinia virus Ankara recombinant for either of 2 preerythrocytic malaria antigens, ME-TRAP or the circumsporozoite protein, in children and adults in Kenya. Clin Infect Dis, 42, 1102-1110 (2006).
134. A. Cosma, R. Nagaraj, S. Buhler, J. Hinkula, D. H. Busch, G. Sutter, F. D. Goebel, V. Erfle, Therapeutic vaccination with MVA-HIV-1 nef elicits Nef-specific T-helper cell responses in chronically HIV-1 infected individuals. Vaccine, 22, 21-29 (2003).
135. E. Harrer, M. Bauerle, B. Ferstl, P. Chaplin, B. Petzold, L. Mateo, A. Handley, M. Tzatzaris, J. Vollmar, S. Bergmann, M. Rittmaier, K. Eismann, S. Muller, J. R. Kalden, B. Spriewald, D. Willbold, T. Harrer, Therapeutic vaccination of HIV-1-infected patients on HAART with a recombinant HIV-1 nef-expressing MVA: safety, immunogenicity and influence on viral load during treatment interruption. Antivir Ther, 10, 285-300 (2005).
136. E.-J. Im and T. Hanke, MVA as a vector for vaccines against HIV-1. Expert Rev Vaccines, 3, S89-97 (2004).
137. G. Sutter and C. Staib, Vaccinia vectors as candidate vaccines: the development of modified vaccinia virus Ankara for antigen delivery. Curr Drug Targets Infect Disord, 3, 263-271 (2003).
138. P. Bejon, J. Mwacharo, O. Kai, T. Mwangi, P. Milligan, S. Todryk, S. Keating, T. Lang, B. Lowe, C. Gikonyo, C. Molyneux, G. Fegan, S. C. Gilbert, N. Peshu, K. Marsh, A. V. Hill, A phase 2b randomised trial of the candidate malaria vaccines FP9 ME-TRAP and MVA ME-TRAP among children in Kenya. PLoS Clin Trials, 1, e29 (2006).
139. P. Bejon, J. Mwacharo, O. K. Kai, S. Todryk, S. Keating, T. Lang, S. C. Gilbert, N. Peshu, K. Marsh, A. V. Hill, Immunogenicity of the candidate malaria vaccines FP9 and modified vaccinia virus Ankara encoding the pre-erythrocytic antigen ME-TRAP in 1-6 year old children in a malaria endemic area. Vaccine, 24, 4709-4715 (2006).
140. T. C. Greenough, C. K. Cunningham, P. Muresan, M. McManus, D. Persaud, T. Fenton, P. Barker, A. Gaur, D. Panicali, J. L. Sullivan, K. Luzuriaga, Safety and immunogenicity of recombinant poxvirus HIV-1 vaccines in young adults on highly active antiretroviral therapy. Vaccine, 26, 6883-6893 (2008).
141. P. A. Bart, R. Goodall, T. Barber, A. Harari, A. Guimaraes-Walker, M. Khonkarly, N. C. Sheppard, Y. Bangala, M. J. Frachette, R. Wagner, P. Liljestrom, J. P. Kraehenbuhl, M. Girard, J. Goudsmit, M. Esteban, J. Heeney, Q. Sattentau, S. McCormack, A. Babiker, G. Pantaleo, J. Weber, EV01: a phase I trial in healthy HIV

negative volunteers to evaluate a clade C HIV vaccine, NYVAC-C undertaken by the EuroVacc Consortium. Vaccine, 26, 3153-3161 (2008).

142. G. de Bruyn, A. J. Rossini, Y. L. Chiu, D. Holman, M. L. Elizaga, S. E. Frey, D. Burke, T. G. Evans, L. Corey, M. C. Keefer, Safety profile of recombinant canarypox HIV vaccines. Vaccine, 22, 704-713 (2004).

143. N. Kanesa-thasan, J. J. Smucny, C. H. Hoke, D. H. Marks, E. Konishi, I. Kurane, D. B. Tang, D. W. Vaughn, P. W. Mason, R. E. Shope, Safety and immunogenicity of NYVAC-JEV and ALVAC-JEV attenuated recombinant Japanese encephalitis virus--poxvirus vaccines in vaccinia-nonimmune and vaccinia-immune humans. Vaccine, 19, 483-491 (2000).

144. S. McCormack, W. Stohr, T. Barber, P. A. Bart, A. Harari, C. Moog, D. Ciuffreda, C. Cellerai, M. Cowen, R. Gamboni, S. Burnet, K. Legg, E. Brodnicki, H. Wolf, R. Wagner, J. Heeney, M. J. Frachette, J. Tartaglia, A. Babiker, G. Pantaleo, J. Weber, EV02: a Phase I trial to compare the safety and immunogenicity of HIV DNA-C prime-NYVAC-C boost to NYVAC-C alone. Vaccine, 26, 3162-3174 (2008).

145. C. F. Ockenhouse, P. F. Sun, D. E. Lanar, B. T. Wellde, B. T. Hall, K. Kester, J. A. Stoute, A. Magill, U. Krzych, L. Farley, R. A. Wirtz, J. C. Sadoff, D. C. Kaslow, S. Kumar, L. W. Church, J. M. Crutcher, B. Wizel, S. Hoffman, A. Lalvani, A. V. Hill, J. A. Tine, K. P. Guito, C. de Taisne, R. Anders, W. R. Ballou, et al., Phase I/IIa safety, immunogenicity, and efficacy trial of NYVAC-Pf7, a pox-vectored, multiantigen, multistage vaccine candidate for Plasmodium falciparum malaria. J Infect Dis, 177, 1664-1673 (1998).

146. E. J. McFarland, D. C. Johnson, P. Muresan, T. Fenton, G. D. Tomaras, J. McNamara, J. S. Read, S. D. Douglas, J. Deville, M. Gurwith, S. Gurunathan, J. S. Lambert, HIV-1 vaccine induced immune responses in newborns of HIV-1 infected mothers. Aids, 20, 1481-1489 (2006).

147. H. McShane, A. A. Pathan, C. R. Sander, S. M. Keating, S. C. Gilbert, K. Huygen, H. A. Fletcher, A. V. Hill, Recombinant modified vaccinia virus Ankara expressing antigen 85A boosts BCG-primed and naturally acquired antimycobacterial immunity in humans. Nat Med, 10, 1240-1244 (2004).

148. L. Dorrell, H. Yang, A. K. Iversen, C. Conlon, A. Suttill, M. Lancaster, T. Dong, I. Cebere, A. Edwards, S. Rowland-Jones, T. Hanke, A. J. McMichael, Therapeutic immunization of highly active antiretroviral therapy-treated HIV-1-infected patients: safety and immunogenicity of an HIV-1 gag/poly-epitope DNA vaccine. Aids, 19, 1321-1323 (2005).

149. J. W. Shiver, T. M. Fu, L. Chen, D. R. Casimiro, M. E. Davies, R. K. Evans, Z. Q. Zhang, A. J. Simon, W. L. Trigona, S. A. Dubey, L. Huang, V. A. Harris, R. S. Long, X. Liang, L. Handt, W. A. Schleif, L. Zhu, D. C. Freed, N. V. Persaud, L. Guan, K. S. Punt, A. Tang, M. Chen, K. A. Wilson, K. B. Collins, G. J. Heidecker, V. R. Fernandez, H. C. Perry, J. G. Joyce, K. M. Grimm, J. C. Cook, P. M. Keller, D. S. Kresock, H. Mach, R. D. Troutman, L. A. Isopi, D. M. Williams, Z. Xu, K. E. Bohannon, D. B. Volkin, D. C. Montefiori, A. Miura, G. R. Krivulka, M. A. Lifton, M. J. Kuroda, J. E. Schmitz, N. L. Letvin, M. J. Caulfield, A. J. Bett, R. Youil, D. C. Kaslow, E. A. Emini, Replication-incompetent adenoviral vaccine vector elicits effective anti-immunodeficiency-virus immunity. Nature, 415, 331-335. (2002).

150. N. Tatsis, L. Tesema, E. R. Robinson, W. Giles-Davis, K. McCoy, G. P. Gao, J. M. Wilson, H. C. Ertl, Chimpanzee-origin adenovirus vectors as vaccine carriers. Gene Ther, 13, 421-429 (2006).

151. E. Nwanegbo, E. Vardas, W. Gao, H. Whittle, H. Sun, D. Rowe, P. D. Robbins, A. Gambotto, Prevalence of neutralizing antibodies to adenoviral serotypes 5 and 35 in

the adult populations of The Gambia, South Africa, and the United States. Clin Diagn Lab Immunol, 11, 351-357 (2004).

152. D. Peruzzi, S. Dharmapuri, A. Cirillo, B. E. Bruni, A. Nicosia, R. Cortese, S. Colloca, G. Ciliberto, N. La Monica, L. Aurisicchio, A novel Chimpanzee serotype-based adenoviral vector as delivery tool for cancer vaccines. Vaccine, 27, 1293-1300 (2009).

153. M. Rosario, A. Bridgeman, E. D. Quakkelaar, M. F. Quigley, B. J. Hill, M. L. Knudsen, V. Ammendola, K. Ljungberg, N. Borthwick, E.-J. Im, A. J. McMichael, J. W. Drijfhout, H. Y. Greenaway, V. Venturi, D. C. Douek, S. Colloca, D. I. Watkins, P. Liljeström, A. Nicosia, D. A. Price, C. J. M. Melief, T. Hanke, Long peptides induce polyfunctional T cells against conserved regions of HIV with superior breadth to single-gene vaccines in macaques. (Submitted).

APPENDIX A - SCHEDULE OF PROCEDURES

Stage 1 - Low dose of ChAdV63.HIVconsv

**Procedures / Study week**	**Scrn**	**0**	**1**	**2**	**4**	**8**	**16**	**28**
Visit number	1	2	3	4	5	6	7	8
Visit windows (days)	(≤ -28)	0	±3	±3	±3	±7	±7	±7
Informed consent	X							
Confirm eligibility	X	X						
ChAdV63.HIVconsv $5x10^9$ vp i.m.		X						
HIV-1 Risk Assessment	X							
Medical History	X							
Interim Medical History		X	X	X	X	X	X	X
General Physical Exam	X	X	X	X	X	X	X	X
Adverse Events			X	X	X	X	X	X
Concomitant Medications	X	X	X	X	X	X	X	X
Local and Systemic Reactogenicity Assessment		X	X	X	X	X	X	X
HLA Typing	X							
HBsAg, HCV Antibodies, Syphilis	X							
Serum HIV-1 ELISA and HIV-1 test counselling	X							X
Urinalysis	X							
Routine Haematology, Biochemistry	X	X	X	X	X	X		X
Pregnancy Test (if applicable)	X	X						X
Cellular Immunogenicity Assays	X	X	X	X	X	X	X	X

Stage 2- CM or PP

**Procedure / Study week**	**Scrn**	**0**	**1**	**2**	**4**	**8**	**9**	**12**	**20**	**28**
Visit number	1	2	3	4	5	6	7	8	9	10
Visit windows (days)	(≤ -28)	0	±3	±3	±3	±3	±3	±3	±7	±7
Informed consent	X									
Confirm eligibility	X	X								
Randomisation		X								
ChAdV63.HIVconv $5x10^{10}$ vp or placebo i.m.		X								
MVA.HIVconsv $2x10^{8}$ pfu or placebo i.m.						X				
HIV-1 Risk Assessment	X									
Medical History	X									
Interim Medical History		X	X	X	X	X	X	X	X	X
General Physical Exam	X	X	X	X	X	X	X	X	X	X
Adverse Events			X	X	X	X	X	X	X	X
Concomitant Medications	X	X	X	X	X	X	X	X	X	X
Local and Systemic Reactogenicity Assessment		X	X	X	X	X	X	X	X	X
HLA Typing	X									
HBsAg, HCV Antibodies, Syphilis	X									
Serum HIV-1 ELISA and HIV-1 test counselling	X									X
Urinalysis	X									
Routine Haematology, Biochemistry	X	X	X			X	X			X
Pregnancy Test (if applicable)	X	X				X				X
Cellular Immunogenicity Assays	X	X	X	X	X	X	X	X	X	X

Stage 3 - DDDCM or PPPPP

**Procedure / Study week**	**Scrn**	**0**	**4**	**8**	**12**	**13**	**14**	**20**	**21**	**22**	**28**
Visit number	1	2	3	4	5	6	7	8	9	10	11
Visit windows (days)	(≤ -28)	0	±3	±3	±3	±3	±3	±3	±3	±3	±7
Informed consent	X										
Confirm eligibility	X	X									
Randomisation		X									
pSG2.HIVconsv DNA 4 mg or placebo i.m.		X	X	X							
ChAdV63.HIVconv $5x10^{10}$ vp or placebo i.m.					X						
MVA.HIVconsv $2x10^{8}$ pfu or placebo i.m.								X			
HIV-1 Risk Assessment	X										
Medical History	X										
Interim Medical History		X	X	X	X	X	X	X	X	X	X
General Physical Exam	X	X	X	X	X	X	X	X	X	X	X
Adverse Events			X	X	X	X	X	X	X	X	X
Concomitant Medications	X	X	X	X	X	X	X	X	X	X	X
Local and Systemic Reactogenicity Assessment		X	X	X	X	X	X	X	X	X	X
HLA Typing	X										
HBsAg, HCV Antibodies, Syphilis	X										
Serum HIV-1 ELISA and HIV-1 test counselling	X										X
Urinalysis	X										
Routine Haematology, Biochemistry	X	X			X	X		X	X		X
Pregnancy Test (if applicable)	X	X	X	X	X			X			X
Cellular Immunogenicity Assays	X	X		X	X	X	X	X	X	X	X

Stage 4 - DDDMC or PPPPP

**Study week**	**Scrn**	**0**	**4**	**8**	**12**	**13**	**16**	**17**	**18**	**24**	**28**
Visit number	1	2	3	4	5	6	7	8	9	10	11
Visit windows (days)	(≤ -28)	0	±3	±3	±3	±3	±3	±3	±3	±3	±7
Informed consent	X										
Confirm eligibility	X	X									
Randomisation		X									
pSG2.HIVconsv DNA 4 mg or placebo i.m.		X	X	X							
MVA.HIVconsv $2x10^8$ pfu or placebo i.m.					X						
ChAdV63.HIVconv $5x10^{10}$ vp or placebo i.m.							X				
HIV-1 Risk Assessment	X										
Medical History	X										
Interim Medical History		X	X	X	X	X	X	X	X	X	X
General Physical Exam	X	X	X	X	X	X	X	X	X	X	X
Adverse Events			X	X	X	X	X	X	X	X	X
Concomitant Medications	X	X	X	X	X	X	X	X	X	X	X
Local and Systemic Reactogenicity Assessment		X	X	X	X	X	X	X	X	X	X
HLA Typing	X										
HBsAg, HCV Antibodies, Syphilis	X										
Serum HIV-1 ELISA and HIV-1 test counselling	X										X
Urinalysis	X										
Routine Haematology, Biochemistry	X	X			X	X	X	X			X
Pregnancy Test (if applicable)	X	X	X	X	X		X				X
Cellular Immunogenicity Assays	X	X		X	X	X	X	X	X	X	X

APPENDIX B - VOLUNTEER INVITATION LETTER VERSION 2.0

HIV-CORE 002 STUDY

West London REC 1 No. 10/H0707/52 **Eudract No. 2010-018439-16**

Are you interested in taking part in a clinical study aimed at testing a series of new vaccines designed to help the immune system control HIV?

You should be aged 18-50 years, HIV-negative, healthy and willing to discuss HIV infection risks. If you engage in behaviour which could put you at high risk of HIV infection you should not take part in this study. Women who are interested in participating should not be pregnant or intending to become pregnant during the study period.

If you chose to participate, you will receive between 1 and 5 vaccinations over a 7-month period. You will also be required to give a blood sample at each study visit during this time.

The Clinical trial is taking place at the University of Oxford, Centre for Clinical Vaccinology and Tropical Medicine, Churchill Hospital, Headington, Oxford OX3 7LJ.

You will be compensated for your time and for the additional travelling costs to and from the hospital.

Participating in this trial does not mean you will become protected against HIV. If you take part, you will be advised on how to protect yourself against HIV infection.

If you are interested, please contact the individuals below for further information.

Thank you for your consideration.

Dr. Antony Black (Project Manager) tel: 01865 857453
email:antony.black@ndm.ox.ac.uk

Dr. Lucy Dorrell (Principal Investigator) tel: 01865 222145
email:lucy.dorrell@imm.ox.ac.uk

Dr Tomas Hanke (Chief Investigator) tel: 01865 617630
email:tomas.hanke@ndm.ox.ac.uk

APPENDIX C - PARTICIPANT INFORMATION LEAFLET VERSION 2.0

HIV-CORE 002 STUDY

Sponsor: University of Oxford

West London REC 1 No. 10/H0707/52 **Eudract No. 2010-018439-16**

PARTICIPANT INFORMATION LEAFLET

Thank you for showing interest in this study. Before you decide to take part, it is important for you to understand why this research is being done and what it will involve. You should be aged 18-50 years, HIV-negative and healthy. Women who are interested in participating should not be pregnant or intending to become pregnant during the study period. Participating in this trial does not necessarily mean that you will become immune to HIV. If you take part, you will be advised on how to protect yourself against HIV infection. Please take time to read the following information carefully and discuss it with friends, relatives or your GP if you wish.

Please ask us if there is anything that is not clear or if you would like more information.

Why is this study being done?
Approximately 33 million people are living with HIV-1, the virus that causes AIDS, and it is estimated that over 7000 new infections occurred each day in 2008. Vaccination is the most effective way to prevent infection, but it has proved extremely difficult to develop an effective vaccine against HIV. One of the main reasons for this is the enormous variation in HIV-1 strains around the world, which is caused by the extraordinary ability of the virus to change its genetic material.

What is the vaccine being tested in this study?
To combat the variability of HIV, a new type of vaccine has been developed at the Medical Research Council laboratory in Oxford. This vaccine, called HIVconsv, consists of a string of HIV-1 gene fragments which have been specially chosen because they code for the least variable (also called highly conserved) protein regions of the virus. The aim is to drive the immune system towards making immune responses to these highly conserved regions, which are thought to be the weak spots within the virus and which the virus cannot easily change and escape.

In order to be effective in people, the HIVconsv vaccine has to be delivered by a carrier (also called a vector), which is either a harmless virus or DNA from a harmless bacterium. Research in Oxford has shown that the strongest immune responses against HIV-1 are achieved when the HIVconsv vaccine is given as a sequence of vaccinations using three different vectors. These vectors are: (1) DNA, (2) a weakened non-replicating chimpanzee

virus (an adenovirus called ChAdV-63), which is similar to human common cold viruses, and (3) a type of smallpox vaccine called modified vaccinia virus Ankara (MVA), which also cannot replicate.

The three vaccines are completely synthetic and do not contain live HIV-1, therefore they cannot give people HIV-1 infection or AIDS. However, as only the MVA vaccine has been tested before in man, the aim of this trial is to confirm that they are safe in people.

What would I be vaccinated with?
The trial has been designed in four stages. The participants will be sequentially allocated into each stage.

The purpose of Stage 1 is to test the chimpanzee adenovirus vaccine, ChAdV63.HIVconsv on its own, to ensure that it is safe, before testing it in combination with the other two vaccines. Two volunteers will receive a low dose of this vaccine and will be monitored for any side effects.

Stages 2, 3 and 4 have been designed as a randomised placebo-controlled trial. A randomised trial is carried out because we want to know whether any side effects a study participant might experience are due to the vaccine or simply a chance finding, so we need to make comparisons. Also, if we see that a study participant makes an immune response to HIV-1, we want to be certain that these are due to the vaccine. The placebo injection will contain only liquid solution without the vaccine. People will be put into different groups and then compared. The groups are selected by a computer, which has no information about the individual – i.e. like tossing a coin. A total of 30 individuals will be recruited into stages 2-4 of the trial.

- In stage 2, 10 individuals will receive EITHER one injection of a standard dose of ChAdV63.HIVconsv followed by an injection of MVA.HIVconsv 8 weeks later OR two placebo injections 8 weeks apart.

- In stage 3, 10 individuals will receive EITHER three injections of the DNA vaccine (‘pSG2.HIVconsv’) followed by one injection of ChAdV63.HIVconsv at four-weekly intervals and then MVA.HIVconsv 8 weeks later OR five injections of placebo over 20 weeks.

- In stage 4, 10 individuals will receive EITHER three injections of pSG2.HIVconsv DNA followed by one injection of MVA.HIVconsv and then one injection of ChAdV63.HIVconsv at four-weekly intervals OR five injections of placebo at four-weekly intervals.

Thus, individuals in stages 2, 3 and 4 will have a four in five (80%) chance of receiving the HIV-1 vaccine combination and a one in five (20%) chance of getting the placebo. As this is a single (placebo not matched)-blind trial, your study doctor or study nurse will know whether you are receiving the actual vaccine or a dummy although you will not.

Do I have to take part?
It is entirely your decision as to whether or not to take part. If you do decide to take part you will be asked to sign a consent form. After you have done so, you are still free to withdraw at any time and without giving a reason.

How often do I need to attend?
Depending on the stage of the trial that you enter, you will be asked to attend for a maximum of 11 visits over a period of 7-8 months.

What do I have to do if I agree to take part?
On the first visit (screening visit) you will spend some time talking to the study doctor or nurse, to make sure that you have been fully informed and are happy to give written consent to take part in the study. If you decide to take part you will be given a copy of the consent form you sign to keep, together with this information sheet. You will be given an appointment schedule for the study visits. If appropriate, you will be asked to ensure that you use effective contraception throughout the study.

You will be asked questions about your health and any medications and will have a physical examination. A blood sample will be taken in order to confirm that you are eligible to take part. If you do not qualify for this study for any reason, this will be discussed with you. If a minor abnormality is noted on a screening blood test, a repeat test might be required to verify it. We will see you within two weeks to give you the results and discuss whether you might still be eligible for vaccination.

On every subsequent visit you will be asked questions about your health and at some visits, we will carry out a physical examination.

What will happen to the blood sample that I give?
You will have between 40-80 ml (roughly 3-5 tablespoons) of blood taken at each visit (on average, just over 3 tablespoons) with a total of no more than 600 ml over a period of six months. 600 ml is just over the amount taken at a Blood Donation Centre on a single visit. The blood tests will include research laboratory tests designed to measure your immune response to HIV-1, evidence of other infections, your tissue type (similar to blood group), and occasionally, a full blood count, blood chemistry and liver function tests. Some of each sample will be stored at each visit for future tests. If you choose to withdraw from the study, any of your anonymised samples collected prior to withdrawal may be used for future tests.
The chart below gives a summary of will happen at each visit, for each stage of the trial

Stage 1

**Number of visits**	8
**Duration of study**	32 weeks
**Vaccination visits**	Week 0
**Blood sample**	Screening visit, weeks 0, 1, 2, 4, 8, 16 and 28

Stage 2

**Number of visits**	10
**Duration of study**	32 weeks
**Vaccination visits**	Weeks 0 and 8
**Blood sample**	Screening visit, weeks 0, 1, 2, 4, 8, 9, 12, 20 and 28

Stage 3

**Number of visits**	11
**Duration of study**	32 weeks
**Vaccination visits**	Weeks 0, 4, 8, 12 and 20

**Blood sample**	Screening visit, weeks 0, 8, 12, 13, 14, 20, 21, 22 and 28

Stage 4

**Number of visits**	11
**Duration of study**	32 weeks
**Vaccination visits**	Weeks 0, 4, 8, 12 and 16
**Blood sample**	Screening visit, weeks 0, 8, 12, 13, 16, 17, 18, 24 and 28

On the vaccination visits you will be given an injection into the muscle over the shoulder region of both arms. You will be asked to spend half an hour at the clinic to ensure that there is no serious reaction to the vaccine. Before going home, you will be given a diary card and a number to call the doctor or nurse in case you have any side effects or concerns about the vaccine. During the next three days you will be asked to keep a daily record of your temperature. You will be telephoned by a doctor or nurse on the third day after the vaccination to check whether you have had any side effects.

What are the risks of taking part?

Risks of DNA (pSG2.HIVconsv) vaccination
This vaccine has not been tested previously in man. DNA vectors, which are similar to the vector used in pSG2.HIVconsv, have been tested extensively in humans (HIV-1 and malaria trials) and shown to be safe. These include a first-generation HIV vaccine, DNA.HIVA, which was also developed in Oxford and tested in combination with MVA.HIVA, without harmful effects.

Risks of ChAdV63.HIVconsv vaccination.
This vaccine has not been tested previously in man. The chimpanzee vector, ChAdV-63, has also been used as a carrier for a vaccine against malaria which has been given to healthy people in Oxford, without any harmful effects. Like MVA, this virus is weakened so that it cannot replicate in humans.

Risks of MVA.HIVconsv vaccination.
The MVA vector was used as a smallpox vaccine about 40 years ago in over 120 000 people, without any problems. Currently, MVA is being developed as a vaccine vector for a number of diseases such as malaria, tuberculosis, hepatitis and cancer.

The MVA.HIVconsv vaccine is currently being tested in Oxford in people who already are HIV-1 positive and who are receiving effective antiretroviral treatment. The results of this trial will be available once it is completed (2011). In addition, before this trial a first-generation HIV-1 vaccine, which was developed in Oxford, called MVA.HIVA, was tested in over 370 healthy and HIV-1-positive people in the UK, Europe and Africa. MVA.HIVA was safe and did not cause any significant or serious side effects. Furthermore, it was able to boost immune responses to HIV-1 to some degree.

For the reasons given above, we do not expect to see any serious adverse reactions when a series of two or three vaccines is given. No serious side effects were observed in experimental animals receiving these vaccines. However, it is possible that any new combination of vaccines may produce unexpected and new side effects in humans.

Any vaccination has the potential to cause the following:

- redness, pain, swelling, itching, bruising, a warm feeling;
- flu-like symptoms such as fever, chills, muscle aches and pains, headaches, nausea, dizziness and fatigue;
- allergic reactions such as itchy rash, low blood pressure, sudden body swelling, serious breathing difficulty.
- a temporary ache around the injection site.

For these reasons, we will observe you in clinic for 30 minutes after vaccination.

Risks of taking blood samples (venepuncture).
Having blood taken may cause some discomfort, bleeding, or bruising where the needle enters the body and, in rare cases, light-headedness and fainting.
We do not foresee that the amount of blood taken during the study will cause harm to your health. In previous studies we conducted, in which volunteers gave 8 blood samples of 50 ml (about 3 tablespoons) each over a 6-month period, no-one developed anaemia during this time.

False positive results on HIV-1 tests
There is a very low chance that the vaccines may cause some HIV-1 tests to give false HIV positive results following vaccination. If this does occur, we will test again using different methods that will be able to distinguish true infection from a result due to vaccination. Should you be required to share this information with a third party, we will be happy to provide verbal or written clarification as needed.

Social harms
It is possible that by taking part in this study, others may perceive you to be at risk of HIV infection or stigmatise you. If you have any concerns or difficulties, trial staff will provide assistance and support.

Will the study benefit me?
You may receive no direct benefit from this study**.** The aim of this study is to see whether a two or three vaccine combination stimulates an immune response against HIV-1, but you will not be protected against HIV-1 infection or AIDS. However, knowledge gained may in the future help others to avoid HIV-1 infection.

Can I take part in this study if I am pregnant?
If you are trying or planning to become pregnant or breast-feeding you should not take part in this study. The safety of the vaccines in pregnancy is not known. You or your partner will be asked to use an effective method of contraception during the study, if this is appropriate and if you are not already doing so. Contraception will need to be continued for the duration of the study. If you become pregnant during the study, you should tell us immediately.

Will I be able to participate in other trials in the future if I have received this vaccine?
This depends on the specific criteria of each trial and it would need to be discussed on an individual basis. If you decide not to take part, or to withdraw from this study because you wish to ensure that you are eligible for another study, we will respect your decision.

Will the information from the study be confidential?
Yes, all your records will be kept confidential. In addition to the doctors and nurses that you meet, other trial staff involved who have access to your records are equally bound to respect your confidentiality. The data will be collected and kept in accordance with the Data

Protection Act 1998. All data will be coded, which means that your name will not be used on any research report or samples collected during the study. The study may be audited by responsible members of the University of Oxford to ensure compliance with the Research Governance Framework.

What if new information becomes available?
You will be told about any new information learned during the course of the study that might cause you change your mind about staying in the study.

Who reviews the study?
The trial procedures have been reviewed and approved by the Gene Therapy Advisory Committee/Research Ethics Committee. They and an independent committee of experts (Data Monitoring Committee) will monitor the progress of the trial, paying close attention to events that could have an impact on the health of the participants.

What happens to the results at the end of the study?
You will be informed of the results of this trial by one of the study doctors. The data gathered from the research will be submitted for publication. You will not be identified in any report or publication.

Who sponsors the study?
This study is sponsored by the University of Oxford.

Research-Related Injury
We do not expect you to suffer any injury as a result of participating in this study. Medical care will be organised, free of charge, in the unlikely event that an injury related to the study does occur.

The University has arrangements in place to provide for harm arising from participation in the study for which the University is the Research Sponsor.

Will I be paid for taking part in this study?
You will receive a payment of £25 for each study visit you make, in respect of the time you have given up for this study and to cover any travel costs.

What happens if I have a complaint?
If you wish to complain about any aspect of the way in which you have been approached or treated during the course of this study, you should contact the Chief Investigator, Tomas Hanke, on 01865-222355 or you may contact the University of Oxford Clinical Trials and Research Governance (CTRG) office on 01865 857939 or the head of CTRG, email heather.house@admin.ox.ac.uk

Who can I contact for more information?
For questions about this study please contact:

Trial Physician
University of Oxford, Centre for Clinical Vaccinology and Tropical Medicine, Churchill Hospital, Headington, Oxford OX3 7LJ Tel: 01865 insert number Fax: 01865 insert number

If you have questions you would prefer to discuss with a staff member who is not directly involved in this research, please contact:

Dr Brian Angus, Consultant Physician
Nuffield Department of Clinical Medicine
University of Oxford
John Radcliffe Hospital, Oxford OX3 9DU
Tel: 01865 221325; email: brian.angus@ndm.ox.ac.uk

APPENDIX D – CONSENT FORM VERSION 1.0

UNIVERSITY OF OXFORD

HIV-CORE 002 STUDY CONSENT FORM
West London REC 1 No. 10/H0707/52
Chief Investigator: Dr Tomas Hanke

Participant identification number for this study: ________

Please initial the boxes

1. I confirm that I have read and understood the information sheet dated………… version (…………..) for the above study. I have had the opportunity to consider the information, ask questions and have had these answered satisfactorily. ☐

2. I understand that my participation is voluntary and I am free to withdraw at any time without giving reason, and without my medical care or legal rights being affected. If I choose to withdraw, I agree to the use of any anonymised sample or data that have been collected prior to withdrawal. ☐

3. I understand that data collected during the study and relevant sections of my research study notes may be looked at by responsible members of the University of Oxford involved in the running of the study, or other regulatory authorities, for the purposes of auditing and monitoring. I give permission for these individuals to have access to my records. ☐

4. I agree to take part in the HIV-CORE 002 study. ☐

5. I understand all blood samples will be considered a gift to the University of Oxford and I will not gain any direct personal benefit from this. ☐

6. **(Optional)** I agree to the use of any sample remaining at the end of the study in future unspecified research. ☐

7. I agree to my GP being informed of my participation in this study. ☐

______________________ ____________ ______________________

Name of volunteer Signature Date

______________________ ____________ ______________________

Name of person taking consent Signature Date

When completed, 1 for volunteer; 1 for researcher (site file);

APPENDIX E - PARTICIPANT DIARY CARD

HIV-CORE 002 STUDY

West London REC 1 No. 10/H0707/52

DIARY CARD

Participant ID number for this study ___________

Instructions:

- Please record the date, time and temperature on the card below.
- Use the thermometer provided by the study team. It will provide the temperature in Celsius degrees.
- Record the temperature on the evening of your vaccination (D0) and approximately at the same time on the following 3 days (72 hours).
- If you forget to record your temperature in the evening, just do it at as soon as you remember specifying the date and time of the measurement below.
- If you feel feverish over the first 72 hour following vaccination and take any additional measurements, please record them on the lines below the table.
- If you have any questions, or need to contact a member of the trial team in an emergency at any time, contact INSERT NAME on: *[insert telephone number*].

	**Date**	**Time**	**Temperature**
**Day 0 evening**			
**Day 1 evening**			
**Day 2 evening**			
**Day 3 evening**			

APPENDIX F - SAMPLE GP LETTER VERSION 1.0

MRC Human Immunology Unit
The Jenner Institute
ORCRB
Roosevelt Drive
Oxford OX3 7DQ
Tel: 01865 617630
Fax: 01865 617608
tomas.hanke@ndm.ox.ac.uk

Date

Dear Dr

Re: [*participant's name, address, DOB*)

HIV-CORE 002 study **West London REC 1 No. 10/H0707/52**

I am writing to inform you, with [*participant's name*] permission, that s/he is participating in a clinical study evaluating the safety and immunogenicity of 3 candidate HIV-1 vaccines, MVA.HIVconsv, pSG2.HIVconsv and ChAdV63.HIVconsv in healthy HIV-1 negative adults. These are non-replicating vaccines comprising an attenuated vaccinia virus, a DNA vaccine and an attenuated adenovirus, which have been engineered to carry selected synthetic HIV-1 gene sequences.

There is a very small possibility that participants receiving the trial vaccines will develop potentially life-long false positive HIV test results. Any positive HIV test will be investigated further by tests that can distinguish between vaccine-induced responses and genuine HIV infection. Written clarification will be provided if requested.

If you have any queries please do not hesitate to contact me at the above address.

Yours sincerely,

Dr Tomas Hanke
Chief Investigator

APPENDIX G - ADVERSE EVENT SEVERITY ASSESSMENT TABLE

Adapted from Division of AIDS, 2004

Abbreviations: ADL- activities of daily living; LLN – lower limit of normal; ULN – upper limit of normal

**CLINICAL**				
**PARAMETER**	**GRADE 1**	**GRADE 2**	**GRADE 3**	**GRADE 4**
**SYSTEMIC EVENTS**				
Acute systemic allergic reaction	Localised urticaria; no medical intervention indicated	Localised urticaria with medical intervention indicated OR mild angioedema with medical intervention indicated	Generalised urticaria OR angioedema with medical intervention indicated OR symptomatic mild bronchospasm	Acute anaphylaxis OR life-threatening bronchospasm OR laryngeal oedema
Chills / rigors	Symptoms causing minimal or no interference with ADL	Symptoms causing greater than minimal interference with ADL	Symptoms causing inability to perform ADL	NA
Fatigue / Malaise	Symptoms causing minimal or no interference with ADL	Symptoms causing greater than minimal interference with ADL	Symptoms causing inability to perform ADL	Incapacitating symptoms causing inability to perform basic self-care
Pain (other than pain at injection site) – indicate body site	Pain causing minimal or no interference with ADL	Pain causing greater than minimal interference with ADL	Pain causing inability to perform AD	Disabling pain causing inability to perform basic self-care OR requiring hospitalisation (other than to Accident and Emergency Dept)
Headache	Symptoms causing minimal or no interference with ADL	Symptoms causing greater than minimal interference with ADL	Symptoms causing inability to perform ADL	Symptoms causing inability to perform basic self-care OR requiring hospitalisation (other than to Accident and Emergency Dept) OR headache with significant impairment of alertness or other neurological function
Fever (non-axillary)	37.7 – 38.6°C	38.7 – 39.3°C	39.3 – 40.5°C	> 40.5°C
**INJECTION SITE REACTIONS**				

Injection site pain (pain without touching) OR Tenderness (pain when area is touched)	Pain / tenderness causing no or minimal limitation of use of limb	Pain / tenderness limiting use of limb OR causing greater than minimal interference with ADL	Pain / tenderness causing inability to perform ADL	Pain / tenderness causing inability to perform basic self-care OR requiring hospitalisation (other than to Accident and Emergency Dept) indicated
Pruritus associated with injection	Localised to injection site and relieved spontaneously or with < 48 hours' treatment	Itching beyond the injection site but not generalised OR localised requiring > 48 hours' treatment	Generalised and causing inability to perform ADL	NA
Localised injection site reaction	Erythema or induration 5x5 cm-9x9 cm	Erythema or induration or oedema > 9 cm, any diameter	Ulceration OR secondary infection OR phlebitis OR sterile abscess OR drainage	Necrosis (involving dermis or deeper tissues)
**SKIN**				
Cutaneous reaction - rash	Localised macular rash	Diffuse macular or maculo-papular rash OR Target lesions	Diffuse macular or maculo-papular rash with vesicles or limited number of bullae OR superficial ulcerations of mucous membrane limited to one site	Extensive or generalised bullous lesions OR Stevens-Johnson syndrome OR ulceration of mucous membrane involving two or more distinct mucosal sites or toxic epidermal necrolysis
Pruritis (itching, no skin lesions)	Itching causing no or minimal interference with ADL	Itching causing greater than minimal interference with ADL	Itching causing inability to perform ADL	NA
Alopecia	Thinning detectable by study participant	Thinning or patchy loss detectable by healthcare provider	Complete hair loss	NA
**CARDIOVASCULAR**				
Cardiac Arrhythmia	Asymptomatic and NO intervention indicated	Asymptomatic AND non-urgent medical intervention indicated	Symptomatic, non-life-threatening AND non-urgent medical intervention indicated	Life-threatening arrhythmia OR urgent intervention indicated
Ischaemia / infarction	NA	NA	Symptomatic ischaemia (stable angina) OR testing consistent with ischaemia	Unstable angina OR acute myocardial infarction
Haemorrhage (significant acute blood loss)	NA	Symptomatic AND no transfusion indicated	Symptomatic AND transfusion of ≤ 2 units packed RBCs	Life-threatening hypotension OR transfusion of > 2 units packed RBCs
Hypertension<br>(confirmed on repeat testing at same visit)	> 140-159 mmHg systolic<br>OR<br>> 90-99 mmHg diastolic	> 160-179 mmHg systolic<br>OR<br>> 100-109 mmHg diastolic	> 180 mmHg systolic<br>OR<br>> 110 mmHg diastolic	Life-threatening consequences eg. malignant hypertension OR hospitalisation indicated (other than visit to Accident & Emergency dept.)

Hypotension	NA	Symptomatic, corrected with oral fluid replacement	Symptomatic, IV fluids indicated	Shock requiring use of vasopressors or mechanical assistance to maintain blood pressure
Pericardial effusion	Asymptomatic, small effusion requiring no intervention	Asymptomatic, moderate or larger effusion requiring no intervention	Effusion with non-life threatening functional consequences OR effusion with non-urgent intervention indicated	Life-threatening consequences eg. tamponade OR urgent intervention indicated
Thrombosis / embolism	NA	Deep vein thrombosis AND no intervention indicated	Deep vein thrombosis AND intervention indicated (eg. anticoagulation, lysis filter, invasive procedure)	Embolic event (eg. pulmonary embolism, life-threatening thrombus)
Ventricular dysfunction (congestive heart failure)	NA	Asymptomatic (diagnostic finding) AND intervention indicated	New onset symptoms OR worsening symptoms	Life-threatening congestive heart failure
**GASTROINTESTINAL**				
Anorexia	Loss of appetite without decreased oral intake	Loss of appetite associated with decreased oral intake but without significant weight loss	Loss of appetite associated with significant weight loss	Life-threatening consequences OR Aggressive intervention indicated (e.g., tube feeding or total parenteral nutrition)
Diarrhoea	Transient or intermittent episodes of unformed stools OR increase of ≤ 3 stools over baseline per 24 hour period	Persistent episodes of unformed to watery stools OR over baseline per 24 hour period increase 4-6 stools	Bloody diarrhoea OR increase of ≥ 7 stools per 24 hour period OR IV fluid replacement indicated	Life-threatening consequences eg. hypotensive shock
Constipation	NA	Persistent constipation requiring regular use of dietary modifications, laxatives or enemas moderate	Requiring disimpaction	Life-threatening consequences eg. Obstruction
Nausea	Transient (< 24 hours) or intermittent nausea with no or minimal interference with oral intake	Persistent nausea resulting in decreased oral intake for 24-48 hours	Persistent nausea resulting in decreased oral intake for > 48 hours OR aggressive rehydration indicated eg. IV fluids	Life-threatening consequences eg. hypotensive shock
Vomiting	Transient or intermittent vomiting with no or minimal interference with oral intake	Frequent episodes of vomiting with no or mild dehydration	Persistent vomiting resulting in orthostatic hypotension OR aggressive rehydration indicated eg. IV fluids	Life-threatening consequences eg. hypotensive shock

Oral discomfort / Dysphagia	Mild discomfort, able to eat usual diet	Symptoms causing altered dietary intake but no medical intervention indicated	Symptoms causing severely altered dietary intake with medical intervention indicated	Life-threatening reduction in oral intake
**NEUROLOGICAL**				
Alteration in personality / behaviour or mood (eg. Agitation, anxiety, depression, mania, psychosis)	Alteration causing no or minimal interference with ADL	Alteration causing greater than minimal interference with ADL	Alterations causing inability to perform ADL	Behaviour potentially harmful to self or others (eg. Suicidal or homicidal ideation or attempt, acute psychosis) OR causing inability to perform basic self-care
Altered mental status	Changes causing no or minimal interference with ADL	Mild lethargy or somnolence causing greater than minimal interference with ADL	Confusion, memory impairment, lethargy or somnolence causing inability to perform ADL	Delirium OR obtundation OR coma
Ataxia	Asymptomatic ataxia detectable on exam OR minimal ataxia causing no or minimal interference with ADL	Symptomatic ataxia causing greater than minimal interference with ADL	Symptomatic ataxia causing inability to perform ADL	Disabling ataxia causing inability to perform basic self-care
CNS ischaemia (acute)	NA	NA	Transient ischaemic attack	Stroke
Neuromuscular weakness (myopathy & neuropathy)	Asymptomatic with decreased strenght on exam OR minimal muscle weakness causing no or minimal interference with ADL	Muscle weakness causing greater than minimal interference with ADL	Muscle weakness causing inability to perform ADL	Disabling muscle weakness causing inability to perform basic self-care OR Respiratory muscle weakness resulting in ventilator dependence
Neurosensory alteration (including paraesthesiae and painful neuropathy)	Asymptomatic with sensory alteration on exam or minimal paraesthesiae causing no or minimal interference with ADL	Sensory alteration or paraesthesiae causing greater than minimal interference with ADL	Sensory alteration or paraesthesiae causing inability to perform ADL	Disabling sensory alteration or paraesthesiae causing inability to perform basic self-care
**RESPIRATORY**				
Bronchospasm (acute)	Transient; FEV1 or peak flow reduced to 70 - 80%	FEV1 or peak flow 50 - 69%	FEV1 or peak flow 25- 49%	Cyanosis OR requiring intubation OR FEV1 or peak flow < 25%
Dyspnoea	Dyspnoea on exertion with no or minimal interference with ADL	Dyspnoea on exertion with greater than minimal interference with ADL	Dyspnoea at rest causing inability to perform ADL	Respiratory failure with ventilatory support indicated
**MUSCULOSKELETAL**				

Arthritis / arthralgia	Joint stiffness, swelling or pain causing no or minimal interference with ADL	Joint stiffness, swelling or pain causing greater than minimal interference with ADL	Joint stiffness, swelling or pain causing inability to perform ADL	Disabling joint stiffness, swelling or pain causing inability to perform basic self-care
Myalgia (non-injection site)	Muscle pain causing no or minimal interference with ADL	Muscle pain causing greater than minimal interference with ADL	Muscle pain causing inability to perform ADL	Disabling muscle pain causing inability to perform basic self-care
**OCULAR / VISUAL**				
Visual impairment (from baseline)	Visual impairment causing no or minimal interference with ADL	Visual impairment causing greater than minimal interference with ADL	Visual impairment causing inability to perform ADL	Disabling visual loss in affected eye(s)
Uveitis	Asymptomatic but detectable on exam	Symptomatic anterior uveitis OR medical intervention indicated	Posterior or pan-uveitis OR operative intervention indicated	Disabling visual loss in affected eye(s)
**ENDOCRINE / METABOLIC**				
Diabetes mellitus	NA	New onset without need to initiate medication OR modification of current medications to regain glucose control	New onset with initiation of medication indicated OR diabetes uncontrolled despite treatment modification	Life-threatening consequences eg. Ketoacidosis, hyperosmolar non-ketotic coma
Hyperthyroidism<br>OR<br>Hypothyroidism	Asymptomatic	Symptomatic causing greater than minimal interfence with ADL OR thyroid suppression / thyroid replacement therapy indicated	Symptoms causing inability to perform ADL OR uncontrolled despite treatment	Life-threatening consequences (eg. Thyroid storm / myxoedema coma)

**LABORATORY**				
**PARAMETER**	**GRADE 1**	**GRADE 2**	**GRADE 3**	**GRADE 4**
**HAEMATOLOGY**				
Haemoglobin	10.0-10.9 g/dL OR any decrease 2.5-3.4 g/dL	9.0-9.9 g/dL OR any decrease 3.5-4. g/dL	7.0-8.9 g/dL OR any decrease ≥ 4.5 g/dL	< 7.0 g/dL
Absolute neutrophil count	1.0-1.3 x 10^9/L	0.75-0.99 x 10^9/L	0.5-0.74 x 10^9/L	< 0.5 x 10^9/L
WBC, decreased<br>WBC, increased	2.0-2.5 x 10^9/L<br>13.0-14.99 x 10^9/L	1.5-1.99 x 10^9/L<br>15.0-19.99 x 10^9/L	1.0-1.49 x 10^9/L<br>20.0-30.0 x 10^9/L	< 1.0 x 10^9/L<br>> 30.0 x 10^9/L
Platelets, decreased	100.0-124.9 x 10^9/L	50.0-99.9 x 10^9/L	25.0-49.9 x 10^9/L	< 25.0 x 10^9/L
Fibrinogen, decreased	100-200mg/dL<br>OR 0.75-0.99 x LLN	75-99 mg/dL<br>OR 0.5-0.74 x LLN	50-74 mg/dL<br>OR 0.25-0.49 x LLN	< 50 mg/dL OR< 0.25 x LLN OR gross bleeding
Prothrombin time	1.1-1.25 x ULN	1.26-1.5 x ULN	1.51-3.0 x ULN	> 3.0 x ULN

Partial thromboplastin time	1.1-1.66 x ULN	1.67-2.33 x ULN	2.34-3.0 x ULN	> 3.0 x ULN
**CHEMISTRIES**				
Sodium:<br>High<br>Low	 146-150 mmol/L<br>130-135 mmol/L	 151- 154 mmol/L<br>125-129 mmol/L	 155-159 mmol/L<br>121-124 mmol/L	 > 160 mmol/L<br>< 120 mmol/L
Potassium:<br>High<br>Low	 5.6 – 6.0 mmol/L<br>3.0 – 3.4 mmol/L	 6.1 – 6.5 mmol/L<br>2.5 – 2.9 mmol/L	 6.6 – 7.0 mmol/L<br>2.0 – 2.4 mmol/L	 >7.0 mmol/L<br><2.0 mmol/L
Glucose, high (fasting and no prior diabetes)	6.1-6.9 mmol/l	7.0-13.9 mmol/l	14.0-27.9 mmol/l	> 28 mmol/l
Creatinine	1.1-1.3 x ULN	1.4-1.8 x ULN	1.9-3.4 x ULN	≥ 3.5 x UN
ALT	1.25-2.5 x ULN	2.6-5.0 x ULN	5.1-10.0 x ULN	> 10.0 x ULN
ALP	1.25-2.5 x ULN	2.6-5.0 x ULN	5.1-10.0 x ULN	> 10.0 x ULN
Bilirubin	1.25-2.5 x ULN	2.6-5.0 x ULN	5.1-10.0 x ULN	> 10.0 x ULN
GGT	1.25 – 2.5 x ULN	>2.5 – 5.0 x ULN	>5.0 – 10.0 x ULN	> 10.0 x ULN
Calcium (corrected for albumin)<br>High<br>Low	 2.65-2.88 mmol/l<br>1.95-2.10 mmol/l	 2.89-3.13 mmol/l<br>1.75-1.94 mmol/l	 3.14-3.38 mmol/l<br>1.53-1.74 mmol/l	 > 3.38 mmol/l<br>< 1.53 mmol/l
Phosphate	0.81 mmol/l - < LLN	0.65-0.8 mmol/l	0.32-0.64 mmol/l	< 0.32 mmol/l
Uric acid	0.45-0.59 mmol/l	0.6-0.71 mmol/l	0.72-0.89 mmol/l	> 0.89 mmol/l
Lactate	< 2.0 x ULN without acidosis	≥ 2.0 x ULN without acidosis	Increased lactate with pH < 7.3 without life-threatening consequences	Increased lactate with pH < 7.3 with life-threatening consequences
Pancreatic amylase	1.1-1.5 x ULN	1.6-2.0 x ULN	2.1-5.0 x ULN	> 5.0 x ULN
Cardiac troponin I (cTnI)	NA	NA	NA	Levels consistent with myocardial infarction or unstable angina as defined by the manufacturer
Cardiac troponin T (cTnT)	NA	NA	NA	≥ 0.2 ng/mL OR Levels consistent with myocardial infarction or unstable angina as defined by the manufacturer
Creatine kinase	3.0-5.9 x ULN	6.0-9.9 x ULN	10.0-19.9 x ULN	≥ 20.0 x ULN
**URINALYSIS**				
Proteinuria, random collection	1+	2-3+	4+	NA
Haematuria, microscopic	6-10 RBC/hpf	>10 RBC/hpf	Gross, with or without clots OR RBC casts	Transfusion required
